# Fast photochromism of helicene-bridged imidazole dimers†

**DOI:** 10.1039/d4sc03578j

**Published:** 2024-07-19

**Authors:** Katsuya Mutoh, Jiro Abe

**Affiliations:** a Department of Chemistry and Biological Science, College of Science and Engineering, Aoyama Gakuin University Sagamihara Kanagawa 252-5258 Japan jiro_abe@chem.aoyama.ac.jp

## Abstract

The unique optical and magnetic properties of organic biradicaloids on polycyclic aromatic hydrocarbons are of fundamental interest in the development of novel organic optoelectronic materials. Open-shell π-conjugated molecules with helicity have recently attracted a great deal of attention due to the magnetic-field-dependence and spin-selectivity arising from the combination of helical chirality and electron spins. However, the molecular design for helical biradicaloids is limited due to the thermal instability and high reactivity. Herein, we achieved fast photochromic reactions and reversible photo-generation of biradical species using helicene-bridged imidazole dimers. A [9]helicene-bridged imidazole dimer exhibits reversible photochromism upon UV light irradiation. The transient species produced reversibly by UV light irradiation exhibited ESR spectra with a fine structure characteristic of a triplet radical pair, indicating the reversible generation of the biradical. The half-life of the thermal recombination reaction of the biradical was estimated to be 29 ms at 298 K. Conversely, a substantial activation energy barrier was confirmed for the intramolecular recombination reaction in the [7]helicene-bridged imidazole dimer, attributed to the extended pitch length of [7]helicene. The temperature dependence of the thermal back reactions revealed that the [7]helicene and [9]helicene moieties functioned as ‘soft’ and ‘hard’ molecular bridges, respectively. These findings pave the way for future advances in the development of photoswitchable helical biradicaloids.

## Abstract 2

The unique optical and magnetic properties of organic biradicaloids on polycyclic aromatic hydrocarbons are of fundamental interest in the development of novel organic optoelectronic materials.

## Graphical abstract

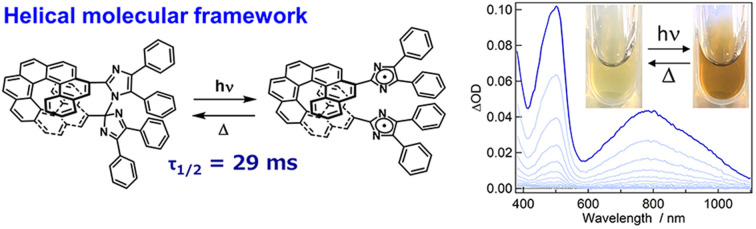

## Introduction

The unique electronic structures and the chemical and physical properties of open-shell biradicaloids have stimulated the interest of chemists in a wide range of research fields.^1–15^ Their applications have also been proposed in nonlinear optical materials,^16–18^ organic field-effect transistors,^19–22^ singlet fission materials (solar cells),^23^ and spintronics.^24^ Recently, various organic biradicaloids based on π-conjugated systems including polycyclic aromatic hydrocarbons have been reported, which are not only fundamental interests of the unique electronic structures but are also among the mainstream materials for the above-mentioned applications.^25–40^ In particular, the combination of helicene chirality and unpaired electron spin within helical π-conjugates has attracted much interest for the discovery of novel organic optoelectronic materials, organic spintronics, and quantum information technology, since the chirality-induced spin selectivity (CISS) was widely recognized and generalized as reported by Naaman *et al.*^41–43^

The robust molecular designs of helical organic monoradicals have been reported thanks to the recent breakthrough of synthetic strategies.^44–52^ However, the biradicaloids embedded in π-conjugated helices remain difficult to synthesize,^53–56^ because the thermal instability of biradicaloids often makes it difficult to synthesize and handle under ambient conditions and to investigate the electronic structures in detail although the chiroptical switches by electrochemical spin-state change have been reported based on the organometallic complexes with helicene.^57–59^ The usual strategies to stabilize organic biradicaloids are kinetic and thermodynamic stabilization by protecting them with bulky substituents and by extending π-conjugation, respectively. The external stimuli and chemical reactions are also effective in the instantaneous generation of organic biradicaloids. While light stimulus presents an attractive perturbation for spatiotemporal control, the typically observed one-way reaction imposes constraints on measurement conditions. Moreover, the application of reversible photoreactions in the study of organic biradicaloids has been constrained.^60–68^ Furthermore, the successful photoswitch of the spin-state in an organic helical molecular framework is scarcely reported, except the only relatively recent report by Dumele *et al.*^69^

**Scheme 1:**
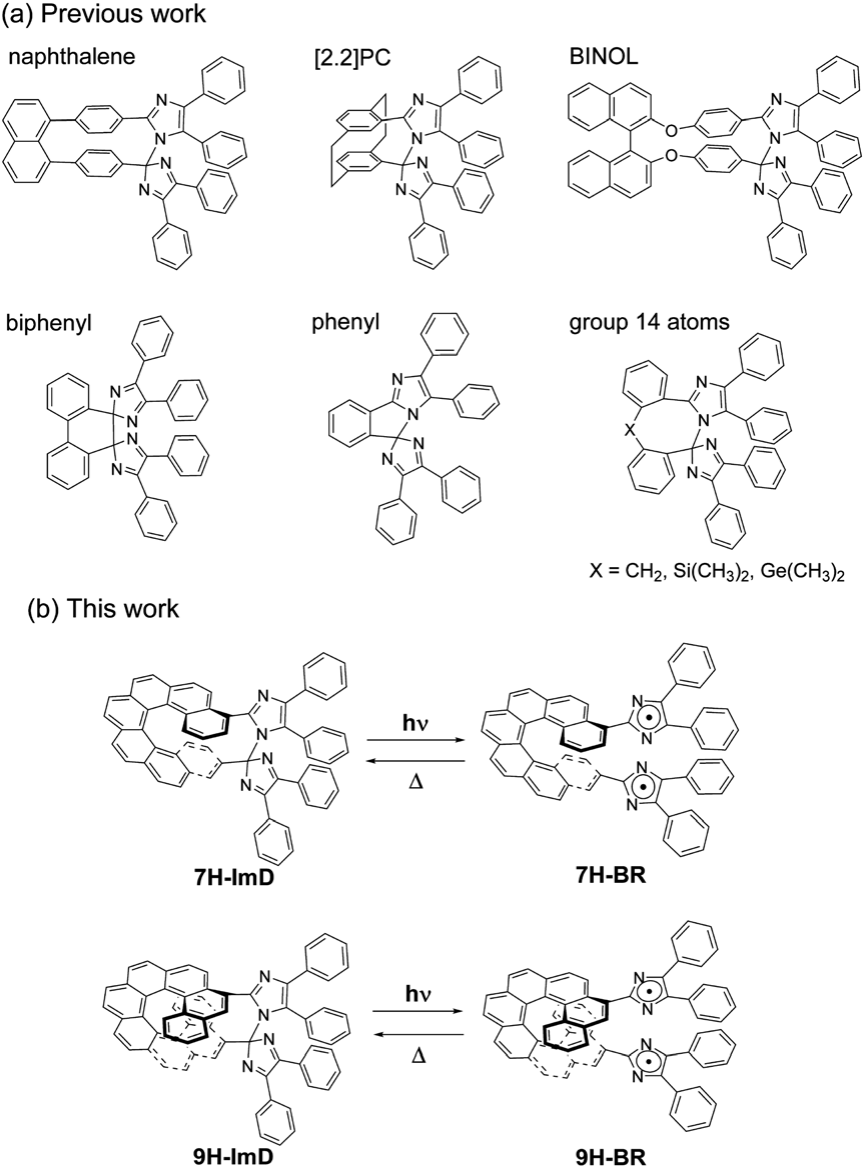
(a) Molecular structures of previously reported bridged imidazole dimers. (b) Photochromic reaction schemes of 7H-ImD and 9H-ImD.

Therefore, in this study, we designed a photochromic molecule that reversibly generates a radical pair bridged by a helicene moiety (Scheme 1). The bridged imidazole dimer shows the C–N bond breaking reaction to form the colored biradical upon light irradiation. The first reported naphthalene-bridged imidazole dimer shows the thermal back reaction of the photogenerated biradical with a half-life of *ca.* 2 s at 295 K.^70^ A [2.2]paracyclophane ([2.2]PC)-bridged imidazole dimer achieved a drastic acceleration of the thermal back reaction to the video frame rate (half-life is 33 ms at 298 K) by bridging the imidazolyl radicals in close proximity.^71^ The other moieties, biphenyl,^72^ phenyl,^73^ and group 14 atoms^74^ were also accepted as the bridging units for the photochromic imidazole dimers, and the thermal back reaction rate could be tuned from nanosecond- to second-time scales. An imidazole dimer bridged by a chiral bridging unit (1,1′-bi-2-naphthol (BINOL)) enables chiroptical switching upon light irradiation.^75^ In addition to the thermal back reaction rate, the optical properties of the transient biradical can be controlled by changing the interaction between the imidazolyl radicals. Due to the pronounced influence of the bridging unit on the photochromic properties of bridged imidazole dimers, the diversity in photochromic performances becomes a compelling and attractive characteristic for photoswitches.^76–81^ In addition, the spatiotemporal photochromic reaction which can selectively generate biradicals by light irradiation is expected to expand the molecular design of biradicals. This capability enables the manipulation of typically unstable biradicals and facilitates the investigation of electronic structures that have not been studied so far. Thus, we focused on the helical structure of helicene derivatives as a novel bridging unit in which the average pitch length can be regulated by the π stacking effect of the aromatic rings. The flexible framework of helicene, behaving like a molecular spring, can alter the distance between the bridged imidazolyl radicals, enabling control over the photochromic properties.

## Results and discussion

### Synthesis

**Scheme 2:**
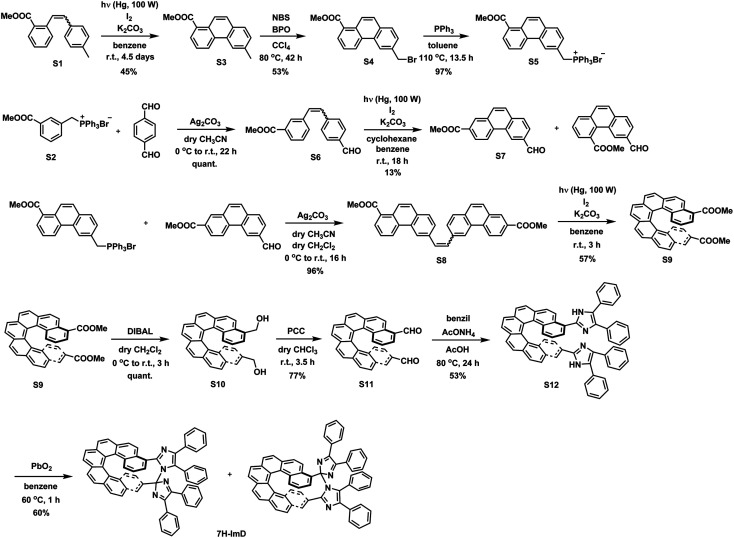
Synthetic scheme of 7H-ImD.

**Scheme 3:**
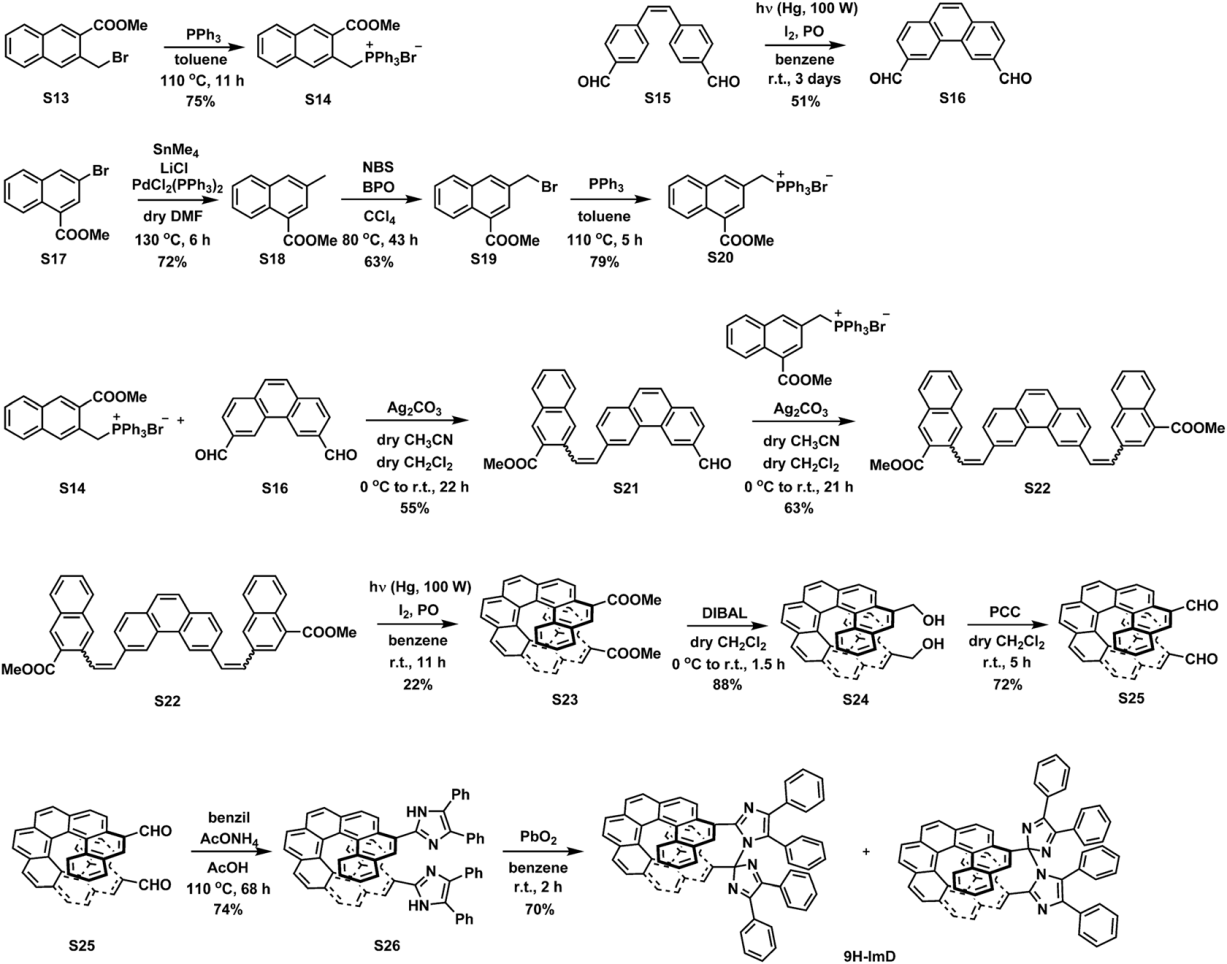
Synthetic scheme of 9H-ImD.

We synthesized the [7]helicene- and [9]helicene-bridged imidazole dimers (7H-ImD and 9H-ImD) according to Schemes 2 and 3, respectively. In order to fix the two imidazolyl radicals in a distance at which they can make a C–N bond between the imidazole rings, the imidazole rings were introduced to the 3- and 15-positions of [7]helicene, and to the 5- and 17-positions of [9]helicene. The helicene-dicarbaldehydes (S11 and S25) are the primary synthetic intermediates to form the imidazole rings. The asymmetric helicene was obtained through the step-by-step Wittig reactions and the subsequent photochemical oxidative cyclization. Under the oxidative photocyclization conditions, potassium carbonate (K_2_CO_3_) or propylene oxide (PO) was used as a scavenger for hydrogen iodide.^82,83^ K_2_CO_3_ is also responsible for the *in situ* generation of potassium iodide that promotes oxidative photocyclization as reported previously.^84^ Although the dicarbaldehyde could be synthesized by lithiation of bromine groups followed by formylation with DMF, the synthetic yields were quite low. On the other hand, the DIBAL reduction of methyl carboxylates followed by oxidation of alcohol smoothly provided the carbaldehyde. The precursor lophine derivatives (S12 and S26) were synthesized by conventional conditions for imidazole condensation with the carbaldehyde. The lophines were oxidized with PbO_2_; especially, S12 was oxidized at 60 °C to reduce the amount of byproducts, as discussed later. There are two types of structural isomers for 7H-ImD and 9H-ImD due to the difference in the bonding manner between the two imidazole rings, due to the asymmetric structure of the bridging helicene, as previously discussed in detail for bridging imidazole dimers.^85^ Because these two structural isomers isomerized each other through an identical photogenerated biradical upon light irradiation, a structural isomer was isolated by the chromatographic technique and used for the spectroscopy. It is noteworthy that the helicene-dicarbaldehyde (S25) can be optically separated by chiral HPLC. The enantiomers of 9H-ImD (P and M isomers) were synthesized by using the corresponding enantiomers of the precursor, and the absolute configuration was estimated by CD spectroscopy (Fig. S59†).

### Optical and photochromic properties

**Fig. 1:**
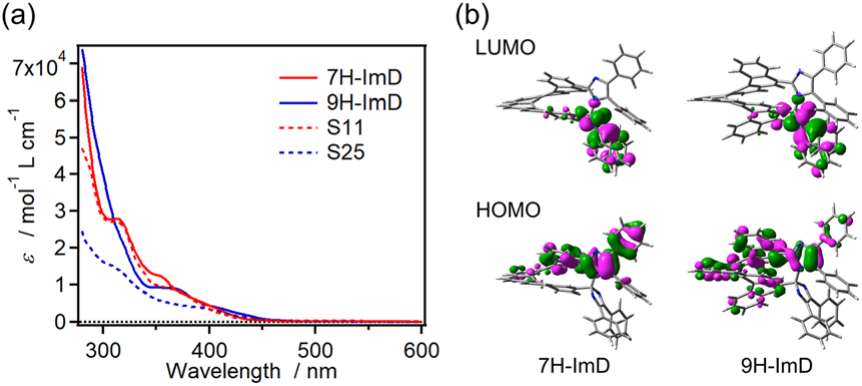
(a) UV-vis absorption spectra of S11, S25, 7H-ImD and 9H-ImD in benzene and (b) frontier Kohn–Sham orbitals of 7H-ImD and 9H-ImD (MPW1PW91/6-31+G(d,p)//M05-2X/6-31G(d)).

The UV-vis absorption spectra of S11, S25, 7H-ImD and 9H-ImD are shown in Fig. 1. The absorption bands of the bridging helicenes were also observed in those of 7H-ImD and 9H-ImD, especially it is characteristic of 7H-ImD. The absorption tails of 7H-ImD and 9H-ImD reached 450 nm. The nature of the lowest unoccupied molecular orbital (LUMO) is crucial for the photochromic reaction of the bridged imidazole dimers. The C–N bonds of 7H-ImD and 9H-ImD exhibit an anti-bonding character at the LUMO similar to those observed in the previously reported photochromic imidazole dimers, this characteristic leads to the bond-breaking reaction of the C–N bonds upon light irradiation.^86^

**Fig. 2:**
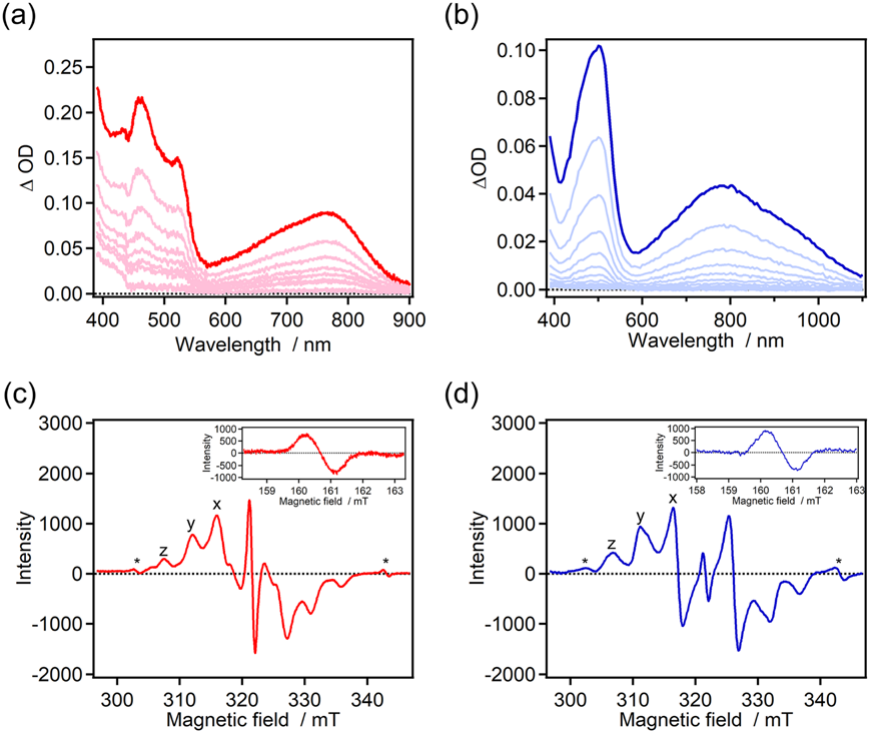
(a and b) Transient absorption spectra of 7H-ImD upon 365 nm CW UV light irradiation (power = 100 mW, 2 s) and 9H-ImD upon 355 nm nanosecond laser irradiation (pulse width = 5 ns, energy = 3 mJ) in benzene at 298 K. (c and d) ESR spectra of 7H-ImD and 9H-ImD in frozen toluene at 80 K upon UV light irradiation (365 nm, 50 mW) (*quartz).

The photochromic properties were investigated by time-resolved absorption spectroscopy (Fig. 2). The color of the benzene solutions of 7H-ImD and 9H-ImD changed from pale yellow to orange in which absorption maxima were observed at around 500 nm and 700–800 nm upon UV light irradiation. The photogenerated species can be attributed to the biradical species (7H-BR and 9H-BR) as discussed later. The absorption spectra of 7H-BR and 9H-BR show the characteristic large and broad absorption bands in the NIR light region. The TDDFT calculation results for 7H-BR and 9H-BR are summarized in the ESI (Fig. S66 and S67†). The charge transfer transition from the bridging helicene unit to the imidazolyl radical mainly contributes to the NIR light absorption (*e.g.*, MO211 → MO213 transition at 713 nm for 7H-BR, UMPW1PW91/6-31+G(d,p)//UM05-2X/6-31G(d) level of the theory),^79^ in addition to the representative optical transition originating from the radical–radical interaction in the face-to-face alignment of the biradical.^71,87,88^ The confirmation of the radical pair generation through UV light irradiation was validated by ESR spectroscopy. The frozen toluene solutions of 7H-ImD and 9H-ImD were irradiated with 365 nm light (50 mW) at 80 K. The characteristic fine structure due to a randomly oriented triplet radical pair and the half-field resonance for the forbidden transition were observed (Fig. 2c and d), indicating the photo-induced homolytic C–N bond breaking reaction in the photochromic reaction. The zero-field splitting parameter (*D*) was determined to be 14.2 mT and 15.0 mT for 7H-BR and 9H-BR, respectively. The distances between the radicals in 7H-BR and 9H-BR were calculated to be 5.83 Å and 5.70 Å, respectively, by means of point-dipole approximation. These distances are similar to those estimated for other photogenerated imidazolyl radical pairs in a frozen matrix or crystal.^89,90^ The slightly longer distance calculated for 7H-BR might be attributed to the difference in the pitch length between [7]helicene and [9]helicene even in the rigid frozen matrix.

**Fig. 3:**
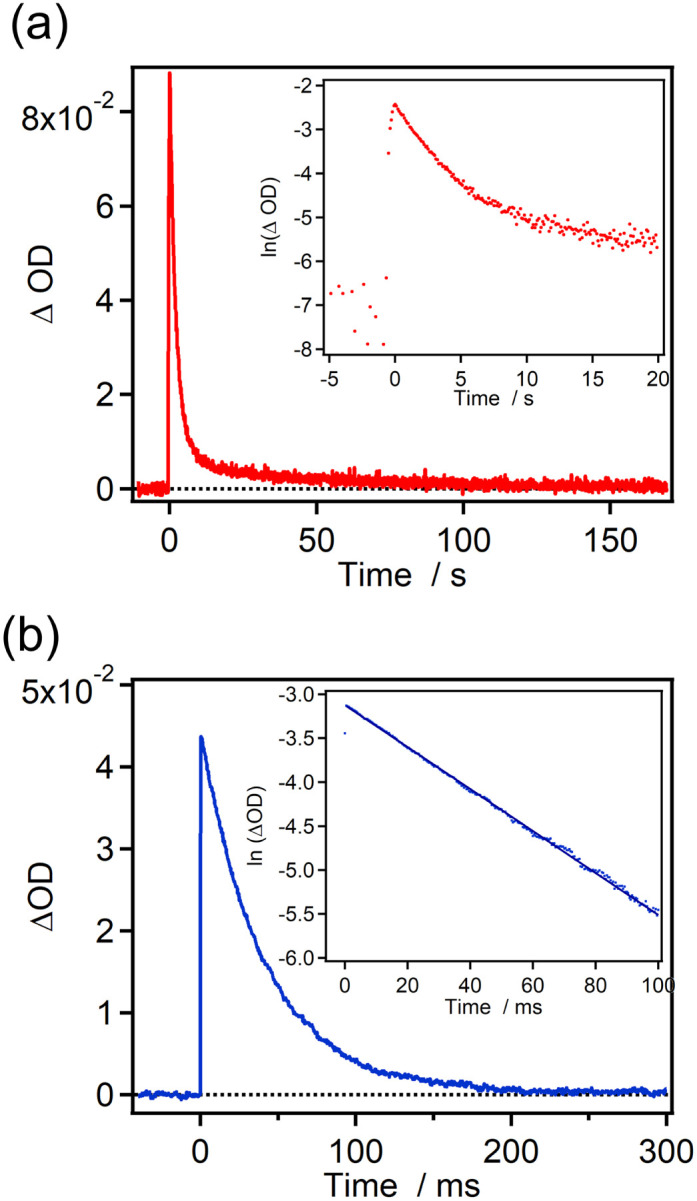
Time profiles of the transient absorbance in benzene at 298 K for (a) 7H-ImD (*λ*_obs._ = 765 nm, *λ*_ex._ = 365 nm, 100 mW) and (b) 9H-ImD (*λ*_obs._ = 800 nm, *λ*_ex._ = 355 nm, 3 mJ). Inset shows the first-order plots for the transient absorbance.

The time profiles of the transient absorbance for 7H-ImD and 9H-ImD are shown in Fig. 3. 9H-ImD exhibits fast photo-switching cycles upon UV light irradiation. The decay of 9H-ImD follows first-order reaction kinetics, and the transient absorption bands revert to the original value on the millisecond time scale (half-life = 29 ms) at 298 K similar to the [2.2]PC-bridged imidazole dimer.^71^ The photochromic reaction of 9H-ImD can be repeated several times (Fig. S62†). In contrast, the transient absorbance of 7H-ImD did not monotonically decrease, and the relatively slow thermal back reaction in the second time scale was observed at 298 K. The remnant absorbance at 400 nm after the thermal back reaction suggests a side reaction path from 7H-BR such that the initial 7H-ImD is not generated. Hence, the HPLC analysis was performed before and after the thermal back reaction to determine the quantity of the reaction byproducts (Fig. S60†). The HPLC peaks corresponding to the initial 7H-ImD, along with four additional byproducts, were observed after 365 nm light irradiation of a benzene solution of 7H-ImD at 298 K. This indicates that the primary reaction pathway of 7H-BR at 298 K involves side reactions from 7H-BR. On the other hand, the generation of the byproducts was suppressed at 333 K, and the time profile of the transient absorbance of 7H-BR could be fitted by the first-order reaction kinetics (Fig. S61†). These results suggest a substantial reaction barrier for the intramolecular recombination reaction between the imidazolyl radicals when bridged with [7]helicene.

**Fig. 4:**
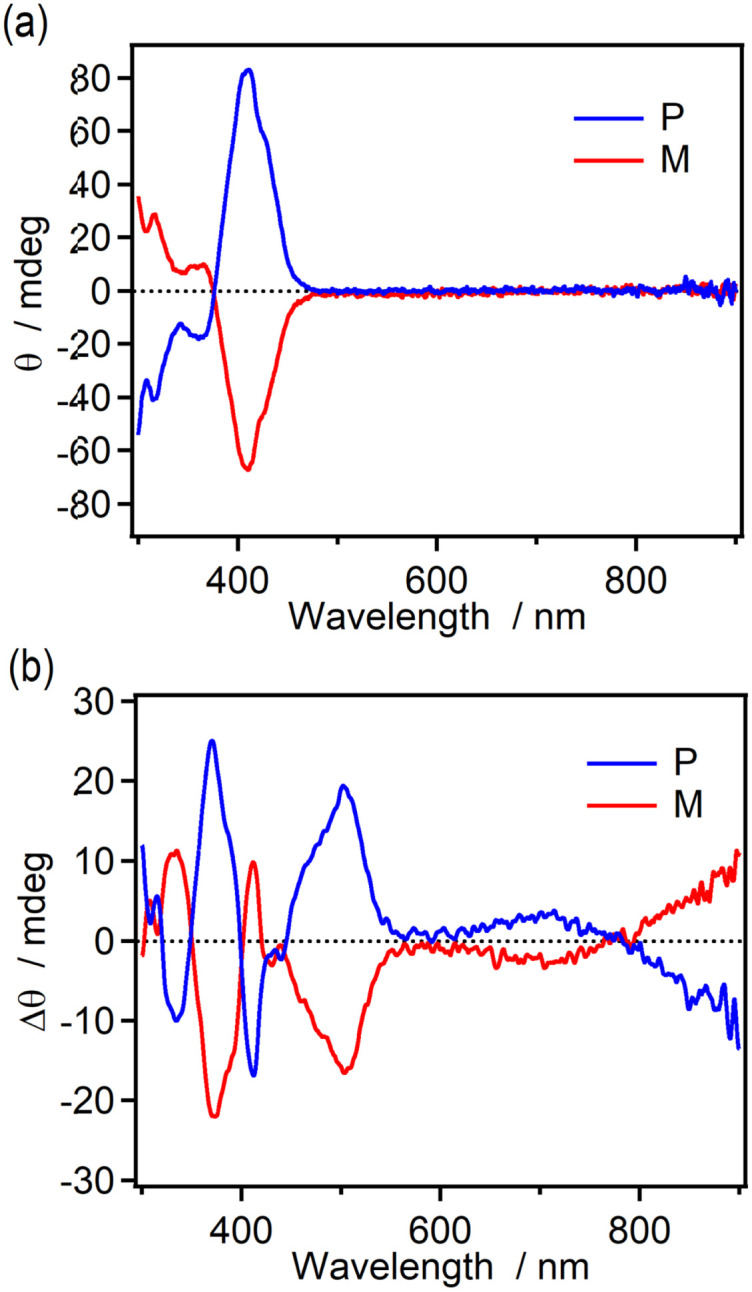
The CD spectra of M- and P-9H-ImD (1.52 × 10^−5^ M and 1.69 × 10^−5^ M, respectively) in toluene at 193 K (a) before and (b) after UV light (365 nm) irradiation.

The chiroptical changes accompanying the photochromic reaction of the enantiomers (M and P isomers) of 9H-ImD in toluene were demonstrated by CD spectroscopy at 193 K (Fig. 4). The CD spectra for the enantiomers show the characteristic Cotton effect around 400 nm derived from the helicene units as also shown in Fig. S59.† After UV light irradiation of 9H-ImD at 193 K, new Cotton effects were observed at 500 nm and 600–900 nm. Since these peaks are consistent with the characteristic absorption bands of 9H-BR, the chirality of the helicene bridges is propagated to the biradicals. It is known that the chiral excitonic coupling between the two chromophores induces the splitting of the CD signals into opposite signs. Because the absorption band at 800 nm of 9H-BR is attributable to the radical–radical interaction in the face-to-face alignment of the biradical, the change in the sign of the Cotton effect from 900 nm to 700 nm would indicate the chiral arrangement and the chiral excitonic interaction between the two imidazolyl radicals of 9H-BR.

### Activation parameters for the thermal back reaction

The significant temperature-dependent variation in the rate of side reactions suggests a substantial contribution of the entropy term to the Gibbs energy of the thermal back reaction. Therefore, the activation enthalpy (Δ*H*^‡^) and entropy (Δ*S*^‡^) for the thermal back reactions of 7H-ImD and 9H-ImD were estimated from the Eyring plots of the rate constants at several temperatures (Fig. S63 and S64†). In these temperature ranges, we could not observe the thermochromic biradical generation. The Eyring plots produced excellent straight lines, and the Δ*H*^‡^ and Δ*S*^‡^ were estimated from standard least-square analysis (Table 1). The activation Gibbs energies (Δ*G*^‡^) for 7H-ImD and 9H-ImD were calculated to be 76.2 and 65.1 kJ mol^−1^, respectively. The substantial reaction barrier for 7H-ImD is attributed to the large negative value of Δ*S*^‡^. The significant difference in the entropy value between the 7H-BR state and the transition state suggests considerable structural change during the thermal back reaction. We also previously reported that the Δ*S*^‡^ value reflects the structural flexibility of the bridging unit.^87,91^ The distances between the bridging points of [7]helicene and [9]helicene were estimated to be 4.4 Å and 3.7 Å, respectively, based on the reported crystal structures (CCDC 852537 and 1051158). Consequently, the long pitch length of [7]helicene increases the distance between the photogenerated radicals of 7H-BR, resulting in the large activation barrier for the recombination and the slow thermal back reaction. Thus, one of the origins of the large negative value of Δ*S*^‡^ would be the long distance between the bridging points of 7H-ImD, which induces the large conformation change during the thermal back reaction. Moreover, the molecular vibration facilitating recombination of the radicals would be induced by thermal energy at high temperatures as indicated by the reduced formation of byproducts at 333 K. From these results, it is inferred that the rates of the thermal back reactions and the Δ*S*^‡^ values for 7H-ImD and 9H-ImD indicate that [7]helicene bridges the imidazolyl radicals with flexibility, whereas [9]helicene tightly bridges them. The flexibility of the molecular framework as a molecular spring has been discussed from the force constant calculated by the DFT calculations for helicene derivatives.^92,93^ According to the literature, the relaxed potential energy surface was scanned from the equilibrium structure (Fig. S68†). The force constants (*k*) for [7]helicene and [9]helicene were calculated to be 2.0 and 2.7 N m^−1^, respectively, by fitting the potential energy curves using the function of Δ*E*_el_ = 1/2*k*(Δ*r*)^2^. The larger force constant of [9]helicene compared to [7]helicene suggests the rigid molecular structure of [9]helicene which is not in conflict with the results from the Eyring analyses. Hence, it would be considered that the [7]helicene and [9]helicene moieties serve as ‘soft’ and ‘hard’ molecular springs, respectively, while further theoretical and experimental investigations will be required for the precise discussion about the molecular springs.

**Table 1:** Activation parameters and the half-life for the thermal back reactions estimated by Eyring analysis

	Δ*H*^‡^ (kJ mol^−1^)	Δ*S*^‡^ (J mol^−1^ K^−1^)	Δ*G*^‡^ (kJ mol^−1^)	*τ*_1/2_
7H-ImD	42.2	−114	76.2	2.5 s
9H-ImD	52.3	−42.5	65.1	29 ms

## Conclusions

We examined the photochromic behavior of imidazole dimers bridged by aromatic helicene units (7H-ImD or 9H-ImD). It was revealed that these molecules reversibly generate the biradical species upon UV light irradiation as evidenced by time-resolved absorption spectroscopy and ESR spectroscopy. Although 9H-ImD shows the fast thermal recombination reaction of the biradical and the reversible photochromism in a millisecond time scale at 298 K, the biradical of 7H-ImD undergoes several side reactions due to the slow thermal recombination reaction at 298 K. The Eyring analysis for the thermal back reactions revealed the significant negative value of Δ*S*^‡^ for 7H-ImD, indicating the large structural change to form the C–N bond between the imidazole rings of 7H-BR. The reversibility of the photochromic reaction of 7H-ImD was improved by increasing the temperature. Because the bridging [7]helicene possesses flexibility akin to molecular springs, the heat induces molecular vibrations, enhancing the frequency and bringing the two imidazolyl radicals closer to a distance suitable for recombination. Furthermore, the helicene-bridged imidazole dimer can reversibly generate the biradical on the helically twisted molecular structure upon light irradiation. By utilizing optically separated helicene-dicarbaldehyde derivatives, the enantiomer can be prepared, making this study on photo-responsive helical imidazole dimers a novel molecular design and principle for future biradicaloid development. These strides will aid in uncovering spin interactions within helically twisted chiral molecules.

## Data availability

The data supporting this article have been included as part of the ESI.†

## Author contributions

K. M.: conceptualization, data curation, funding acquisition, investigation, validation, visualization, writing – original draft, writing – review & editing. J. A.: conceptualization, funding acquisition, resources, supervision, writing – review & editing.

## Conflicts of interest

There are no conflicts to declare.

## Supplementary Material

SC-015-D4SC03578J-s001
