# Supplementary material for: Fast photochromism of helicene-bridged imidazole dimers
Source: Chem Sci. 2024 Jul 19;15(33):13343–50. doi: 10.1039/d4sc03578j (PMC11339945; doi:10.1039/d4sc03578j)
Supplement: SC-015-D4SC03578J-s001 [file SC-015-D4SC03578J-s001.pdf]

Electronic Supplementary Material (ESI) for Chemical Science.
This journal is © The Royal Society of Chemistry 2024

Electronic Supplementary Information for:

Fast Photochromism of Helicene-Bridged Imidazole Dimers

Katsuya Mutoh[a,b] and Jiro Abe*[a]

[a] *Department of Chemistry and Biological Science, College of Science and Engineering, Aoyama Gakuin University, Sagamihara, Kanagawa 252-5258, Japan*
[b] *(Present address) Department of Chemistry, Graduate School of Science, Osaka Metropolitan University, Sumiyoshi, Osaka 558-8585, Japan.*

E–mail: jiro_abe@chem.aoyama.ac.jp

CONTENTS

1. Syntheses **S2**
2. NMR Spectra **S8**
3. HR-ESI-TOF MS Spectra **S27**
4. HPLC Chromatograms **S34**
5. CD Spectroscopy **S35**
6. Transient Absorption Spectroscopy **S36**
7. Eyring Analysis **S37**
8. DFT Calculation **S39**
9. References **S75**

1. Syntheses

All reactions were monitored by thin-layer chromatography carried out on 0.2 mm E. Merck silica gel plates (60F-254). Column chromatography was performed on silica gel (Silica gel 60N, Kanto Chemical). ^{1}H NMR spectra were recorded at 400 MHz on a Bruker AVANCE III 400 NanoBay. $CDCl_3$, THF-d_8, and DMSO-d_6 were used as deuterated solvents. ESI–TOF–MS spectra were recorded on a Bruker micrOTOF II-AGA1. All glassware was washed with distilled water and dried. A phase separator paper (Whatman® 1PS) was used to dry the organic solution. Unless otherwise noted, all reagents and reaction solvents were purchased from TCI, Wako Co. Ltd., Aldrich Chemical Co., Inc. and Kanto Chemical Co., Inc. and were used without further purification.

Methyl-2-(4-methylstyryl)benzoate (S1) was synthesized according to the literatures.[S1]

(3-(Methoxycarbonyl)benzyl)triphenylphosphonium bromide (S2) was synthesized according to the literatures.[S2]

Methyl-6-methylphenanthrene-1-carboxylate (S3)

A solution of methyl 2-(4-methylstyryl)benzoate (932 mg, 3.69 mmol), iodine (932 mg, 3.67 mmol) and K_2CO_3 (6.00 g, 43.4 mmol) in cyclohexane (650 mL) was stirred at room temperature for 4.5 days under light irradiation with a high pressure Hg lamp (100 W). The reaction was quenched by adding excess $NaHSO_3$ powder and the solid was removed by filtration. The organic layer was removed by evaporation. The residue was separated by SiO_2 column chromatography (hexane:CH_2Cl_2 = 1:1) to give the desired product as a pale yellow solid (416 mg, yield: 45%). ^{1}H NMR (400 MHz, $CDCl_3$) δ: 8.91 (d, J = 8.4 Hz, 1H), 8.69 (d, J = 9.6 Hz, 1H), 8.45 (s, 1H), 8.19 (dd, J = 7.6, 1.2 Hz, 1H), 7.84–7.81 (m, 2H), 7.65 (t, J = 8.0 Hz, 1H), 7.47 (dd, J = 8.0, 1.2 Hz, 1H), 4.03 (s, 3H), 2.64 (s, 3H). ^{13}C NMR (100 MHz, $CDCl_3$) δ: 168.5, 136.7, 130.7, 130.6, 130.0, 129.6, 129.5, 128.8, 128.6, 128.3, 128.1, 127.1, 125.0, 122.7, 122.5, 52.3, 22.2. HRMS (ESI-TOF): calculated for $C_{17}H_{14}O_2$ $[M+Na]^+$: 273.0886, found: 273.0887.

Methyl-6-(bromomethyl)phenanthrene-1-carboxylate (S4)

A mixture of methyl-6-methylphenanthrene-1-carboxylate (350 mg, 1.40 mmol), NBS (270 mg, 1.52 mmol) and benzoyl peroxide (25% wet, 30 mg, 0.093 mmol) in CCl_4 (10 mL) was stirred at 80 °C for 42 h. The organic layer was washed with water and brine, and passed through a phase separator paper. After the evaporation of the solvent, the residue was separated by SiO_2 column chromatography (hexane/CH_2Cl_2 = 2/1) to give the desired product as a colorless solid (244 mg, yield: 53%). ^{1}H NMR (400 MHz, $CDCl_3$) δ: 8.91 (d, J = 8.4 Hz, 1H), 8.80 (d, J = 9.2 Hz, 1H), 8.70 (s, 1H), 8.24 (dd, J = 7.6, 1.2 Hz, 1H), 7.91 (d, J = 8.4 Hz, 1H), 7.86 (d, J = 9.2 Hz, 1H), 7.72–7.66 (m, 2H), 4.79 (s, 2H), 4.04 (s, 3H). ^{13}C NMR (100 MHz, $CDCl_3$) δ: 168.3, 136.2, 131.4, 130.8, 130.6, 130.1, 130.0, 129.2, 128.28, 128.26, 127.9, 127.1, 125.5, 124.5, 123.3, 52.4, 34.2. HRMS (ESI-TOF): calculated for $C_{17}H_{13}BrO_2$ $[M+Na]^+$: 350.9991, found: 350.9975.

((8-(Methoxycarbonyl)phenanthren-3-yl)methyl)triphenylphosphonium bromide (S5)

A mixture of methyl 6-(bromomethyl)phenanthrene-1-carboxylate (200 mg, 0.608 mmol) and PPh_3 (180 mg, 0.686 mmol) in toluene (5 mL) was stirred at 110 °C for 13.5 h. The precipitate was collected and washed with toluene and Et_2O to give the desired product as a colorless solid (349 mg, yield: 97%). ^{1}H NMR (400 MHz, DMSO-d_6) δ: 8.57 (d, J = 9.6 Hz, 1H), 8.40 (s, 1H), 8.37 (d, J = 8.4 Hz, *2*H), 8.16 (dd, J = 7.2, 0.8 Hz, 1H), 7.97–7.88 (m, 5H), 7.78–7.72 (m, 13H), 7.35 (d, J = 8.4 Hz, 1H), 5.42 (d, J = 15.6 Hz, 2H), 3.97 (s, 3H). ^{13}C NMR (100 MHz, $CDCl_3$) δ: 134.74, 134.67, 134.64, 130.31, 130.27, 130.1, 130.01, 129.95, 129.8, 129.7, 129.6, 129.5, 129.24, 129.21, 128.10, 128.07, 127.8, 127.6, 127.5, 127.3, 127.0, 126.9, 125.6, 125.5, 125.3, 123.9, 118.3, 117.4, 53.5, 52.2, 30.7, 30.3. HRMS (ESI-TOF): calculated for $C_{35}H_{28}O_2P$ $[M]^+$: 511.1821, found: 511.1844.

Methyl 3-(4-formylstyryl)benzoate (S6) & Methyl 6-formylphenanthrene-2-carboxylate (S7)

A mixture of (3-(methoxycarbonyl)benzyl)triphenylphosphonium bromide (1.00 g, 2.04 mmol) and terephthalaldehyde (270 mg, 2.01 mmol) in dry CH_3CN (35 mL) was stirred at 0 °C. To the solution was added Ag_2CO_3 (561 mg, 2.04 mmol) at 0 °C and the mixture was stirred at r.t. for 22 h. After filtration with celite, the solvent was evaporated, and the residue was separated by SiO_2 column chromatography (CH_2Cl_2) to give pale yellow oil (the mixture of the *cis*- and *trans*-isomers, 548 mg, quant.). This was used for the next reaction step without further purification.

A solution of methyl 3-(4-formylstyryl)benzoate (490 mg, 1.84 mmol), iodine (730 mg, 2.88 mmol) and K_2CO_3 (3.00 g, 21.7 mmol) in cyclohexane (700 mL) and benzene (10 mL) was stirred at room temperature for 18 h under light irradiation with a high-pressure Hg lamp (100 W). The reaction was quenched by adding excess $NaHSO_3$ powder and the solid was removed by filtration. The organic layer was removed by evaporation. The residue was separated by SiO_2 column chromatography (CH_2Cl_2) two times, and recrystallized from CH_2Cl_2/hexane by liquid-liquid diffusion at room temperature to give the desired product as a colorless solid (62 mg, isolated yield: 13%). ^{1}H NMR (400 MHz, $CDCl_3$) δ: 10.30 (s, 1H), 9.22 (s, 1H), 8.84 (d, J = 8.8 Hz, 1H), 8.67 (d, J = 1.6 Hz, 1H), 8.35 (dd, J = 8.8, 1.6 Hz, 1H), 8.15 (dd, J = 8.0, 1.6 Hz, 1H), 8.05 (d, J = 8.0 Hz, 1H), 7.99 (d, J = 9.2 Hz, 1H), 7.87 (d, J = 8.8 Hz, 1H), 4.03 (s, 3H). ^{13}C NMR (100 MHz, $CDCl_3$) δ: 192.2, 166.8, 136.8, 134.5, 133.4, 131.6, 131.1, 130.6, 129.7, 129.6, 128.8, 127.6, 127.34, 127.30, 126.2, 123.0, 52.4. HRMS (ESI-TOF): calculated for $C_{17}H_{12}O_3$ $[M+H]^+$: 265.0859, found: 265.0872.

Methyl 6-(2-(7-(methoxycarbonyl)phenanthren-3-yl)vinyl)phenanthrene-1-carboxylate (S8)

A mixture of ((8-(Methoxycarbonyl)phenanthren-3-yl)methyl)triphenylphosphonium bromide (150 mg, 0.254 mmol) and methyl 6-formylphenanthrene-2-carboxylate (62 mg, 0.235 mmol) in dry CH_3CN (12 mL) and dry CH_2Cl_2 (8 mL) was stirred at 0 °C. To the solution was added Ag_2CO_3 (70 mg, 0.254 mmol) at 0 °C and the mixture was stirred at r.t. for 16 h. After filtration with celite, the solvent was evaporated, and the residue was separated by SiO_2 column chromatography (CH_2Cl_2) to give the desired product as a pale yellow solid (the mixture of the *cis*- and *trans*-isomers, 112 mg, yield: 96%). This was used for the next reaction without further purification. HRMS (ESI-TOF): calculated for $C_{34}H_{24}O_4$ $[M+Na]^+$: 519.1567, found: 519.1558.

[7]Helicene-di(methylcarboxylate) (S9)

A solution of methyl 6-(2-(7-(methoxycarbonyl)phenanthren-3-yl)vinyl)phenanthrene-1-carboxylate (100 mg, 0.201 mmol), iodine (207 mg, 0.816 mmol) and K_2CO_3 (437 mg, 3.16 mmol) in benzene (700 mL) was stirred at room temperature for 3 h under light irradiation with a high pressure Hg lamp (100 W). The reaction was quenched with $Na_2S_2O_3$ and the solid was removed by filtration. The organic layer was removed by evaporation. The residue was separated by SiO_2 column chromatography (CH_2Cl_2) to give the desired product as a yellow solid (57 mg, yield: 57%). ^{1}H NMR (400 MHz, $CDCl_3$) δ: 8.44 (d, J = 9.6 Hz, 2H), 8.06–8.04 (m, *4*H), 8.00 (d, J = 1.6 Hz, 1H), 7.96–7.90 (m, *2*H), 7.83 (d, J = 8.8 Hz, 1H), 7.75 (d, J = 8.8 Hz, 1H), 7.54–7.49 (m, 2H), 7.35 (d, J = 8.4 Hz, 1H), 7.17 (d, J = 8.4 Hz, 1H), 7.01 (dd, J = 8.8, 2.0 Hz, 1H), 6.38 (t, J = 8.0 Hz, 1H), 3.91–3.90 (m, 6H). ^{13}C NMR (100 MHz, $CDCl_3$) δ: 167.9, 166.8, 132.2, 132.1, 132.0, 131.8, 130.8, 130.5, 130.0, 129.8, 129.4, 128.7, 128.5, 128.1, 127.7, 127.6, 127.5, 127.4, 127.33, 127.26, 127.2, 127.1, 127.0, 126.6, 126.1, 126.0, 125.3, 124.9, 124.6, 123.7, 123.4, 122.1, 52.0. HRMS (ESI-TOF): calculated for $C_{34}H_{22}O_4$ $[M+Na]^+$: 517.1410, found: 517.1393.

[7]Helicene-dimethanol (S10) & [7]Helicene-dicarbaldehyde (S11)

A solution of [7]helicene-di(methylcarboxylate) (55 mg, 0.111 mmol) in dry CH_2Cl_2 (6 mL) was dropwise added DIBAL-H (1.0 M/CH_2Cl_2, 0.5 mL, 0.500 mmol) at 0 °C and stirred at room temperature for 3 h. The reaction mixture was quenched with 1N

HCl aq. and extracted with CH_2Cl_2. The organic layer was washed with water and brine. The organic layer was removed by evaporation to give the desired product as an orange amorphous (50 mg, quant.). This was used without further purification for the next reaction. ^{1}H NMR (400 MHz, $CDCl_3$) δ: 8.01–7.99 (m, 4H), 7.97–7.90 (m, 3H), 7.82 (d, *J* = 8.4 Hz, 1H), 7.72 (d, *J* = 8.8 Hz, 1H), 7.47 (d, *J* = 8.4 Hz, 1H), 7.27 (s, 1H), 7.17 (d, *J* = 8.4 Hz, 1H), 7.12 (d, *J* = 8.4 Hz, 1H), 6.85 (d, *J* = 6.8 Hz, 1H), 6.41 (dd, *J* = 8.4, 1.6 Hz, 1H), 6.31 (t, *J* = 7.6 Hz, 1H), 5.15 (d, *J* = 12.0 Hz, 1H), 4.61 (d, *J* = 11.6 Hz, 1H), 4.52–4.45 (m, *2*H).

The entire batch of crude [7]helicene-dimethanol (50 mg, 0.114 mmol), dissolved in dry CH_2Cl_2 (6 mL) under N_2, was added PCC (54 mg, 0.251 mmol) and celite (54 mg). The mixture was stirred at r.t. for 3.5 h and filtered through a short plug of silica gel. The organic layer was removed by evaporation to give the desired product as a yellow solid (38 mg, yield: 77%). ^{1}H NMR (400 MHz, $CDCl_3$) δ: 10.13 (s, 1H), 9.80 (s, 1H), 8.82 (dd, *J* = 8.8, 0.4 Hz, 1H), 8.10–8.06 (m, 4H), 7.99–7.92 (m, 3H), 7.81 (d, *J* = 8.8 Hz, 1H), 7.76 (d, *J* = 0.4 Hz, 1H), 7.58 (d, *J* = 8.4 Hz, 1H), 7.44 (d, *J* = 8.4 Hz, 1H), 7.38 (dd, *J* = 7.2, 1.2 Hz, 1H), 7.21 (d, *J* = 8.8 Hz, 1H), 6.86 (dd, *J* = 8.8, 1.6 Hz, 1H), 6.51 (t, *J* = 8.0 Hz, 1H). ^{13}C NMR (100 MHz, $CDCl_3$) δ: 192.6, 191.5, 133.8, 132.82, 132.76, 132.43, 132.42, 132.3, 131.3, 131.0, 130.82, 130.75, 130.0, 129.6, 129.5, 129.0, 128.6, 127.8, 127.7, 127.5, 127.4, 127.24, 127.18, 127.1, 125.2, 124.8, 124.7, 122.9, 122.5, 121.9. HRMS (ESI-TOF): calculated for $C_{32}H_{18}O_2$ $[M+H]^+$: 435.1380, found: 435.1395.

7H-ImDL (S12)

A solution of [7]helicene-dicarbaldehyde (15 mg, 0.0345 mmol), benzil (20 mg, 0.0951 mmol) and ammonium acetate (45 mg, 0.584 mmol) in acetic acid (1 mL) was stirred at 80 °C for 24 h. The solution was neutralized with NH_3 aq. and extracted with CH_2Cl_2. The organic layer was washed with water and brine, passed through a phase separator paper, and the solvent was removed by evaporation. The residue was separated by SiO_2 column chromatography (from CH_2Cl_2 to CH_2Cl_2/AcOEt=20/1) and washed with CH_2Cl_2/hexane to give the desired product as a yellow solid (15 mg, yield: 53%). ^{1}H NMR (400 MHz, DMSO-d_6) δ: 12.49 (s, 1H), 12.42 (s, 1H), 9.01 (d, *J* = 8.8 Hz, 1H), 8.25–8.12 (m, 7H), 8.00–7.95 (m, 2H), 7.70 (d, *J* = 8.8 Hz, 1H), 7.51–7.49 (m, 2H), 7.40–7.17 (m, 18H), 7.12–7.07 (m, 4H), 6.53 (t, *J* = 8.0 Hz, 1H). ^{13}C NMR (100 MHz, $CDCl_3$) δ: 146.1, 146.0, 132.6, 132.5, 132.0, 131.4, 130.9, 130.3, 129.8, 129.3, 128.5, 128.4, 128.2, 128.1, 128.03, 127.98, 127.9, 127.63, 127.61, 127.52, 127.49, 127.3, 127.2, 127.1, 127.0, 126.8, 126.5, 126.3, 126.0, 125.5, 125.4, 124.9, 124.7, 124.1, 123.1, 121.1. HRMS (ESI-TOF): calculated for $C_{60}H_{38}N_4$ $[M+H]^+$: 815.3169, found: 815.3161.

7H-ImD

A mixture of 7H-ImDL (5 mg, 0.0061 mmol) and PbO_2 (50 mg) in benzene (1 mL) was degassed by N_2 bubbling and stirred at 60 °C for 1 h. PbO_2 was filtered off with celite and the solvent was evaporated. The residue was separated by PTLC (CH_2Cl_2/AcOEt = 20/1) to give the desired product as a yellow solid (3 mg, yield: 60%). ^{1}H NMR (400 MHz, THF-d_8) δ: 7.98–7.96 (m, 4H), 7.90–7.82 (m, 3H), 7.71 (d, *J* = 8.8 Hz, 1H), 7.61 (d, *J* = 8.4 Hz, 1H), 7.50 (d, *J* = 8.4 Hz, 1H), 7.46–7.42 (m, 2H), 7.39–7.23 (m, 15H), 7.19 (d, *J* = 8.8 Hz, 1H), 7.17–7.07 (m, 3H), 7.04–6.96 (m, 3H), 6.80 (dd, *J* = 8.8, 2.0 Hz, 1H), 6.44 (t, *J* = 7.6 Hz, 1H). ^{13}C NMR (100 MHz, THF-d_8) δ: 168.6, 165.5, 147.0, 139.0, 135.7, 134.2, 133.9, 133.83, 133.80, 133.5, 132.9, 132.83, 132.75, 132.6, 132.4, 132.3, 132.1, 131.4, 131.31, 131.27, 131.24, 130.8, 130.6, 130.4, 129.1, 128.9, 128.7, 128.5, 128.3, 128.2, 128.1, 127.9, 127.8, 127.6, 127.49, 127.48, 127.42, 127.3, 127.1, 126.7, 126.4, 126.18, 126.15, 125.8, 124.5, 122.1, 121.8, 119.2, 113.3. HRMS (ESI-TOF): calculated for $C_{60}H_{36}N_4$ $[M+H]^+$: 813.3013, found: 813.3016.

Methyl 3-(bromomethyl)-2-naphthoate (S13) was synthesized according to the literature procedure.[S3]
(Z)-4,4'-(Ethene-1,2-diyl)dibenzaldehyde (S15) was synthesized according to the literature procedure.[S4]

Methyl 3-bromo-1-naphthoate (S17) was synthesized according to the literature procedure.[S5]

((3-(Methoxycarbonyl)naphthalen-2-yl)methyl)triphenylphosphonium bromide (S14)

A mixture of methyl 3-(bromomethyl)-2-naphthoate (10.97 g, 39.29 mmol) and PPh_3 (12.00 g, 45.75 mmol) in toluene (250 mL) was stired at 110 °C for 11 h. After cooling to room temperature, the precipitate was collected and washed with toluene and Et_2O to give the desired product as a colorless solid (15.94 g, yield: 75%). ^{1}H NMR (400 MHz, DMSO-d_6) δ: 8.56 (s, 1H), 8.13 (d, J = 7.6 Hz, 1H), 7.93–7.88 (m, 4H), 7.80 (d, J = 7.6 Hz, 1H), 7.75–7.57 (m, 14H), 5.67 (d, J = 15.2 Hz, 2H), 3.50 (s, 3H). ^{13}C NMR (100 MHz, DMSO-d_6) δ: 166.3, 135.12, 135.09, 134.1, 134.0, 133.2, 132.43, 132.35, 131.49, 131.47, 130.2, 130.1, 129.7, 129.2, 128.0, 127.2, 126.2, 126.1, 124.9, 124.8, 117.9, 117.0, 52.2, 27.3, 26.8. HRMS (ESI-TOF): calculated for $C_{31}H_{26}O_2P$ $[M]^+$: 461.1665, found: 461.1667.

Phenanthrene-3,6-dicarbaldehyde (S16)

A solution of (Z)-4,4'-(ethene-1,2-diyl)dibenzaldehyde (2.52 g, 10.7 mmol), iodine (3.26 g, 12.8 mmol) and propylene oxide (PO) (18.0 mL, 257 mmol) in benzene (1.4 L) was stirred at room temperature for 3 days under light irradiation with a high pressure Hg lamp (100 W). The solvent was evaporated, and the residue was separated by SiO_2 column chromatography (CH_2Cl_2) to give the desired product as a pale yellow solid (1.28 g, yield: 51%). ^{1}H NMR (400 MHz, $CDCl_3$) δ: 10.34 (s, 2H), 9.30 (s, 2H), 8.17 (dd, J = 8.4, 1.2 Hz, 2H), 8.08 (d, J = 8.4, 2H), 7.97 (s, 2H). ^{13}C NMR (100 MHz, $CDCl_3$) δ: 192.1, 136.1, 134.9, 130.4, 129.9, 129.7, 126.9, 126.0. HRMS (ESI-TOF): calculated for $C_{16}H_{10}O_2$ $[M+H]^+$: 235.0754, found: 235.0746.

Methyl 3-methyl-1-naphthoate (S18)

To a solution of methyl 3-bromo-1-naphthoate (7.70 g, 29.0 mmol) in dry DMF (150 mL) was added LiCl (6.30 g, 149 mmol) and $SnMe_4$ (5.43 mL, 39.1 mmol) at room temperature. The solution was degassed by N_2 bubbling, and added $PdCl_2(PPh_3)_2$ (1.00 g, 1.43 mmol), and then stirred at 130 °C for 6 h. After cooling to room temperature, the reaction was quenched with H_2O and 1N aqueous HCl. The crude mixture was extracted with EtOAc and the combined organic extracts were washed with 1N aqueous HCl and brine, and concentrated in vacuo. The residue was purified by column chromatography (silica gel/K_2CO_3 (10 wt%), hexane/CH_2Cl_2 = 2/1) to give the desired product as pale yellow oil (4.16 g, yield: 72%). ^{1}H NMR (400 MHz, $CDCl_3$) δ: 8.83 (d, J = 8.8 Hz, 1H), 8.03 (d, 2.0 Hz, 1H), 7.80–7.79 (m, 2H), 7.56–7.47 (m, 2H), 4.00 (s, 3H), 2.54 (s, 3H). ^{13}C NMR (100 MHz, $CDCl_3$) δ: 168.1, 134.2, 134.1, 132.4, 132.3, 129.6, 127.9, 126.9, 126.8, 126.2, 125.6, 52.1, 21.4. HRMS (ESI-TOF): calculated for $C_{13}H_{12}O_2$ $[M+Na]^+$: 223.0730, found: 223.0723.

Methyl 3-(bromomethyl)-1-naphthoate (S19)

A mixture of methyl 3-(methyl)-1-naphthoate (4.26 g, 21.3 mmol), NBS (4.09 g, 23.0 mmol) and BPO (25% wet, 266 mg, 1.10 mmol) in CCl_4 (60 mL) was stired at 80 °C for 43 h. The organic layer was washed with water and brine and evaporated. The residue was separated by SiO_2 column chromatography (hexane/CH_2Cl_2 = 2/1) to give the desired product as a colorless solid (3.77 g, yield: 63%). ^{1}H NMR (400 MHz, $CDCl_3$) δ: 8.89 (d, J = 8.8 Hz, 1H), 8.22 (d, J = 2.0 Hz, 1H), 8.01 (s, 1H), 7.87 (d, J = 8.4 Hz, 1H), 7.63 (dt, J = 7.2, 1.6 Hz, 1H), 7.56 (dt, J = 6.8, 1.2 Hz, 1H), 4.67 (s, 2H), 4.02 (s, 3H). ^{13}C NMR (100 MHz, $CDCl_3$) δ: 167.4, 133.9, 133.8, 132.9, 131.1, 130.9, 128.6, 128.4, 128.0, 126.8, 125.8, 52.3, 32.9. HRMS (ESI-TOF): calculated for $C_{13}H_{11}O_2Br$ $[M+H]^+$: 279.0015, found: 279.0024.

((4-(Methoxycarbonyl)naphthalen-2-yl)methyl)triphenylphosphonium bromide (S20)

A mixture of methyl 3-(bromomethyl)-1-naphthoate (3.75 g, 13.4 mmol) and PPh_3 (4.10 g, 15.6 mmol) in toluene (85 mL) was stirred at 110 °C for 5 h. The precipitate was collected and washed with toluene and Et_2O to give the desired product as a colorless solid (5.76 g, yield: 79%). ^{1}H NMR (400 MHz, $CDCl_3$) δ: 8.70 (d, J = 8.8 Hz, 1H), 8.03 (s, 1H), 7.80–7.73 (m, 9H), 7.63–7.58 (m, 7H), 7.53–7.48 (m, 2H), 7.40 (t, J = 7.2 Hz, 1H), 5.62 (d, J = 14.4 Hz, 2H), 3.80 (s, 3H). ^{13}C NMR (100 MHz, $CDCl_3$) δ: 166.9, 136.8, 136.7, 135.00, 134.97, 134.4, 134.3, 133.6, 133.5, 132.13, 132.10, 130.4, 130.3, 130.2, 130.1, 128.7, 128.4, 127.0, 126.9, 126.7, 125.3, 123.5, 123.4, 117.9, 117.1, 52.0, 30.8, 30.3. HRMS (ESI-TOF): calculated for $C_{31}H_{26}O_2P$ $[M]^+$: 461.1665, found: 461.1651.

Methyl 3-(2-(6-formylphenanthren-3-yl)vinyl)-2-naphthoate (S21)

A mixture of ((3-(Methoxycarbonyl)naphthalen-2-yl)methyl)triphenylphosphonium bromide (1.18 g, 2.18 mmol) and phenanthrene-3,6-dicarbaldehyde (500 mg, 2.13 mmol) in dry CH_3CN (83 mL) and dry CH_2Cl_2 (83 mL) was stirred at 0 °C. To the solution was then added Ag_2CO_3 (880 mg, 3.19 mmol) at 0 °C. The mixture was stirred at room temperature for 22 h. After filtration, the solvent was evaporated, and the residue was separated by SiO_2 column chromatography (CH_2Cl_2) to give the desired product as a yellow solid (the mixture of the *cis*- and *trans*-isomers, 497 mg, yield: 55%). This was used for the next reaction step without further purification. HRMS (ESI-TOF): calculated for $C_{29}H_{20}O_3$ $[M+H]^+$: 417.1485, found: 417.1467.

Methyl 3-(2-(6-(2-(3-(methoxycarbonyl)naphthalen-2-yl)vinyl)phenanthren-3-yl)vinyl)-1-naphthoate (S22) and **Dimethyl [9]helicene-dicarboxylate (S23)**

A mixture of methyl 3-(2-(6-formylphenanthren-3-yl)vinyl)-2-naphthoate (440 mg, 1.06 mmol) and ((4-(methoxycarbonyl)naphthalen-2-yl)methyl)triphenylphosphonium bromide (610 mg, 1.13 mmol) in dry CH_3CN (70 mL) and dry CH_2Cl_2 (70 mL) was stirred at 0 °C. To the solution was then added Ag_2CO_3 (330 mg, 1.20 mmol) at 0 °C. The mixture was stirred at room temperature for 21 h. After filtration, the solvent was evaporated, and the residue was separated by SiO_2 column chromatography (hexane:CH_2Cl_2 = 1:2) to give the desired product **S22** as a yellow solid (the mixture of the structural isomers, 397 mg, yield: 63%). This was used for the next reaction step without further purification.

A solution of methyl 3-(2-(6-(2-(3-(methoxycarbonyl)naphthalen-2-yl)vinyl)phenanthren-3-yl)vinyl)-1-naphthoate (397 mg, 0.663 mmol), iodine (250 mg, 0.985 mmol) and propylene oxide (830 mg, 14.3 mmol) in benzene (700 mL) was stirred at room temperature for 11 h under light irradiation with a high pressure Hg lamp (100 W). The solvent was removed by evaporation. The residue was separated by SiO_2 column chromatography (CH_2Cl_2) to give the desired product as an orange solid (88 mg, yield: 22%). ^{1}H NMR (400 MHz, $CDCl_3$) δ: 8.33 (d, J = 8.0 Hz, 1H), 8.21 (d, J = 8.4 Hz, 1H), 8.00–7.94 (m, 4H), 7.82 (dd, J = 8.0, 2.0 Hz, 2H), 7.46 (d, J = 8.0 Hz, 1H), 7.41 (d, J = 8.0 Hz, 2H), 7.20–7.11 (m, 4H), 7.08–6.99 (m, 3H), 6.46 (dt, J = 6.8, 1.2 Hz, 1H), 6.39 (dt, J = 6.8, 1.2 Hz, 1H), 4.10 (s, 3H), 4.06 (s, 3H). ^{13}C NMR (100 MHz, $CDCl_3$) δ: 168.2, 167.7, 133.0, 131.8, 131.5, 131.4, 131.2, 130.9, 129.7, 129.1, 128.02, 127.95, 127.59, 127.57, 127.3, 127.2, 127.1, 126.90, 126.87, 126.7, 126.54, 126.47, 126.3, 126.0, 126.1, 125.6, 125.5, 125.4, 125.1, 125.0, 124.7, 124.30, 124.26, 124.1, 123.6, 123.5, 123.4, 52.2, 52.0. HRMS (ESI-TOF): calculated for $C_{42}H_{26}O_4$ $[M+Na]^+$: 617.1723, found: 617.1730.

[9]helicene-dimethanol (S24) and **[9]helicene-dicarbaldehyde (S25)**

To a solution of [9]helicene-dicarboxylate (88 mg, 0.15 mmol) in dry CH_2Cl_2 (9 mL) was dropwise added 1.0 M DIBAL-H (0.760 mL, 0.760 mmol) at 0 °C and stirred at room temperature for 1.5 h. The reaction mixture was quenched with 1N HCl aq. and extracted with CH_2Cl_2. The organic layer was washed with 1N HCl aq. and brine. The organic layer was removed by evaporation to

give the desired product as a yellow solid (70 mg, yield: 88%). This was used for the next reaction step without further purification. ^{1}H NMR (400 MHz, $CDCl_3$) δ: 7.98 (s, 2H), 7.84–7.79 (m, 2H), 7.52–7.33 (m, 9H), 7.18–7.15 (m, 2H), 7.11 (s, 2H), 7.06–6.98 (m, 3H), 6.41 (t, J = 7.2 Hz, 1H), 6.36 (t, J = 7.2 Hz, 1H), 5.22 (d, J = 14.0 Hz, 1H), 5.09 (d, J = 12.8 Hz, 1H), 4.98 (d, J = 13.6 Hz, 1H), 4.82 (d, J = 12.8 Hz, 1H).

A mixture of [9]helicene-dimethanol (70 mg, 0.13 mmol), PCC (75 mg, 0.35 mmol) and celite (75 mg) in dry CH_2Cl_2 (15 mL) was stirred at r.t. for 5 h and filtered through a short plug of silica gel and celite. The filtrate was evaporated to give the desired product as a yellow solid (50 mg, yield: 72%). The M and P isomers were separated by chiral chromatography (DAICEL, CHIRALPAK IC, eluent: CH_2Cl_2/hexane/TH*F*= 1/1/2) (Fig. S58). The absolute configuration was determined by the CD spectroscopy and the DFT calculation. ^{1}H NMR (400 MHz, $CDCl_3$) δ: 10.28 (s, 1H), 10.24 (s, 1H), 8.73 (dd, J = 8.8, 1.2 Hz, 1H), 8.44 (d, J = 8.8 Hz, 1H), 8.02 (d, J = 8.0 Hz, 1H), 8.00 (d, J = 8.0 Hz, 1H), 7.89–7.85 (m, 3H), 7.68 (s, 1H), 7.54–7.45 (m, 3H), 7.25–7.07 (m, 7H), 6.56 (t, J = 8.0 Hz, 1H), 6.47 (t, J = 8.0 Hz, 1H). ^{13}C NMR (100 MHz, $CDCl_3$) δ: 192.6, 192.3, 138.0, 137.8, 133.7, 132.2, 131.3, 131.1, 130.0, 129.9, 129.8, 129.6, 129.4, 128.3, 128.2, 128.0, 127.8, 127.6, 127.5, 127.3, 127.02, 126.96, 126.93, 126.79, 126.77, 126.65, 126.55, 126.50, 126.4, 126.2, 125.4, 124.3, 124.2, 124.1, 123.60, 123.58, 123.4, 122.6. HRMS (ESI-TOF): calculated for $C_{40}H_{22}O_2$ $[M+Na]^+$: 557.1512, found: 557.1530.

(M)- or (P)-[9]helicene-ImDL (S26)

A solution of [9]helicene-dicarbaldehyde (M or P: 15 mg, 0.028 mmol), benzil (40 mg, 0.19 mmol) and ammonium acetate (100 mg, 1.30 mmol) in acetic acid (11 mL) was stirred at 110 °C for 68 h. The solution was neutralized with NH_3 aq. and extracted with CH_2Cl_2. The organic layer was washed with water and brine, and dried through a phase separator paper, and the solvent was removed by evaporation. The residue was purified by SiO_2 column chromatography (CH_2Cl_2/AcOEt = 20/1) to give the desired product as yellow amorphous (M: 15 mg, yield: 58%, P: 19 mg, yield: 74%). ^{1}H NMR (400 MHz, DMSO-d_6) δ: 12.35 (s, 1H), 12.26 (s, 1H), 8.95 (d, J = 8.4 Hz, 1H), 8.70 (d, J = 7.6 Hz, 1H), 8.16 (d, J = 8.4 Hz, 1H), 8.13 (d, J = 8.4 Hz, 1H), 8.10 (s, 1H), 8.02 (d, J = 8.0 Hz, 1H), 7.98 (d, J = 8.0 Hz, 1H), 7.79 (s, 1H), 7.63 (d, J = 8.4 Hz, 1H), 7.58 (d, J = 8.4 Hz, 1H), 7.45–7.38 (m, 5H), 7.30–7.24 (m, 11H), 7.21–7.15 (m, 6H), 7.12–7.02 (m, 5H), 6.98 (d, J = 8.4 Hz, 1H), 6.39–6.33 (m, 2H). ^{13}C NMR (100 MHz, $CDCl_3$) δ: 145.7, 145.6, 134.9, 134.5, 132.5, 131.9, 131.3, 131.2, 130.9, 130.5, 129.9, 129.1, 129.0, 128.8, 128.3, 128.2, 127.8, 127.6, 127.4, 127.3, 127.13, 127.11, 127.0, 126.93, 126.88, 126.83, 126.75, 126.71, 126.67, 126.6, 126.5, 126.0, 125.64, 125.55, 125.3, 125.1, 124.9, 124.7, 124.4, 123.9, 123.7, 123.4, 123.2. HRMS (ESI-TOF): calculated for $C_{68}H_{42}N_4$ $[M+H]^+$: 915.3482, found: 915.3469.

(M)- or (P)-[9]helicene-ImD

A mixture of [9]helicene-bis(diphenylimidazole) (M or P : 10 mg, 0.011 mmol) and PbO_2 (40 mg) in benzene (5 mL) was stirred at r.t. for 2 h. PbO_2 was filtered off with celite and the solvent was evaporated. The residue was separated by aminated PTLC (CH_2Cl_2/AcOEt = 60/1) to give the desired product as a yellow solid (M or P: 7 mg, yield: 70%). ^{1}H NMR (400 MHz, THF-d_8) δ: 9.47 (d, J = 8.8 Hz, 1H), 8.07 (d, J = 8.4 Hz, 1H), 8.04 (d, J = 8.0 Hz, 1H), 7.98–7.68 (m, 5H), 7.67 (d, J = 8.0 Hz, 2H), 7.60 (dd, J = 8.0, 1.2 Hz, 2H), 7.57–7.54 (m, 2H), 7.50–7.43 (m, 3H), 7.39–7.28 (m, 5H), 7.26–7.21 (m, 2H), 7.18–6.99 (m, 14H), 6.40 (dt, J = 7.2, 1.2 Hz, 1H), 6.29 (t, J = 7.2 Hz, 1H). ^{13}C NMR (100 MHz, THF-d_8) δ: 167.7, 165.4, 148.4, 138.5, 135.6, 134.9, 134.7, 134.16, 134.13, 133.6, 133.20, 133.16, 133.0, 132.8, 132.7, 131.6, 131.5, 131.44, 131.38, 131.28, 131.25, 131.2, 131.0, 130.9, 130.2, 130.0, 129.41, 129.35, 239.13, 129.07, 129.0, 128.9, 128.6, 128.5, 128.32, 128.28, 128.1, 128.00, 127.96, 127.9, 127.82, 127.79, 127.7, 127.6, 127.5, 127.42, 127.38, 126.9, 126.4, 126.0, 125.8, 125.64, 125.60, 124.2, 122.7, 122.4, 122.2, 114.6. HRMS (ESI-TOF): calculated for $C_{68}H_{40}N_4$ $[M+H]^+$: 913.3326, found: 913.3335.

2. NMR Spectra

^{1}H NMR Spectra

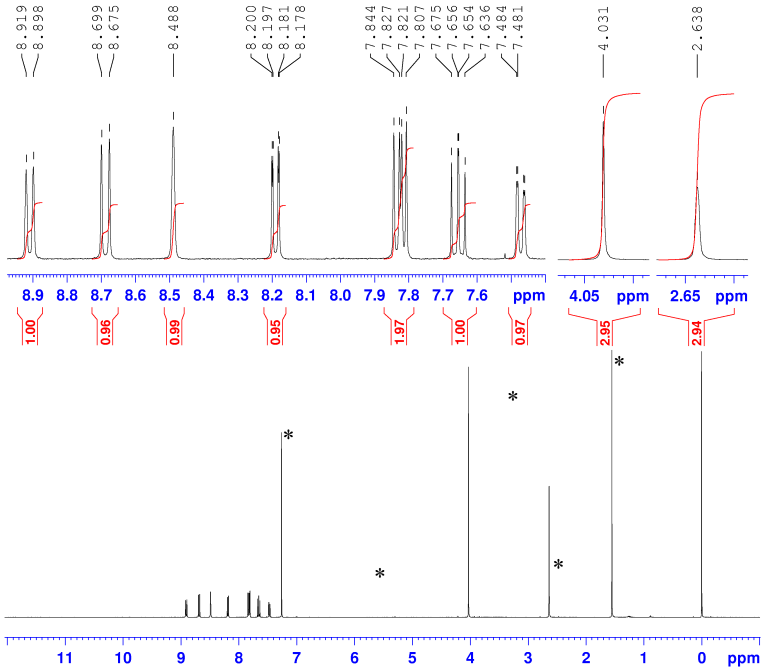

Fig. S1 ^{1}H NMR spectrum of **methyl-6-methylphenanthrene-1-carboxylate (S3)** in $CDCl_3$ (* solvent peaks).

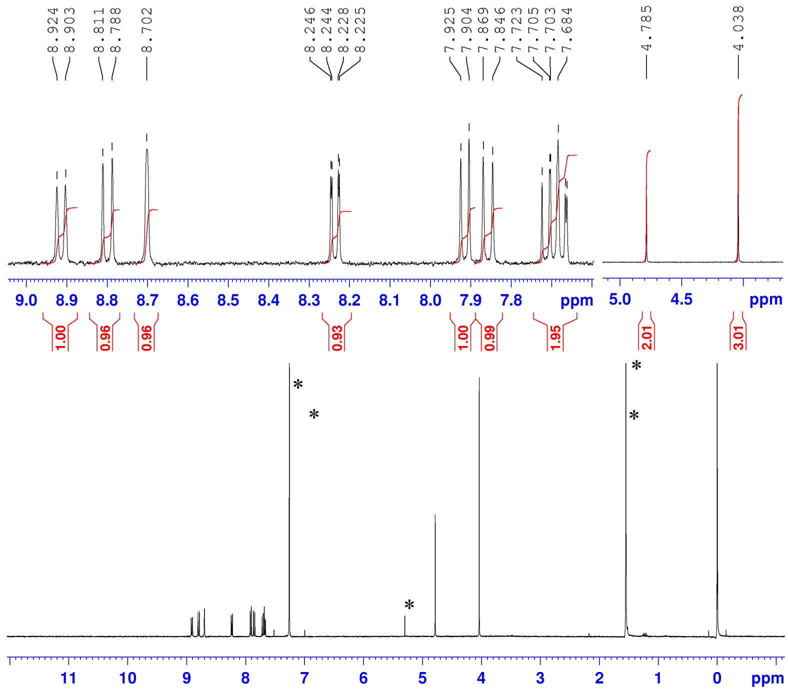

Fig. S2 ^{1}H NMR spectrum of **methyl-6-(bromomethyl)phenanthrene-1-carboxylate (S4)** in $CDCl_3$ (* solvent peaks).

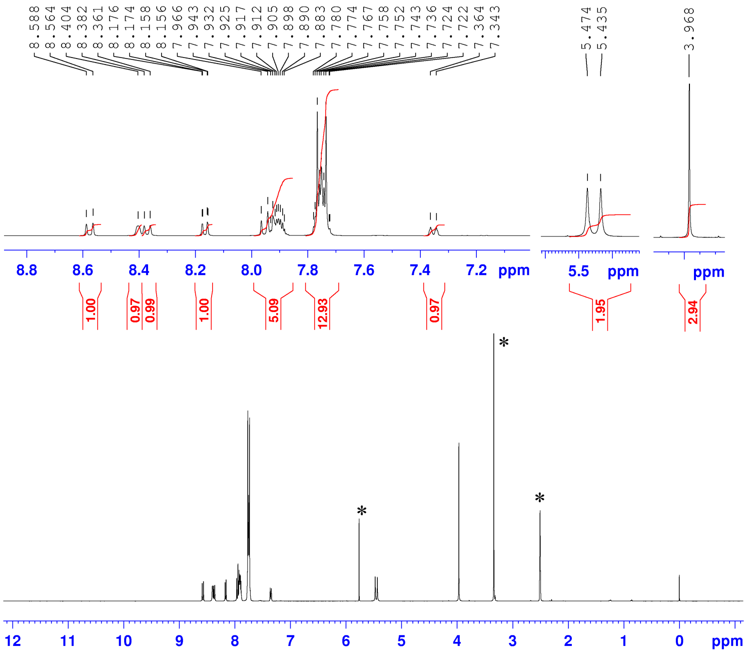

Fig. S3 ^{1}H NMR spectrum of **((8-(methoxycarbonyl)phenanthren-3-yl)methyl)triphenylphosphonium bromide (S5)** in DMSO-d_6 (* solvent peaks).

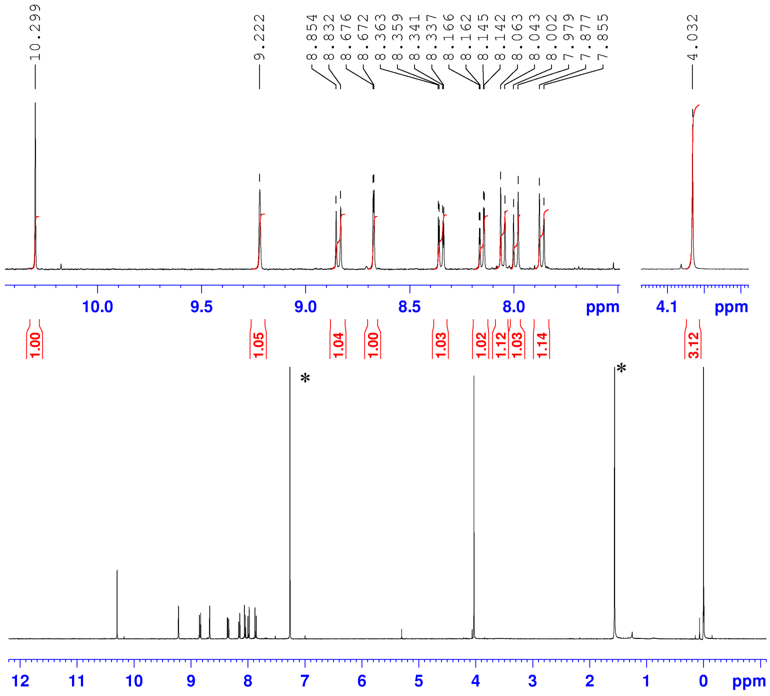

Fig. S4 ^{1}H NMR spectrum of **methyl 6-formylphenanthrene-2-carboxylate (S7)** in $CDCl_3$ (* solvent peaks).

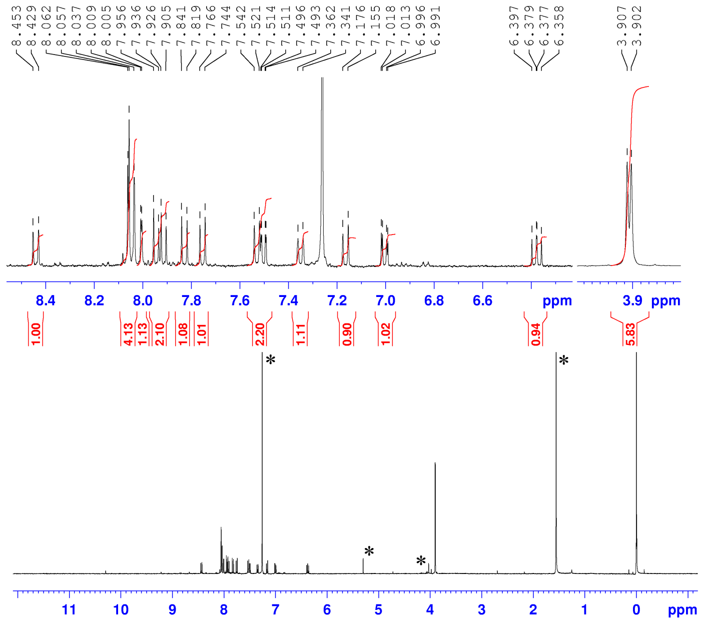

Fig. S5 ^{1}H NMR spectrum of **[7]helicene-di(methylcarboxylate) (S9)** in $CDCl_3$ (* solvent peaks).

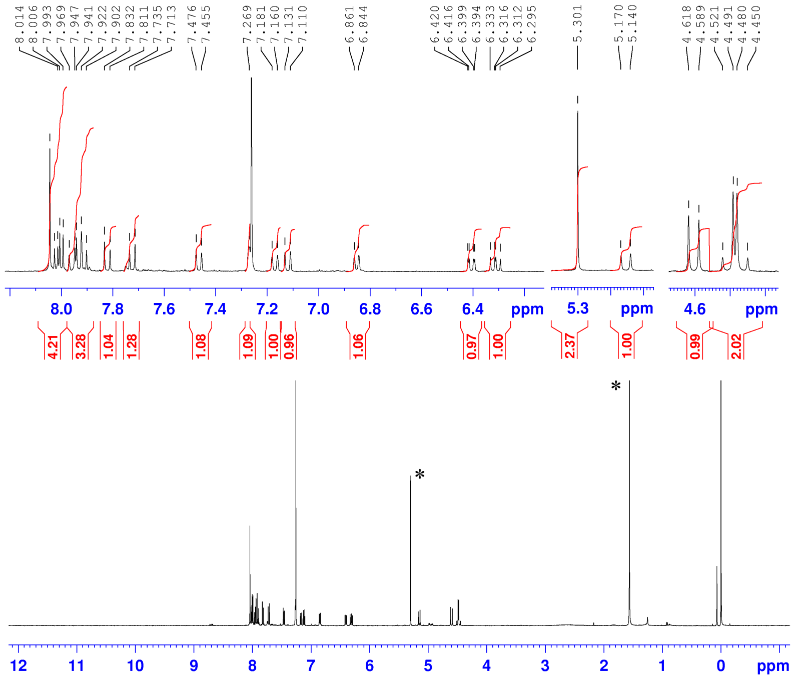

Fig. S6 ^{1}H NMR spectrum of **[7]helicene-dimethanol (S10)** in $CDCl_3$ (* solvent peaks).

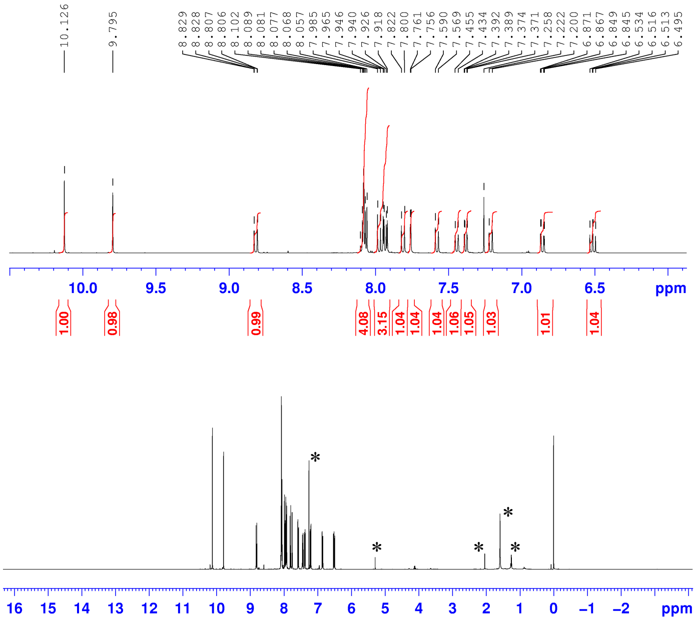

Fig. S7 ^{1}H NMR spectrum of **[7]helicene-dicarbaldehyde (S11)** in $CDCl_3$ (* solvent peaks).

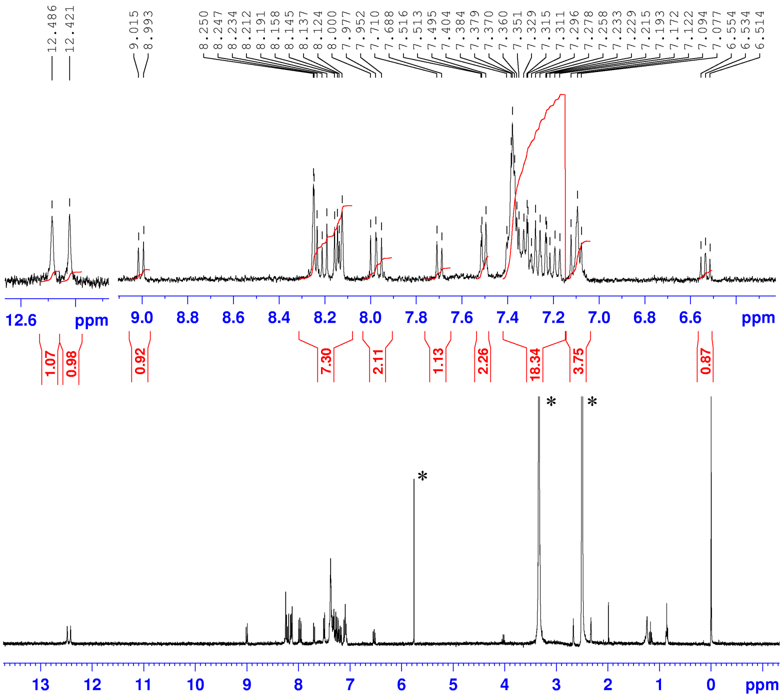

Fig. S8 ^{1}H NMR spectrum of **7H-ImDL (S12)** in DMSO-d_6 (* solvent peaks).

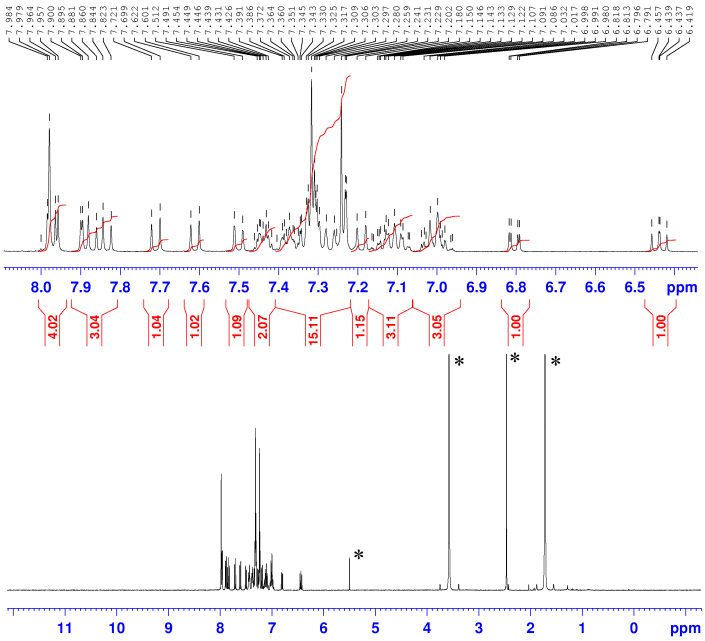

Fig. S9 ^{1}H NMR spectrum of **7H-ImD** in THF-d_8 (* solvent peaks).

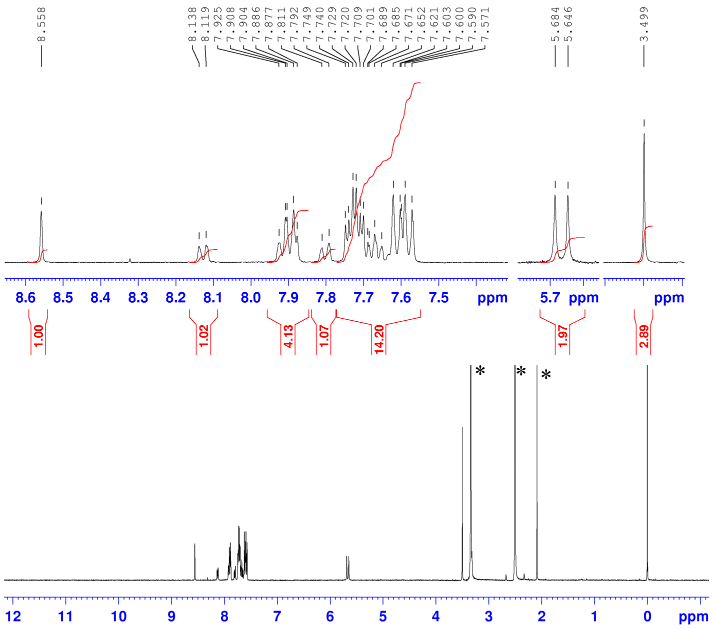

Fig. S10 ^{1}H NMR spectrum of **((3-(methoxycarbonyl)naphthalen-2-yl)methyl)triphenylphosphonium bromide (S14)** in DMSO-d_6 (* solvent peaks).

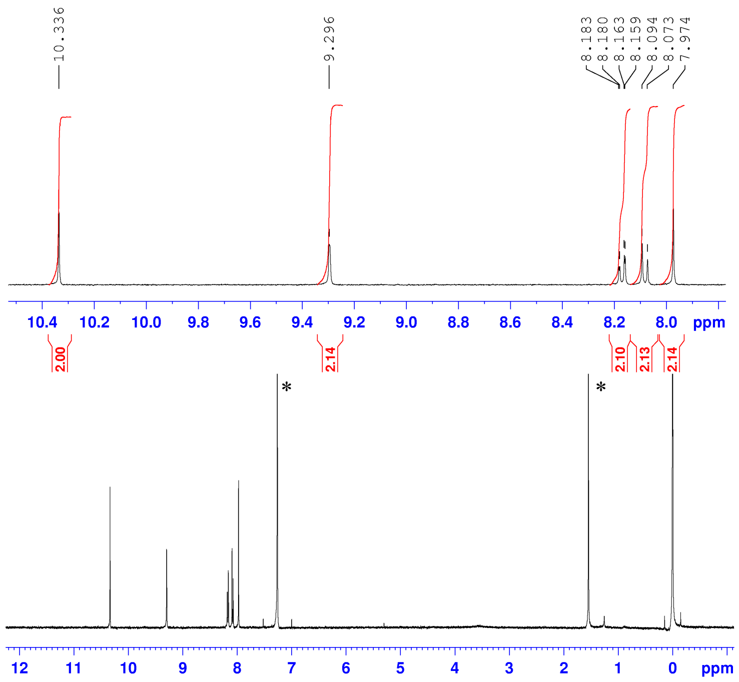

Fig. S11 ^{1}H NMR spectrum of **phenanthrene-3,6-dicarbaldehyde (S16)** in $CDCl_3$ (* solvent peaks).

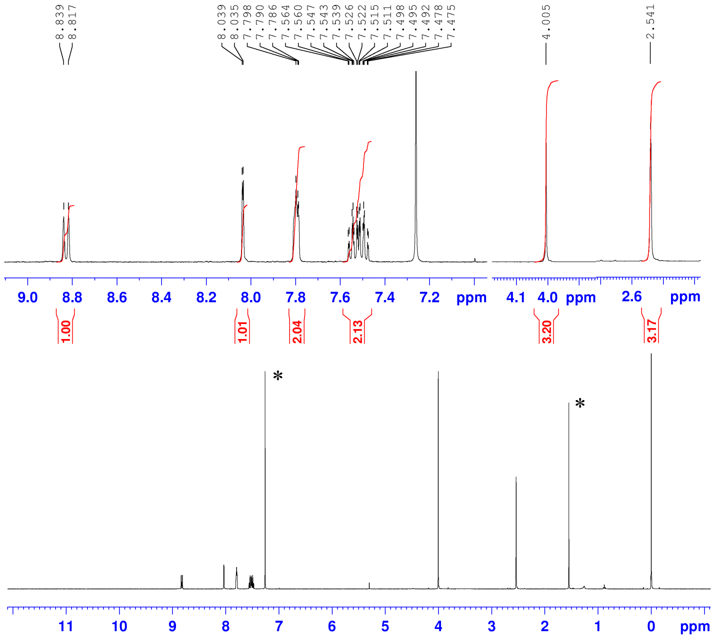

Fig. S12 ^{1}H NMR spectrum of **methyl 3-methyl-1-naphthoate (S18)** in $CDCl_3$ (* solvent peaks).

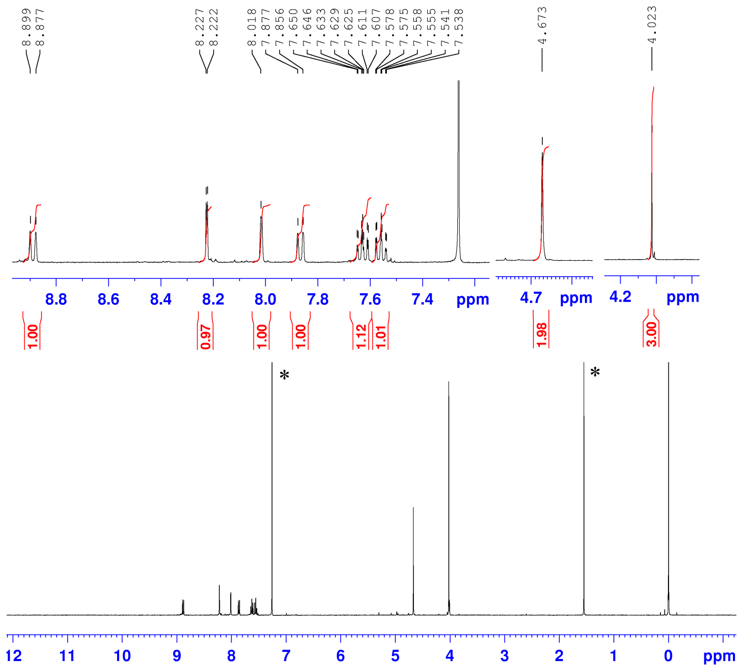

Fig. S13 ^{1}H NMR spectrum of **Methyl 3-(bromomethyl)-1-naphthoate (S19)** in $CDCl_3$ (* solvent peaks).

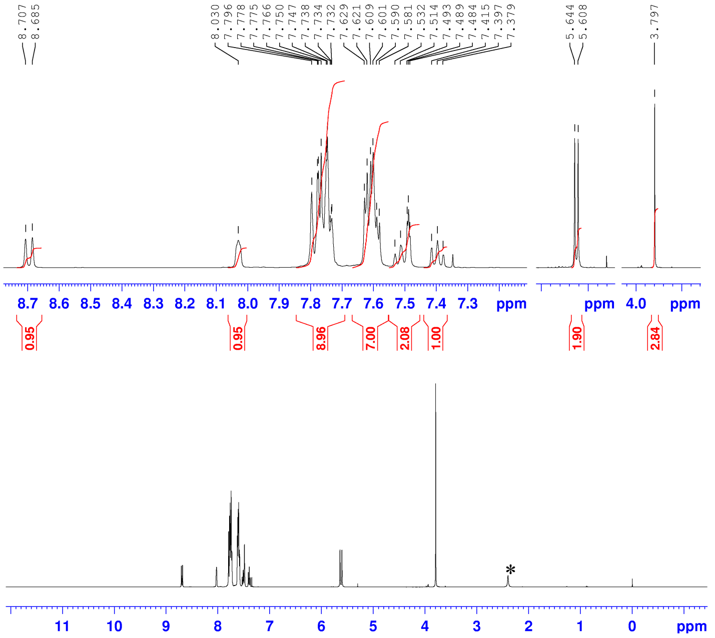

Fig. S14 ^{1}H NMR spectrum of **((4-(methoxycarbonyl)naphthalen-2-yl)methyl)triphenylphosphonium bromide (S20)** in $CDCl_3$ (* solvent peaks).

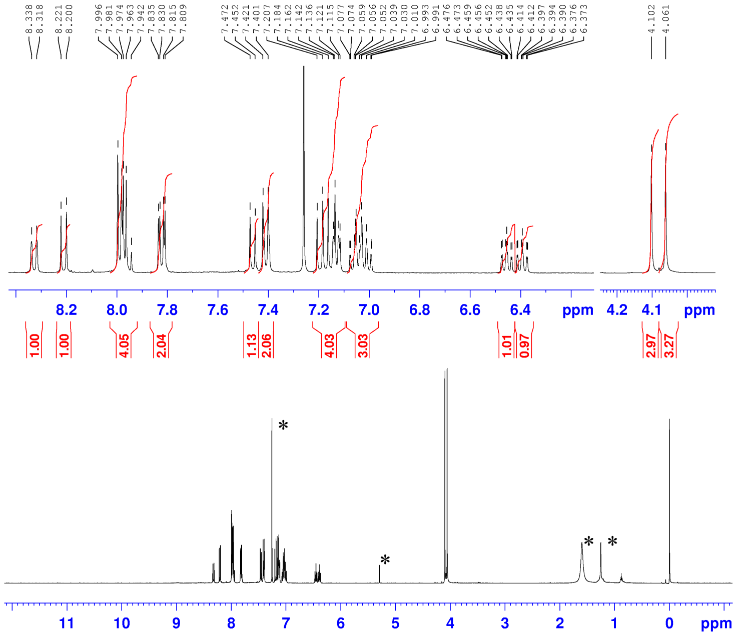

Fig. S15 ^{1}H NMR spectrum of **dimethyl [9]helicene-dicarboxylate (S23)** in $CDCl_3$ (* solvent peaks).

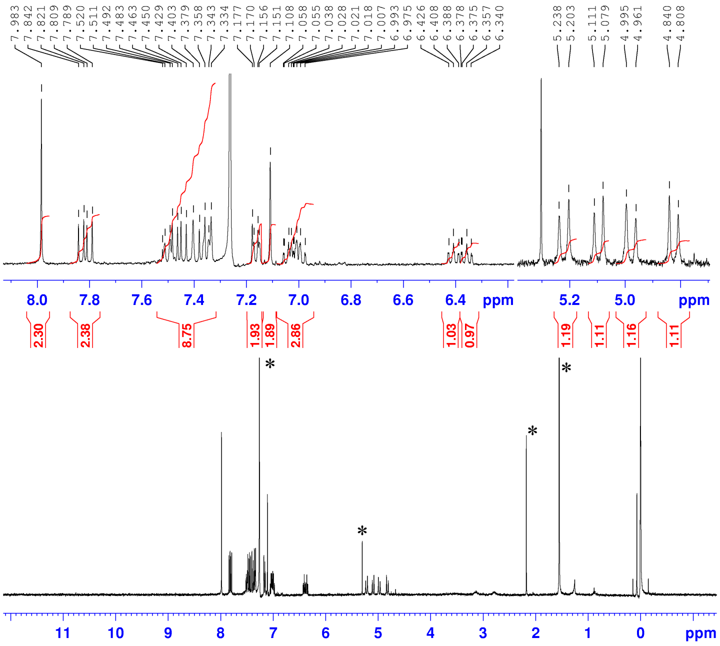

Fig. S16 ^{1}H NMR spectrum of **[9]helicene-dimethanol (S24)** in $CDCl_3$ (* solvent peaks).

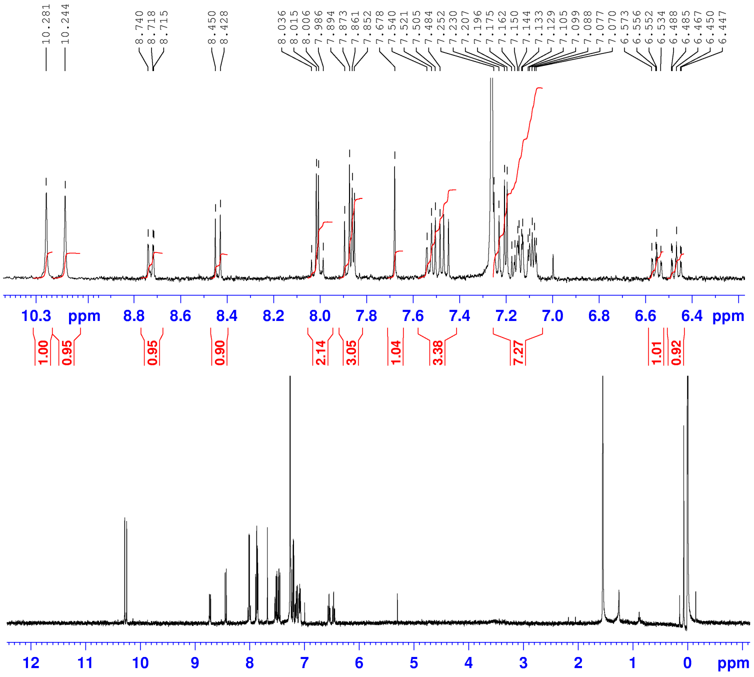

Fig. S17 ^{1}H NMR spectrum of **[9]helicene-dicarbaldehyde (S25)** in $CDCl_3$ (* solvent peaks).

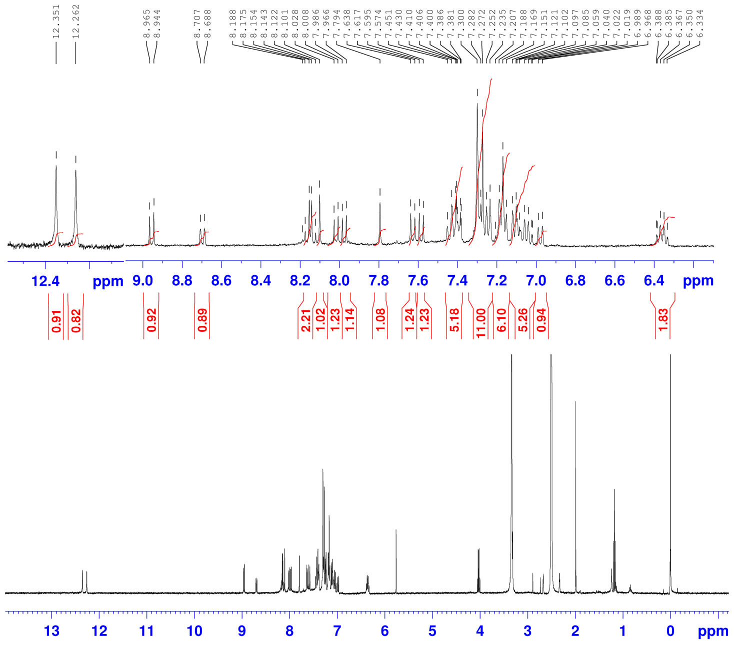

Fig. S18 ^{1}H NMR spectrum of **[9]helicene-ImDL (S26)** in DMSO-d_6 (* solvent peaks).

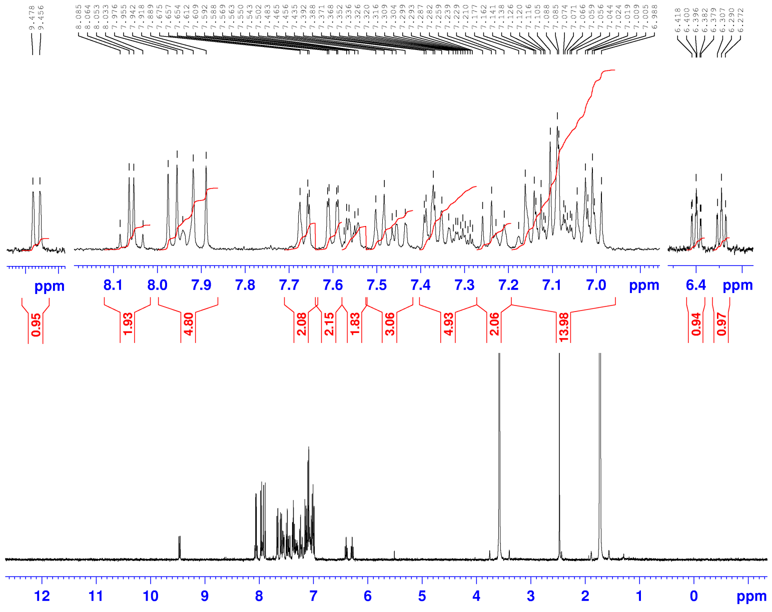

Fig. S19 ^{1}H NMR spectrum of **[9]helicene-ImD** in THF-d_8 (* solvent peaks).

^{13}C NMR Spectra

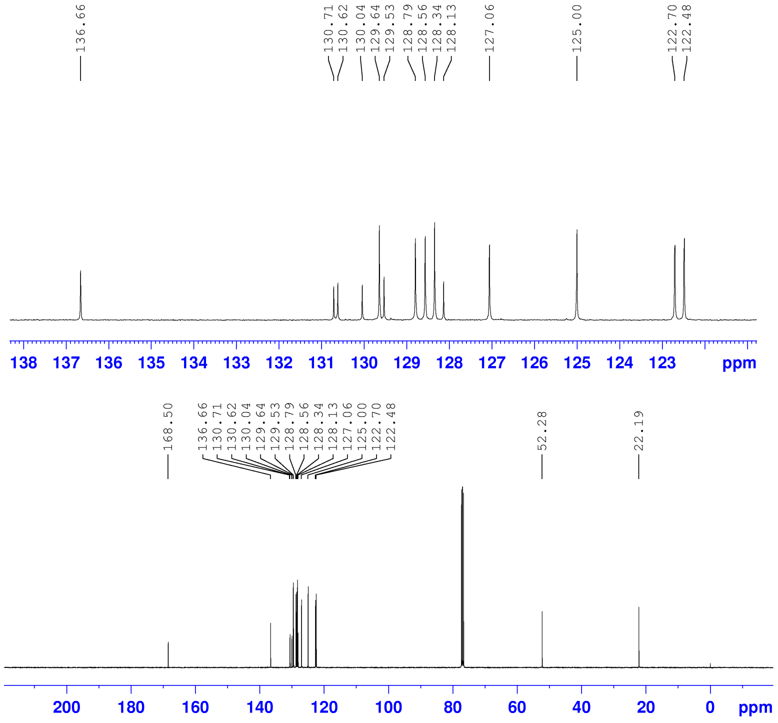

Fig. S20 ^{13}C NMR spectrum of **methyl-6-methylphenanthrene-1-carboxylate (S3)** in $CDCl_3$.

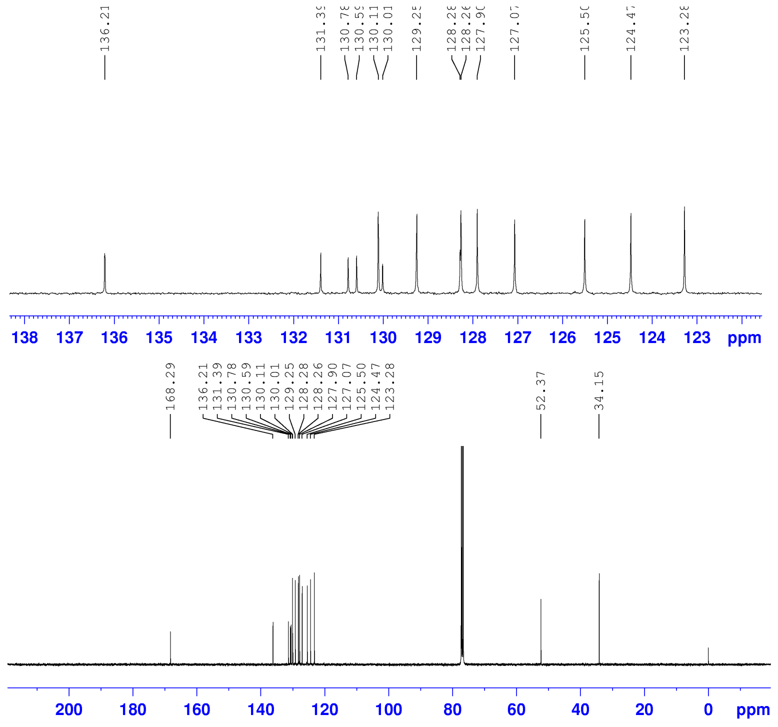

Fig. S21 ^{13}C NMR spectrum of **methyl-6-(bromomethyl)phenanthrene-1-carboxylate (S4)** in $CDCl_3$.

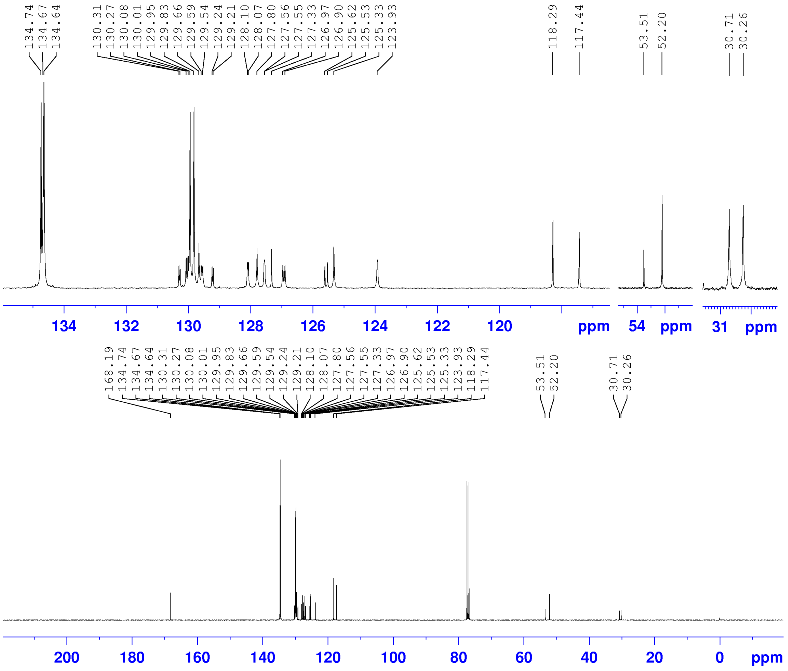

Fig. S22 ^{13}C NMR spectrum of **((8-(methoxycarbonyl)phenanthren-3-yl)methyl)triphenylphosphonium bromide (S5)** in $CDCl_3$.

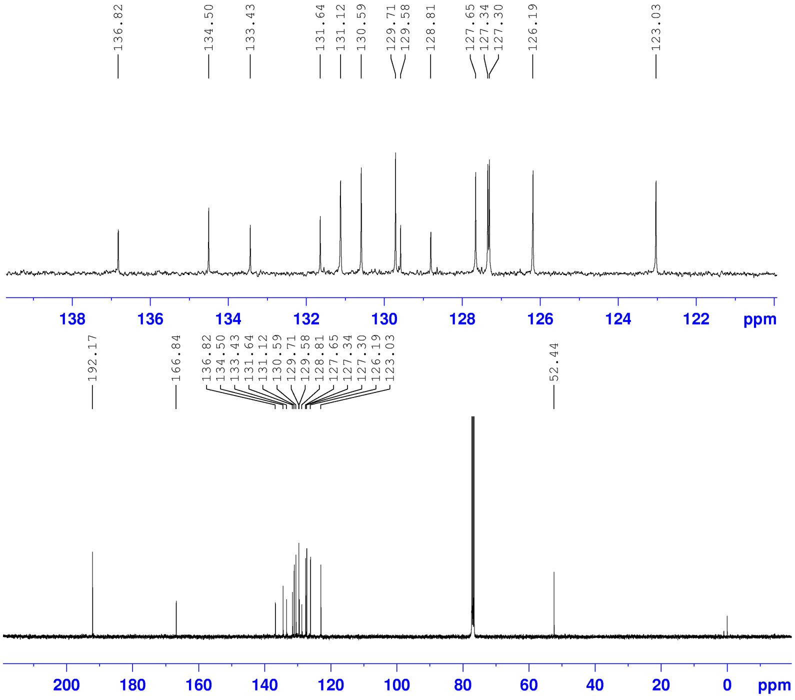

Fig. S23 ^{13}C NMR spectrum of **methyl 6-formylphenanthrene-2-carboxylate (S7)** in $CDCl_3$.

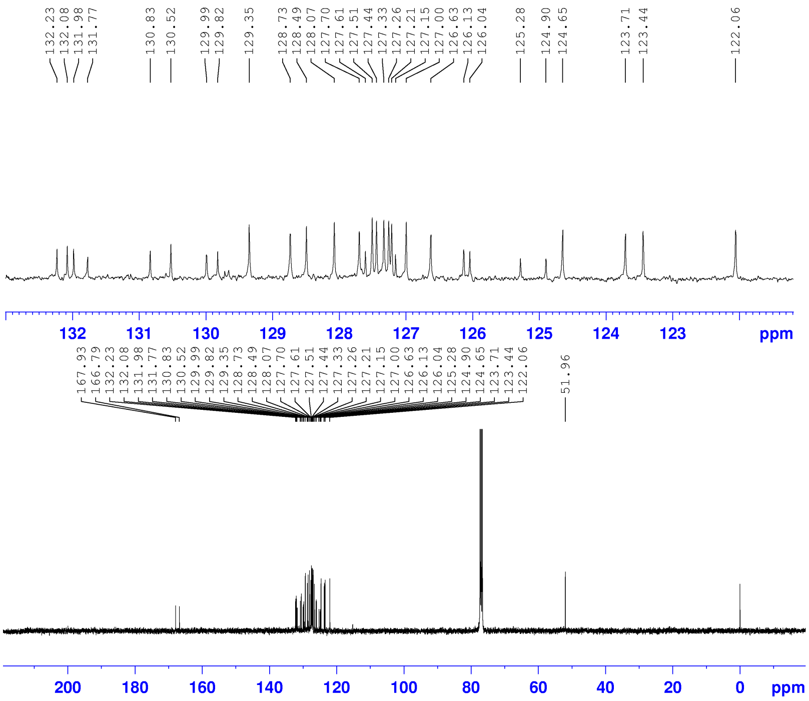

Fig. S24 ^{13}C NMR spectrum of **[7]helicene-di(methylcarboxylate) (S9)** in $CDCl_3$.

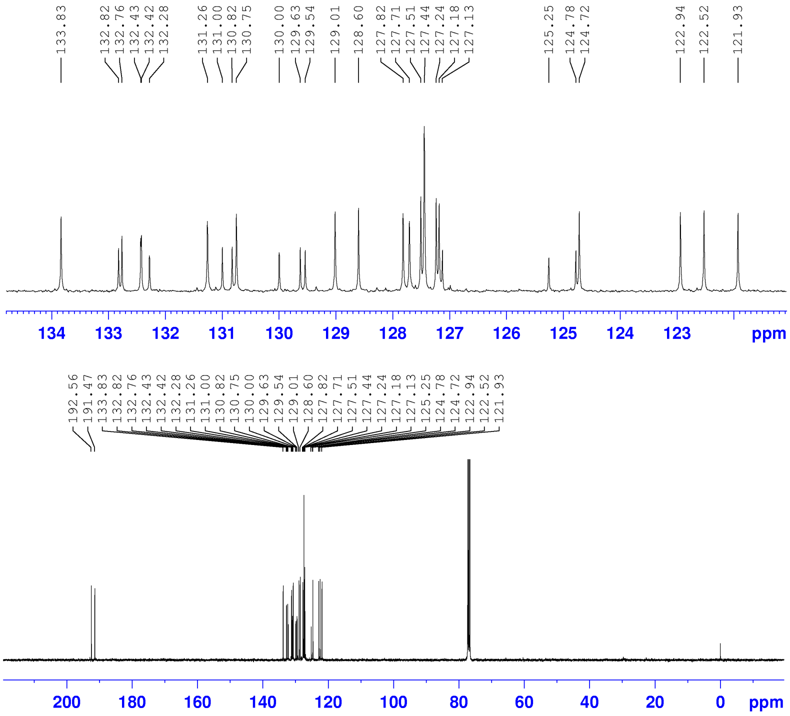

Fig. S25 ^{13}C NMR spectrum of **[7]helicene-dicarbaldehyde (S11)** in $CDCl_3$.

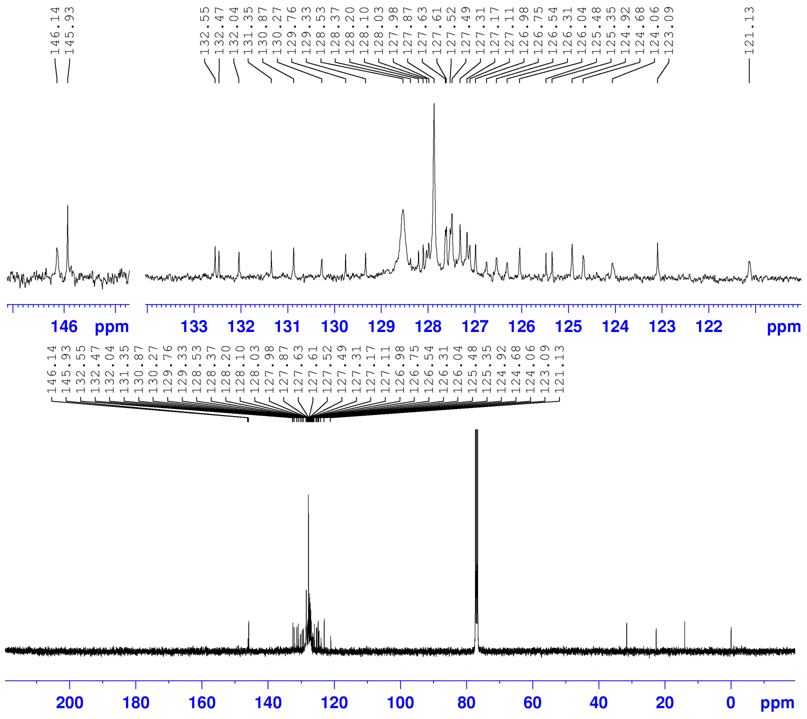

Fig. S26 ^{13}C NMR spectrum of **7H-ImDL (S12)** in DMSO-d_6 at 316 K.

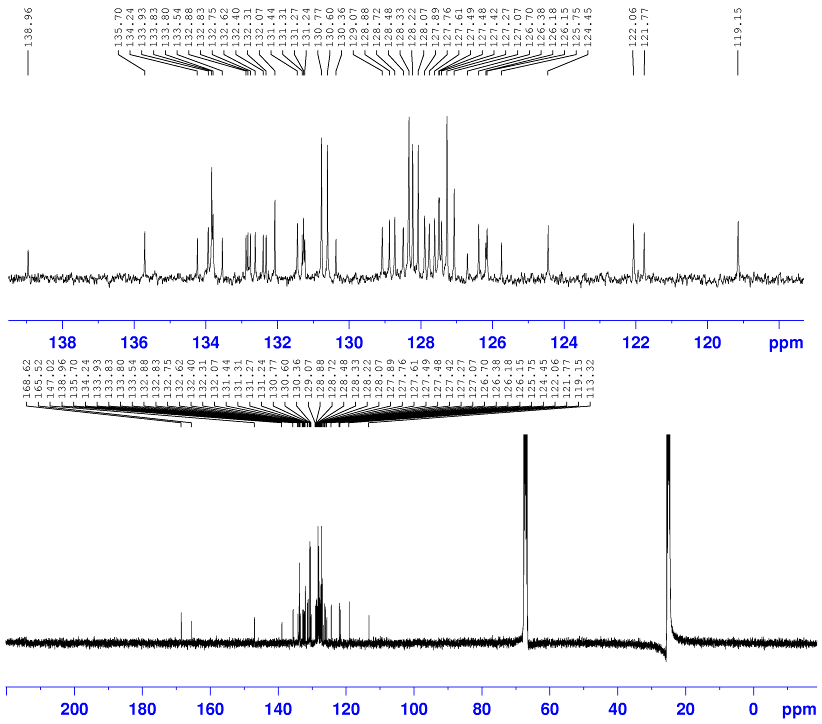

Fig. S27 ^{13}C NMR spectrum of **7H-ImD** in $CDCl_3$.

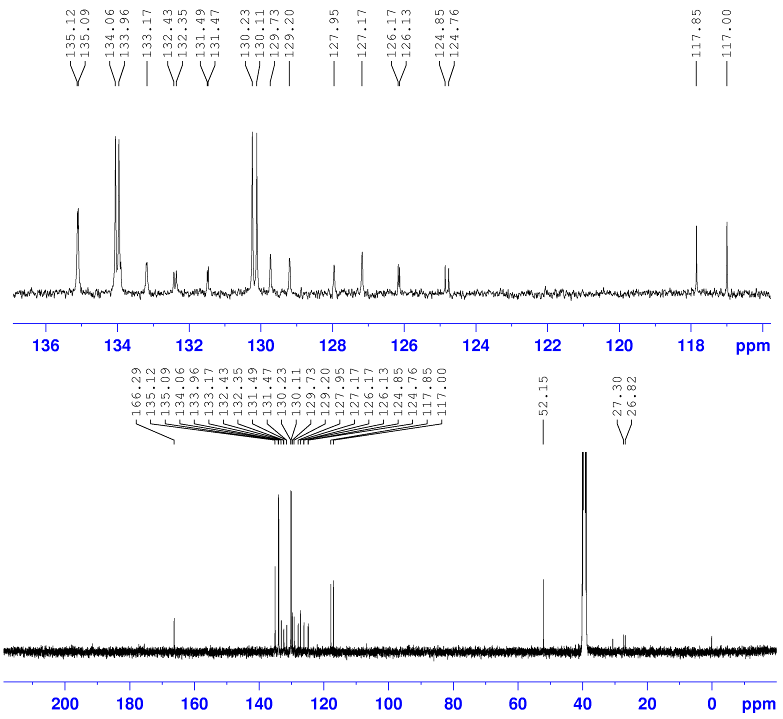

Fig. S28 ^{13}C NMR spectrum of **((3-(methoxycarbonyl)naphthalen-2-yl)methyl)triphenylphosphonium bromide (S14)** in DMSO-d_6.

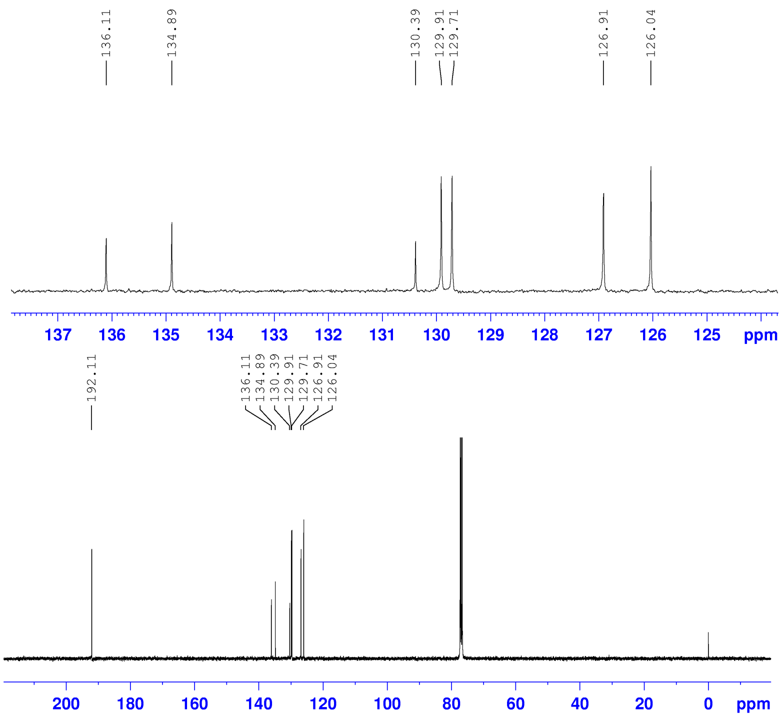

Fig. S29 ^{13}C NMR spectrum of **phenanthrene-3,6-dicarbaldehyde (S16)** in $CDCl_3$.

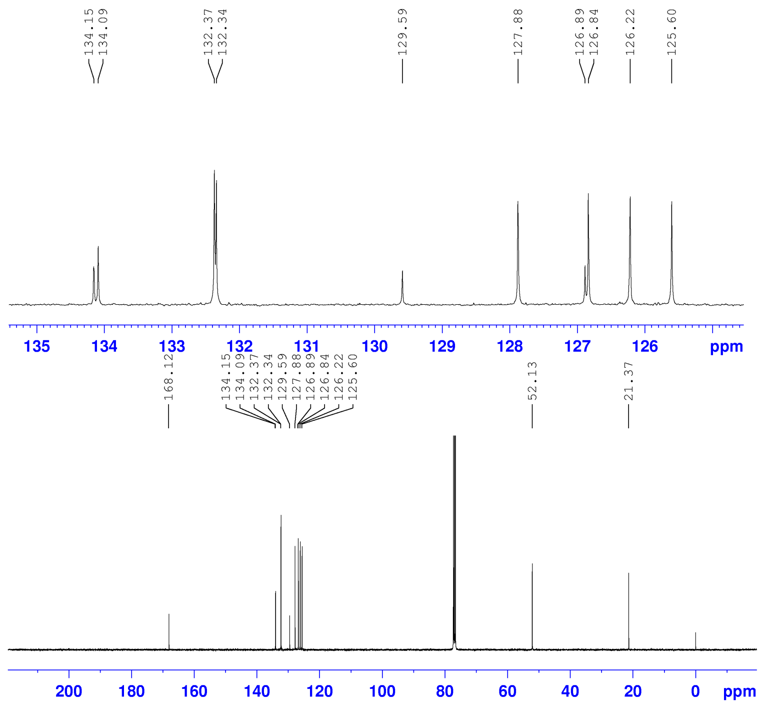

Fig. S30 ^{13}C NMR spectrum of **methyl 3-methyl-1-naphthoate (S18)** in $CDCl_3$.

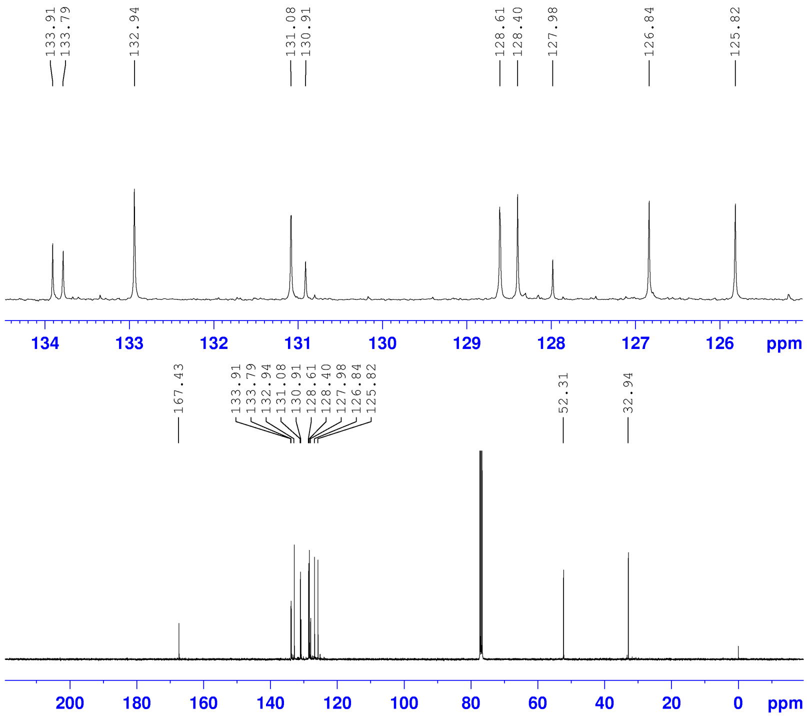

Fig. S31 ^{13}C NMR spectrum of **methyl 3-(bromomethyl)-1-naphthoate (S19)** in $CDCl_3$.

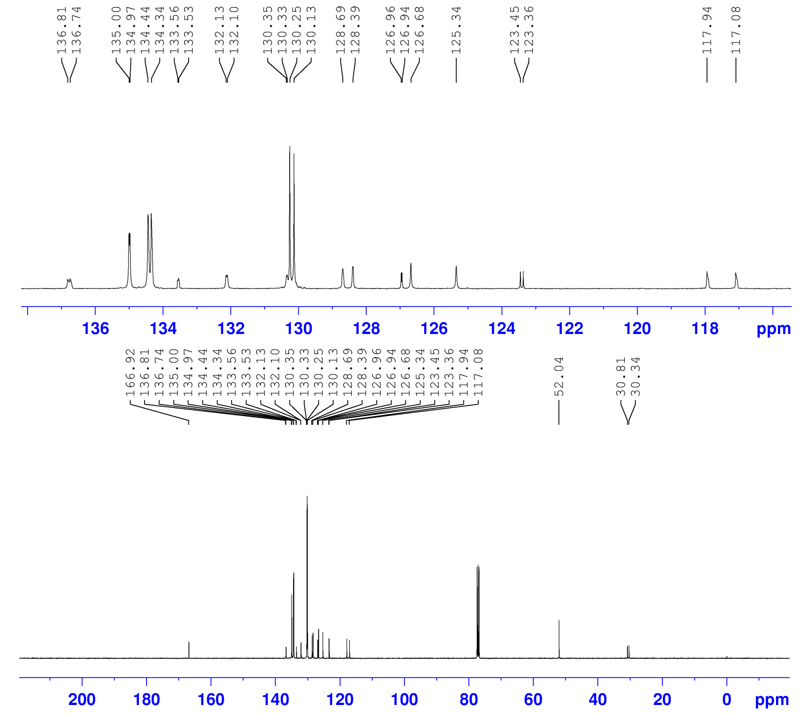

Fig. S32 ^{13}C NMR spectrum of **((4-(methoxycarbonyl)naphthalen-2-yl)methyl)triphenylphosphonium bromide (S20)** in $CDCl_3$.

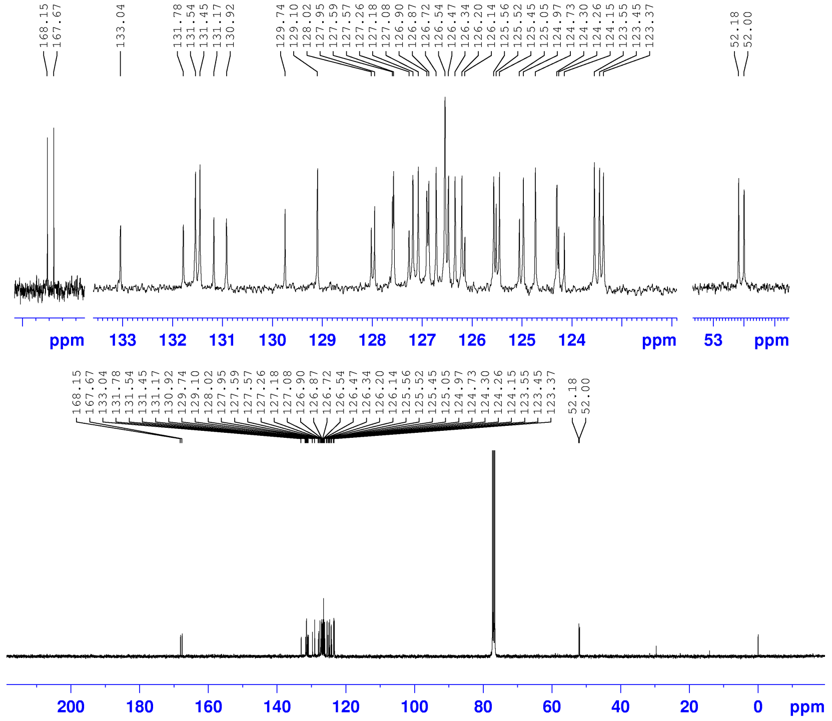

Fig. S33 ^{13}C NMR spectrum of **[9]helicene-dicarboxylate (S23)** in $CDCl_3$.

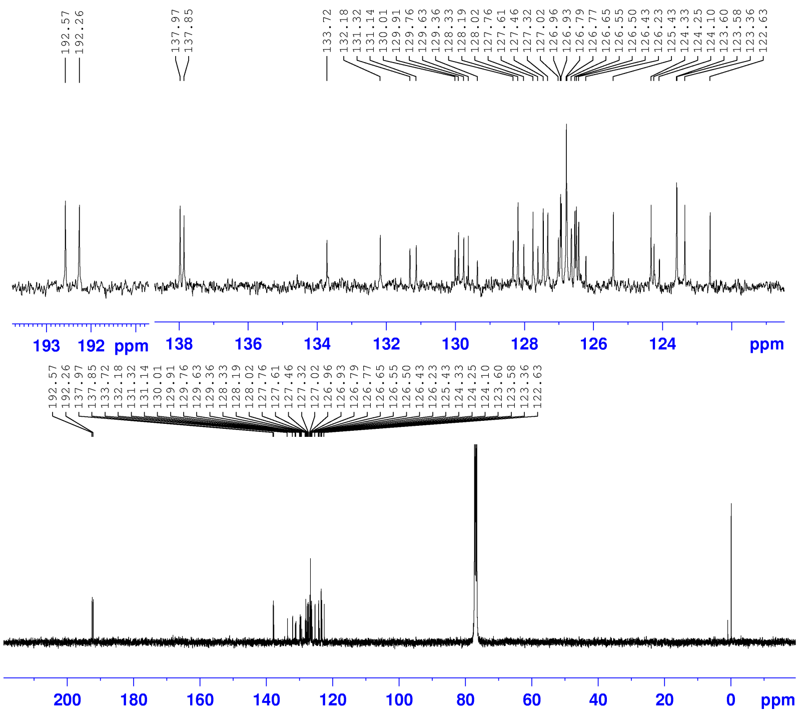

Fig. S34 ^{13}C NMR spectrum of **[9]helicene-dicarbaldehyde (S25)** in $CDCl_3$.

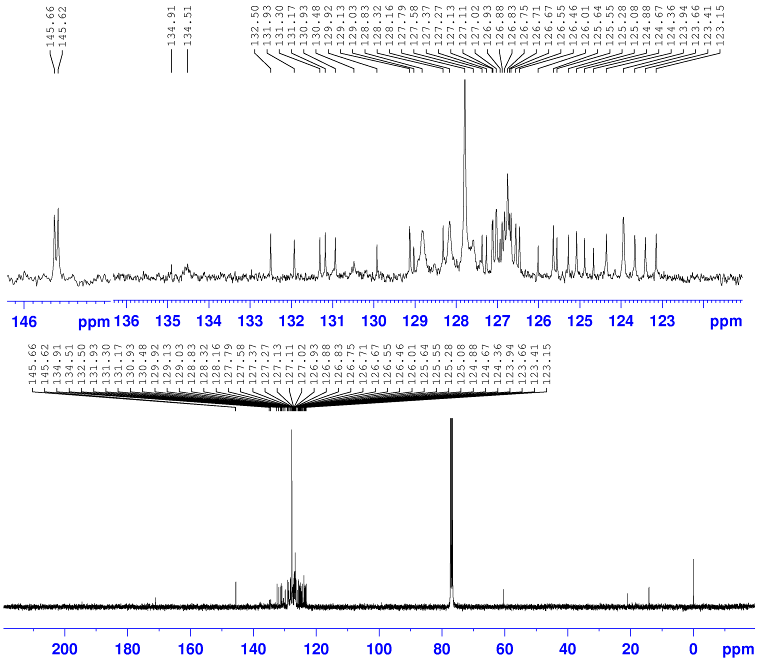

Fig. S35 ^{13}C NMR spectrum of **[9]helicene-ImDL (S26)** in $CDCl_3$.

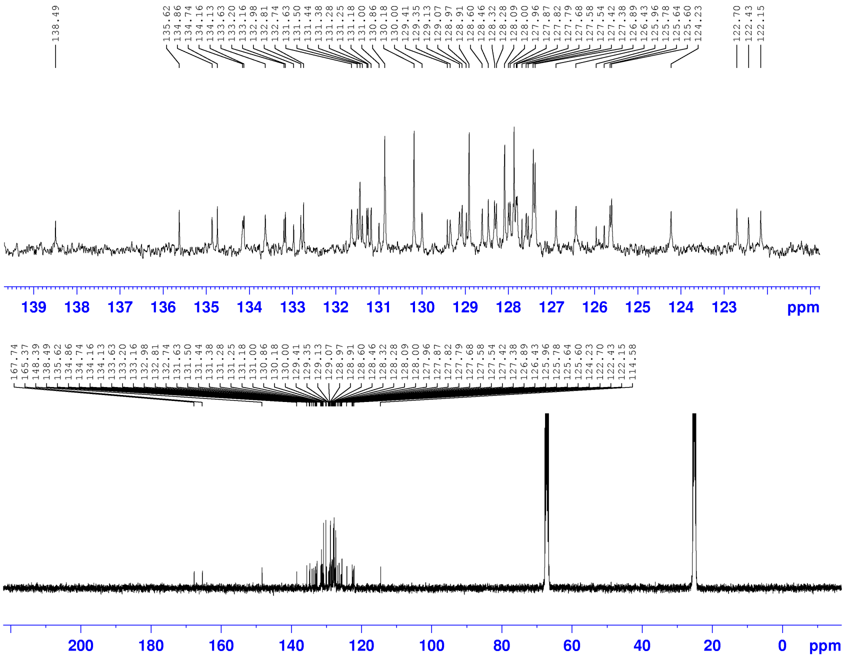

Fig. S36 ^{13}C NMR spectrum of **[9]helicene-ImD** in THF-d_8.

3. HR-ESI-TOF MS Spectra

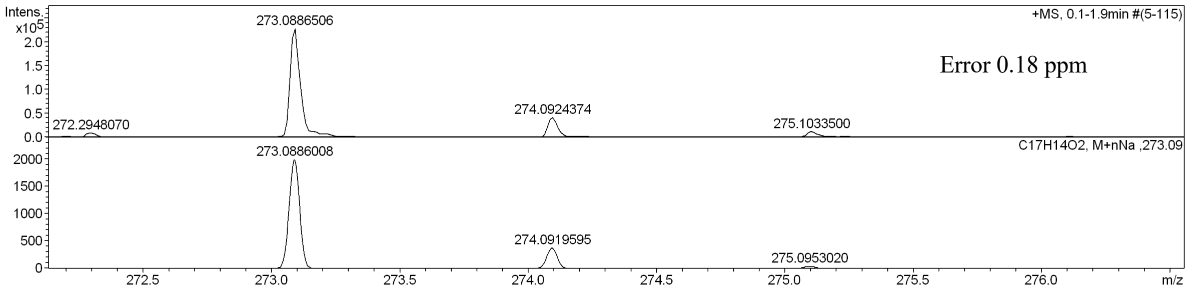

Fig. S37 HR-ESI-TOF MS spectra of **methyl-6-methylphenanthrene-1-carboxylate (S3)**.

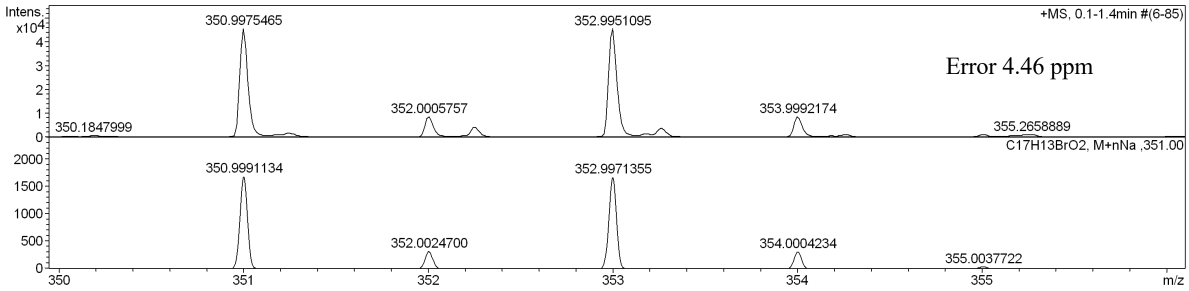

Fig. S38 HR-ESI-TOF MS spectra of **methyl-6-(bromomethyl)phenanthrene-1-carboxylate (S4)**.

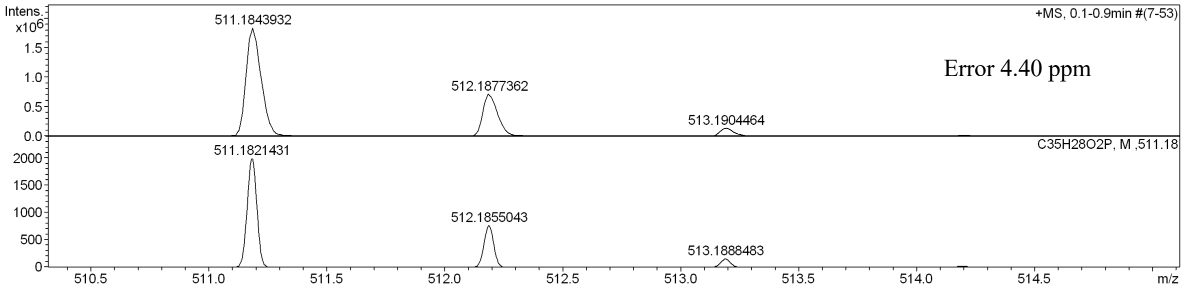

Fig. S40 HR-ESI-TOF MS spectra of **((8-(methoxycarbonyl)phenanthren-3-yl)methyl)triphenylphosphonium bromide (S5)**.

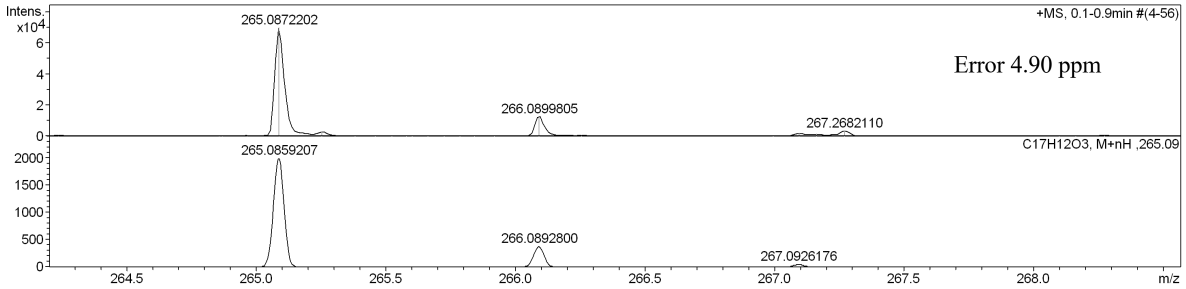

Fig. S41 HR-ESI-TOF MS spectra of **methyl 6-formylphenanthrene-2-carboxylate (S7)**.

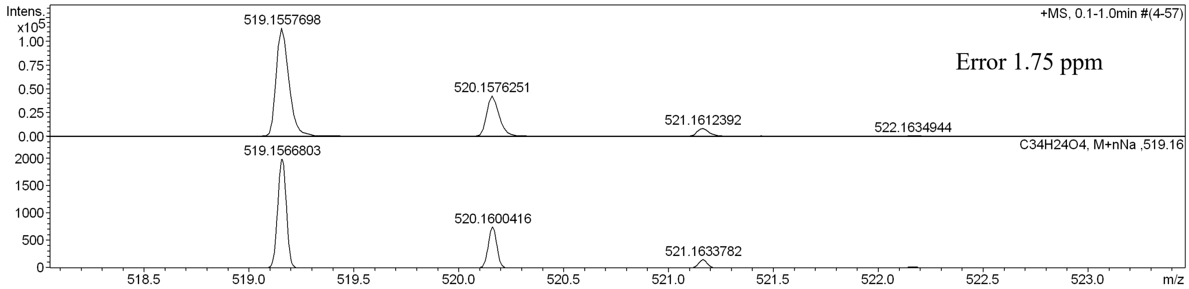

Fig. S42 HR-ESI-TOF MS spectra of **methyl 6-(2-(7-(methoxycarbonyl)phenanthren-3-yl)vinyl)phenanthrene-1-carboxylate (S8)**.

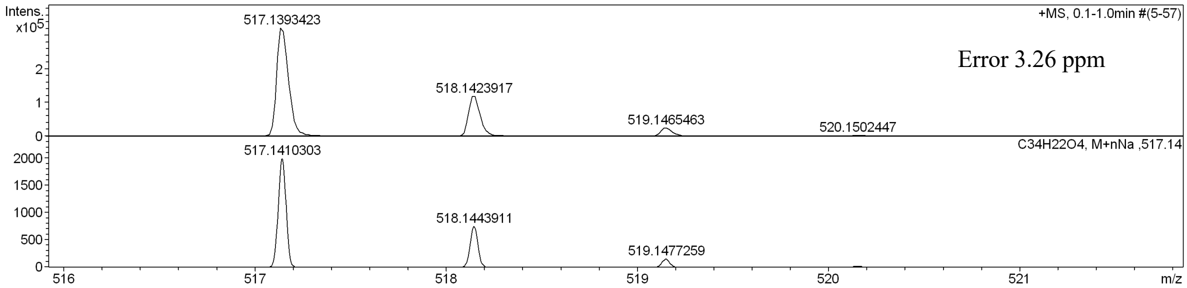

Fig. S43 HR-ESI-TOF MS spectra of **[7]helicene-di(methylcarboxylate) (S9)**.

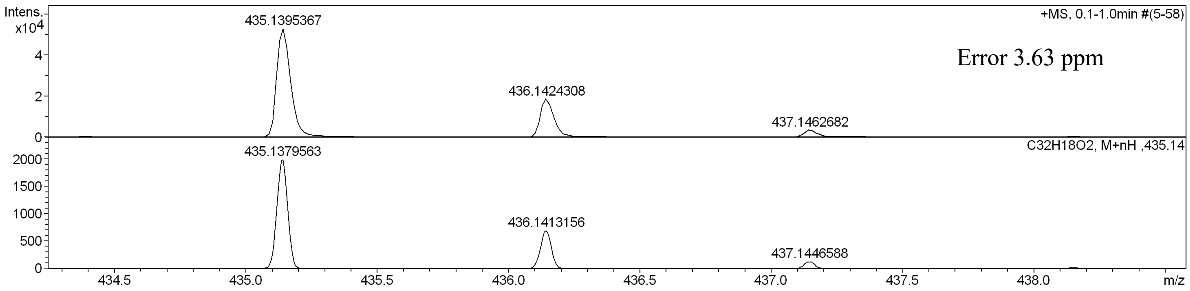

Fig. S44 HR-ESI-TOF MS spectra of **[7]helicene-dicarbaldehyde (S11)**.

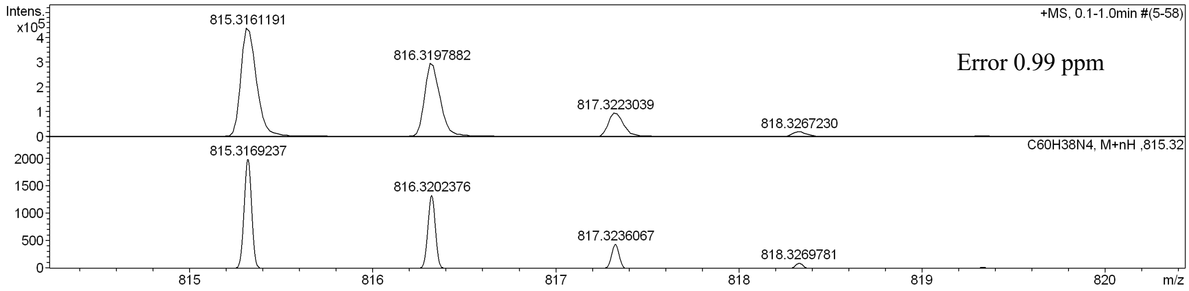

Fig. S45 HR-ESI-TOF MS spectra of **7H-ImDL (S12)**.

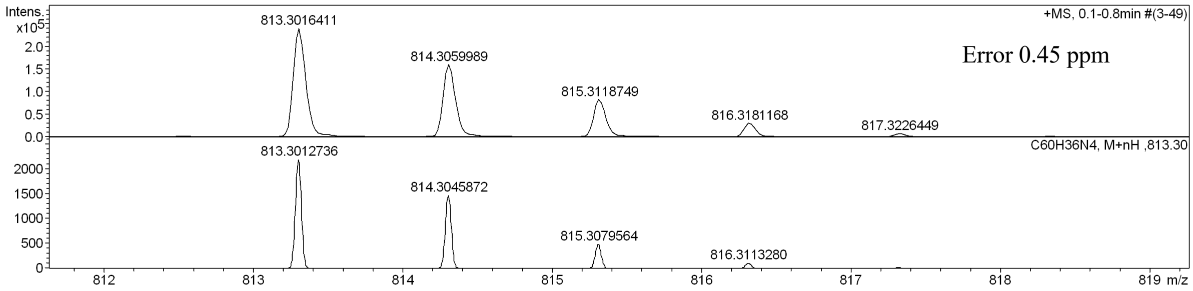

Fig. S46 HR-ESI-TOF MS spectra of **7H-ImD**.

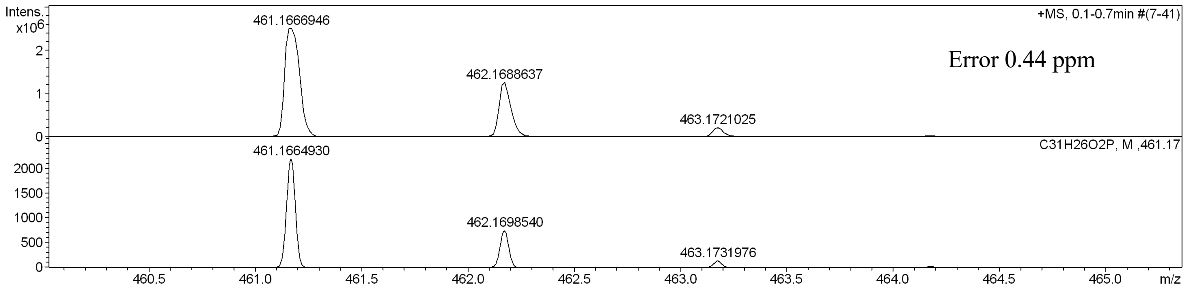

Fig. S47 HR-ESI-TOF MS spectra of **((3-(methoxycarbonyl)naphthalen-2-yl)methyl)triphenylphosphonium bromide (S14)**.

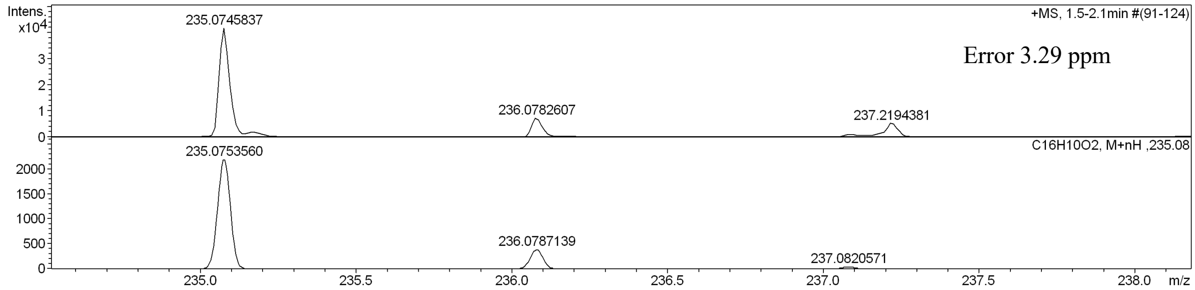

Fig. S48 HR-ESI-TOF MS spectra of **phenanthrene-3,6-dicarbaldehyde (S16)**.

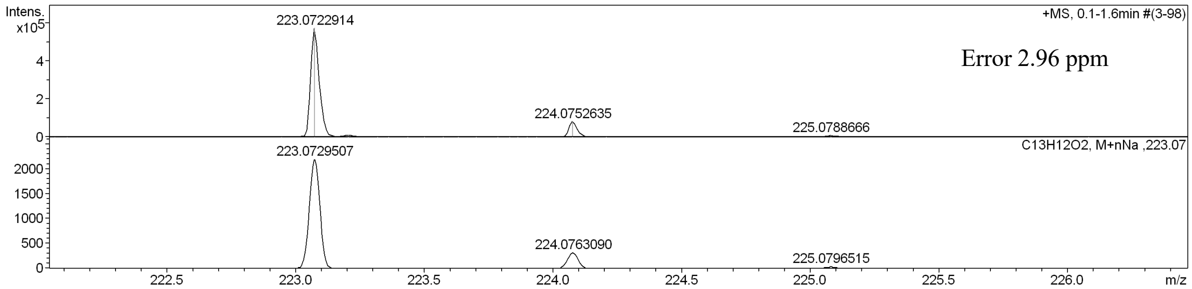

Fig. S49 HR-ESI-TOF MS spectra of **methyl 3-methyl-1-naphthoate (S18)**.

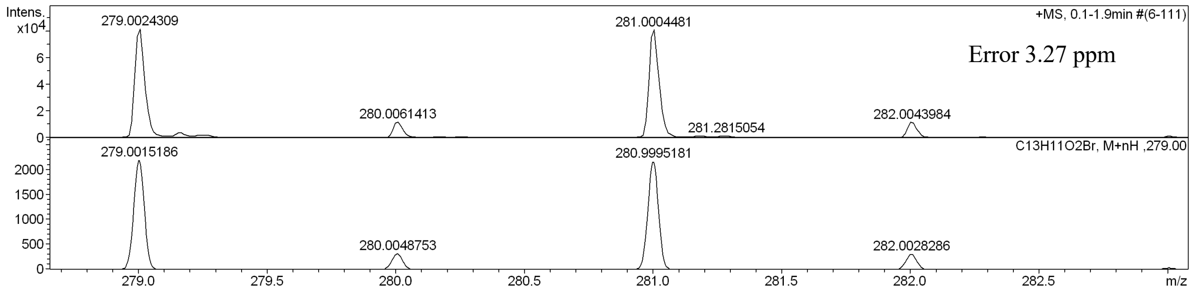

Fig. S50 HR-ESI-TOF MS spectra of **methyl 3-(bromomethyl)-1-naphthoate (S19)**.

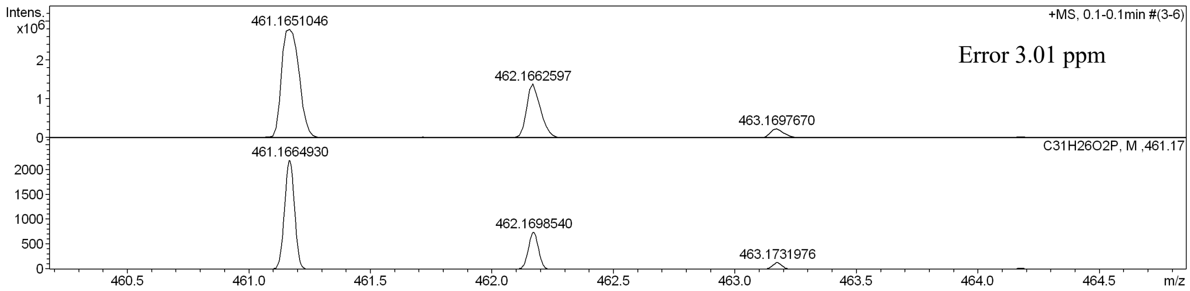

Fig. S51 HR-ESI-TOF MS spectra of **((4-(methoxycarbonyl)naphthalen-2-yl)methyl)triphenylphosphonium bromide (S20)**.

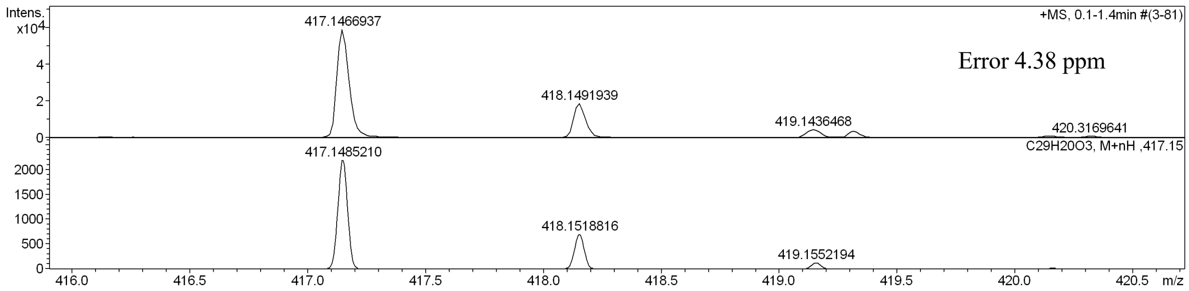

Fig. S52 HR-ESI-TOF MS spectra of **methyl 3-(2-(6-formylphenanthren-3-yl)vinyl)-2-naphthoate (S21)**.

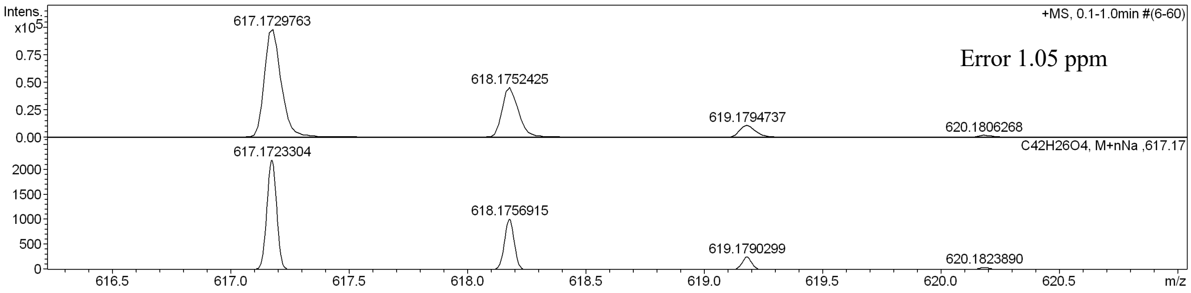

Fig. S53 HR-ESI-TOF MS spectra of **dimethyl [9]helicene-dicarboxylate (S23)**.

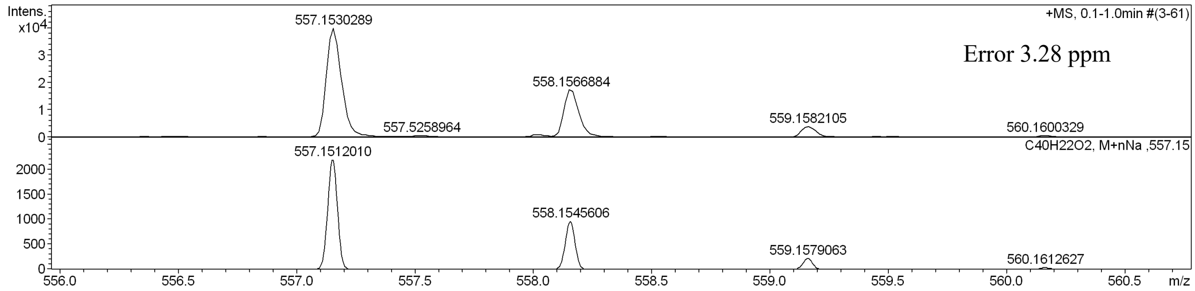

Fig. S54 HR-ESI-TOF MS spectra of **[9]helicene-dicarbaldehyde (S25)**.

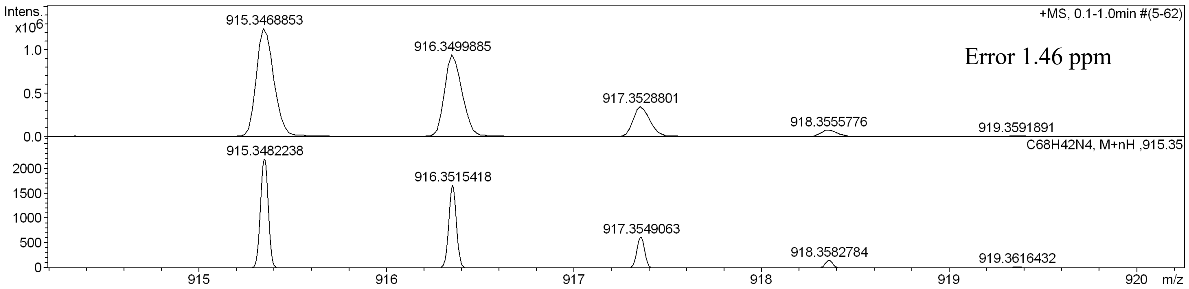

Fig. S55 HR-ESI-TOF MS spectra of **[9]helicene-ImDL (S26)**.

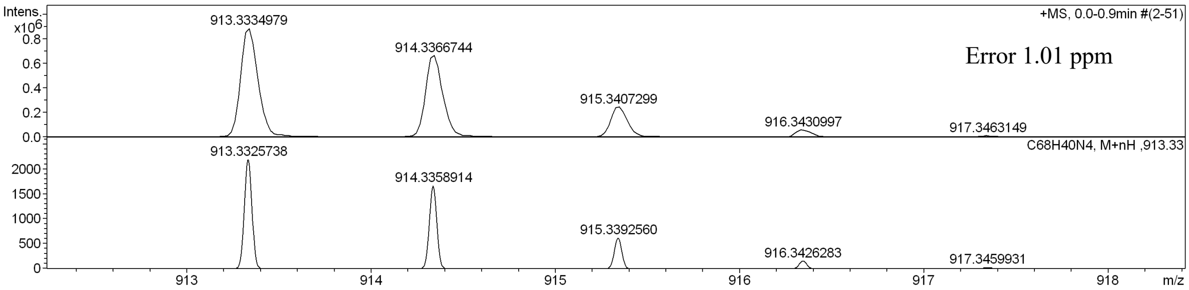

Fig. S56 HR-ESI-TOF MS spectra of **[9]helicene-ImD**.

4. HPLC Chromatograms

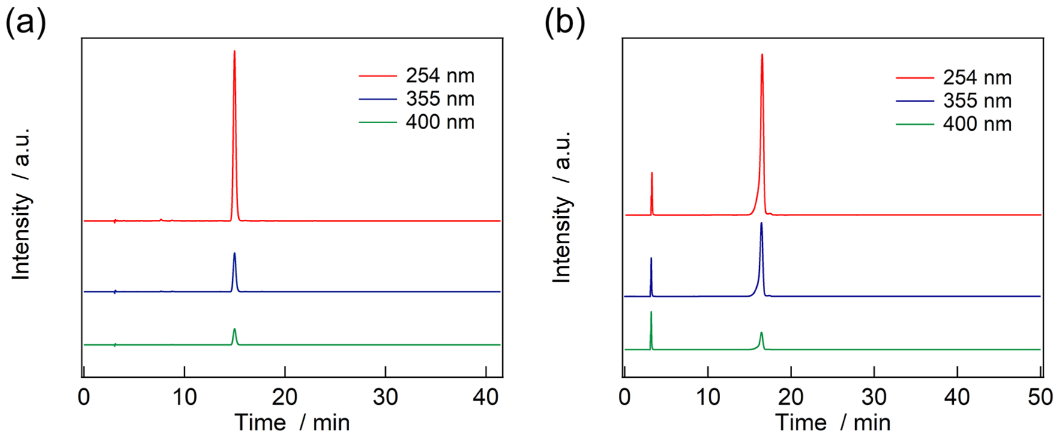

Fig. S57 HPLC chromatograms of (a) **7H-ImD**; 99 % purity, and (b) **9H-ImD**; 98 % purity. HPLC analysis was performed using a reverse phase analytical column (Mightysil RP18, 25 cm×4.6 mm, 5 μm particle) from Kanto Chemical Industries, equipped with a PDA detector; the mobile phase was CH_3CN with a flow rate of 1.0 mL/min (detection wavelength; 254, 355, and 400 nm). (* solvent peaks)

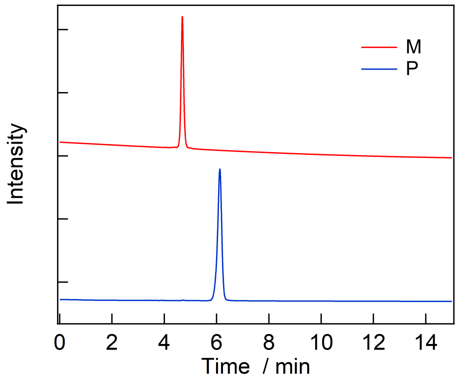

Fig. S58 Chiral HPLC chromatograms of **[9]helicene-dicarbaldehyde (S25)**. HPLC analysis was performed using a DAICEL, CHIRALPAK IC, equipped with a PDA detector; the mobile phase was CH_2Cl_2/hexane/THF = 1/1/2 with a flow rate of 1.0 mL/min (detection wavelength; 254 nm).

5. CD Spectroscopy

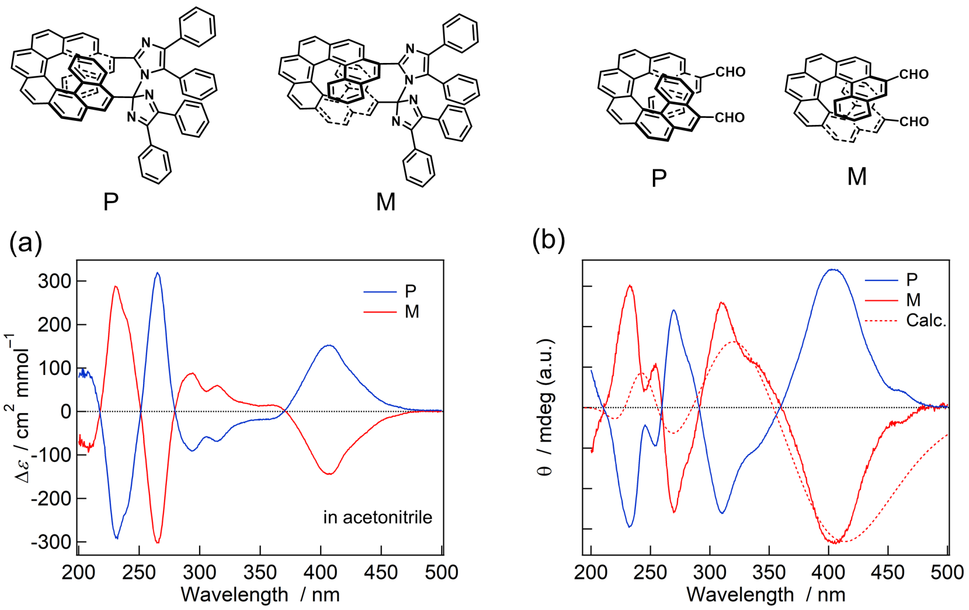

Fig. S59 CD spectra (JASCO J-820) of (a) **(M)-** and **(P)-9H-ImD** and (b) **(M)-** and **(P)-[9]helicene-dicarbaldehyde (S25)** in acetonitrile at 298 K. The TDDFT calculation was performed for **(M)-[9]helicene-dicarbaldehyde** (MPW1PW91/6-31+G(d,p)//MPW1PW91/6-31G(d) level of the theory) to determine the absolute configuration.

6. Transient Absorption Spectroscopy

The UV light (365 nm, 100 mW) was irradiated to the benzene solution of 7H-ImD in a quartz optical cuvette (optical length = 1 cm) using LED light source (CL-H1-470-9-1, Asahi Spectra Co., Ltd.). The transient absorption spectra and the time variation of the transient absorbance were recorded on an Ocean FX multichannel detector (Ocean Optics, Inc). The power of the excitation light was measured using NOVA (OPHIR Optronics Solutions Ltd.) equipped with a power thermal sensor 30A-P17 (OPHIR Optronics Solutions Ltd.). CUV-QPOD (Ocean Optics, Inc) equipped with a TC 125 temperature controller (QUANTUM) was used as a cuvette holder. A deuterium and a halogen lamps DH-2000-BAL (Ocean Optics, Inc) were used as the probe beam, which were guided with a QP-600-1-SR optical fiber (Ocean Optics, Inc). Optical grade solvents were used for all measurements.

The laser flash photolysis experiments were performed for 9H-ImD by a TSP-2000 time-resolved spectrophotometer (Unisoku). A 10 Hz Qswitched Nd:YAG laser (Continuum Minilite II) with the third harmonic at 355 nm (pulse width = 5 ns) was employed as the excitation light source. A halogen lamp (OSRAM HLX 64623) was used as the probe beam arranged in an orientation perpendicular to the exciting laser beam. The probe beam was monitored with a photomultiplier tube (Hamamatsu R2949) through a spectrometer (Unisoku MD200). The excitation intensity was estimated by an energy detector (Gentec Electro-Optics QE12LP-S-MB) with an energy monitor (Genetic Electro-Optics MAESTRO). Optical grade solvents were used for all measurements.

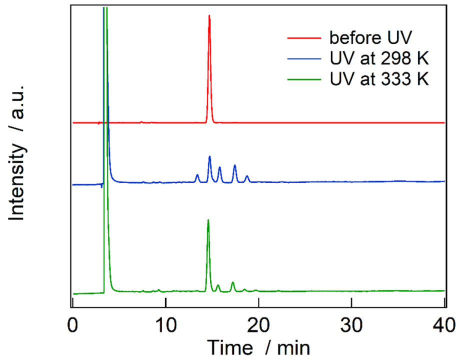

Fig. S60 The HPLC analysis for the benzene solution of **7H-ImD** (red) before UV light irradiation, (blue, green) after UV light irradiation and the subsequent thermal back reaction (blue) at 298 K, and (green) at 333 K.

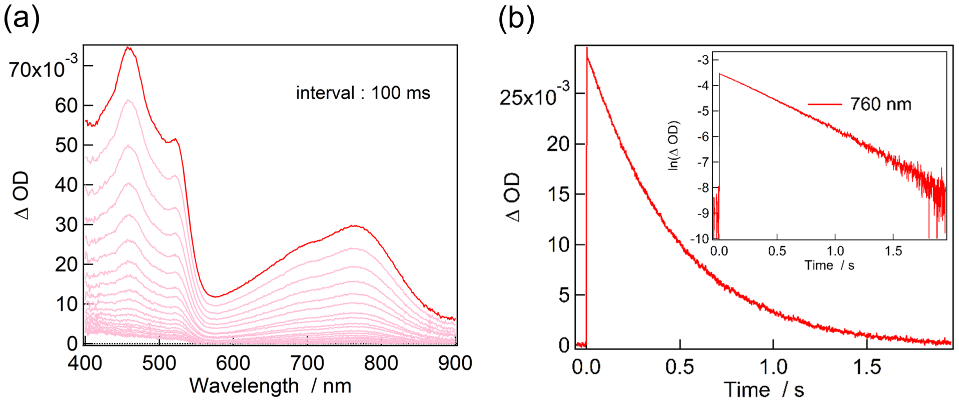

Fig. S61 (a) The transient absorption spectra of 7H-ImD upon 355-nm nanosecond laser irradiation (pulse width = 5 ns, energy = 3 mJ) in benzene at 333 K. (b) The time profiles of the transient absorbance of 7H-ImD ($\lambda_{obs.}$ = 760 nm, $\lambda_{ex.}$ = 355 nm, 3 mJ) in benzene at 333 K. Inset shows the first-order plots for the transient absorbance.

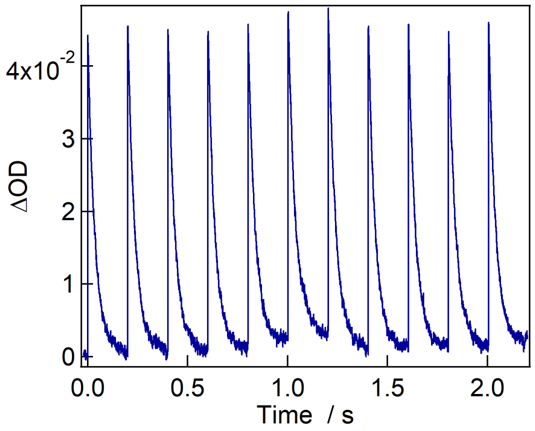

Fig. S62 The reversibility of the photochromic reaction of 9H-ImD (λ_{obs} = 500 nm, λ_{ex} = 355 nm, 3 mJ) at 303 K.

7. Eyring Analysis

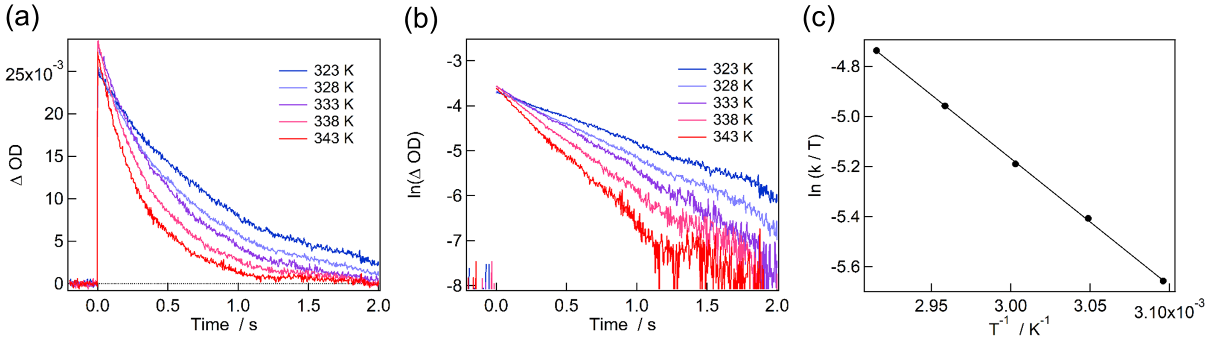

Fig. S63 (a) Time profiles of the transient absorbance of **7H-ImD** monitored at 760 nm in degassed benzene. The measurements were performed in the temperature range from 323 to 343 K. (b) The first order plots of the time profiles. (c) Eyring plots for the thermal back reaction of the biradical species of **7H-ImD**.

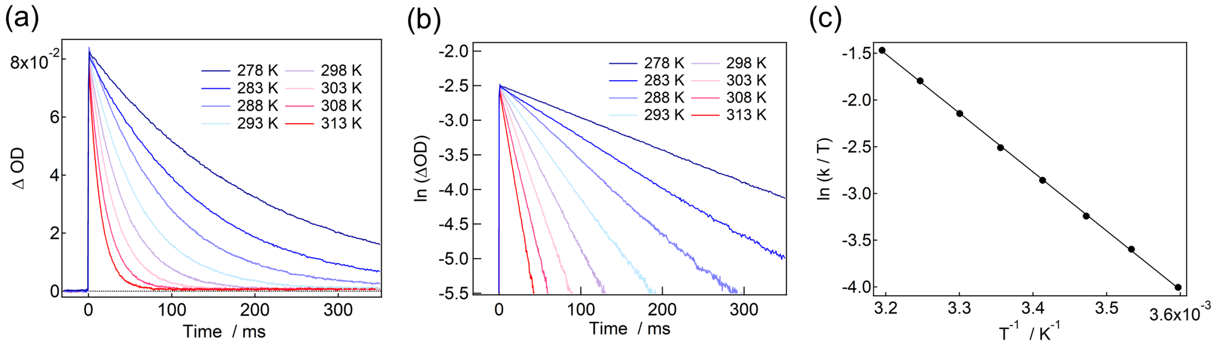

Fig. S64 (a) Time profiles of the transient absorbance of **9H-ImD** monitored at 500 nm in degassed benzene. The measurements were performed in the temperature range from 278 to 313 K. (b) The first order plots of the time profiles. (c) Eyring plots for the thermal back reaction of the biradical species of **9H-ImD**.

8. DFT Calculation

All calculations were carried out using the Gaussian 09 program (Revision D.01).[S6] The molecular structures were fully optimized at the (U)M05-2X/6-31G(d) level of the theory. The analytical second derivative was computed using vibrational analysis to confirm each stationary point to be a minimum. The TDDFT calculations were performed at the MPW1PW91/6-31+G(d,p) level of the theory for the optimized structures.

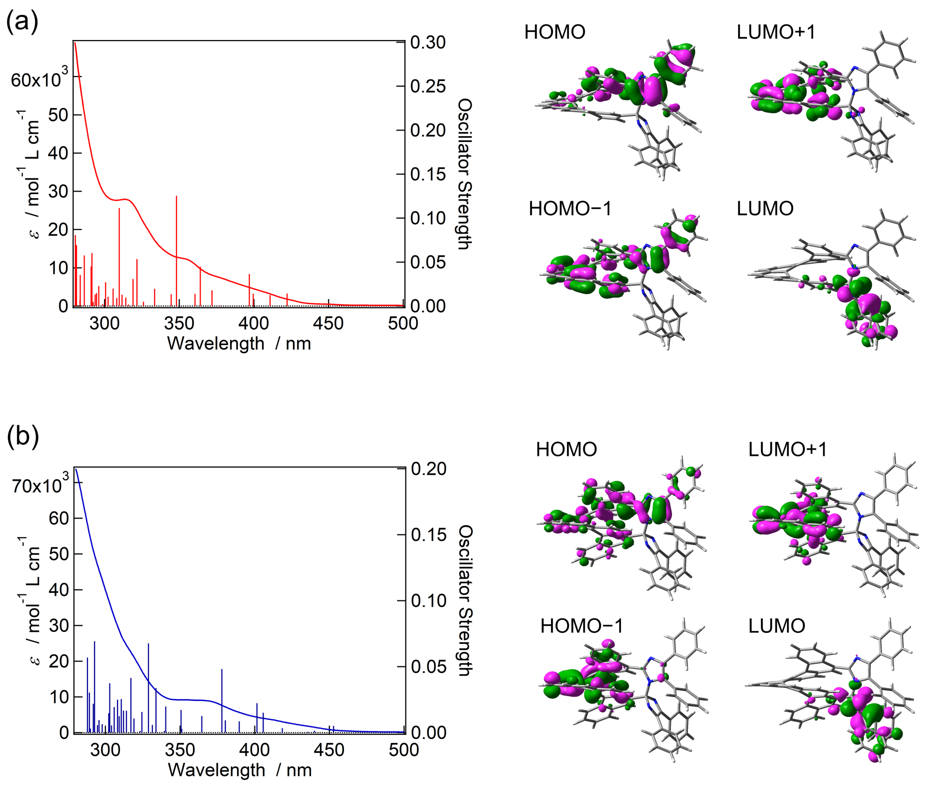

Fig. S65 The absorption spectra in benzene and the TDDFT calculation results (MPW1PW91/6-31+G(d,p)//M05-2X/6-31G(d) level of the theory) for (a) 7H-ImD and (b) 9H-ImD.

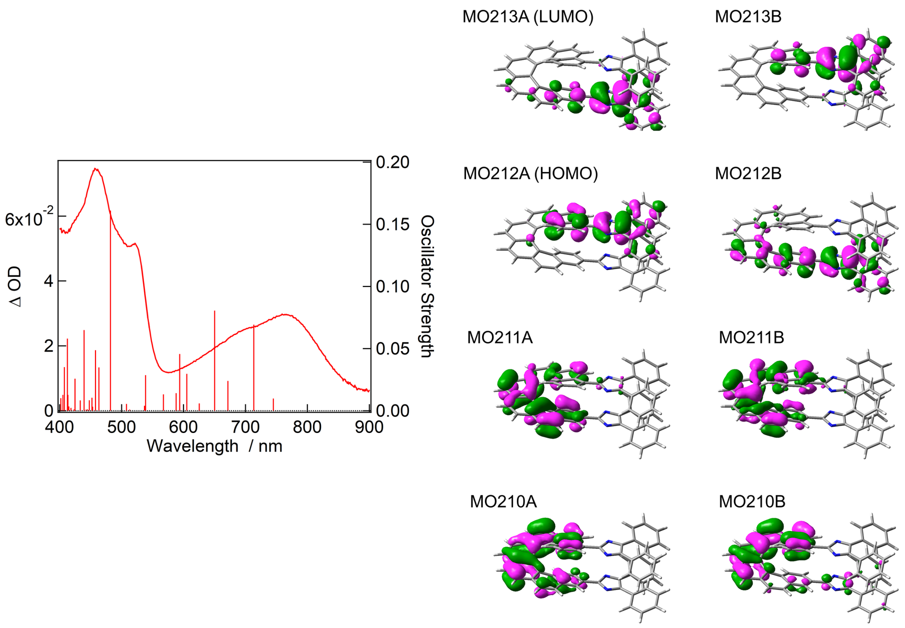

Fig. S66 The transient absorption spectrum in benzene and the TDDFT calculation results (UMPW1PW91/6-31+G(d,p)//UM05-2X/6-31G(d) level of the theory) for 7H-BR.

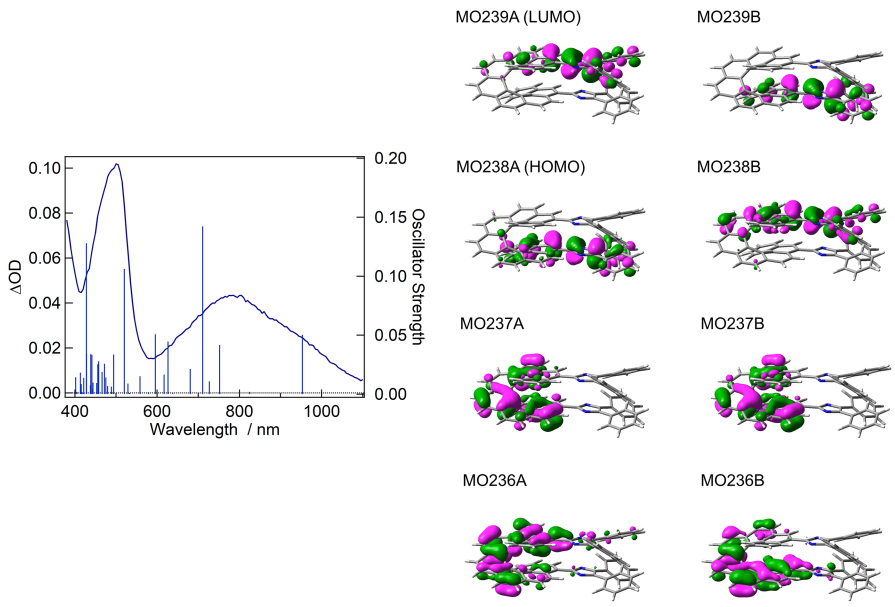

Fig. S67 The transient absorption spectrum in benzene and the TDDFT calculation results (UMPW1PW91/6-31+G(d,p)//UM05-2X/6-31G(d) level of the theory) for 9H-BR.

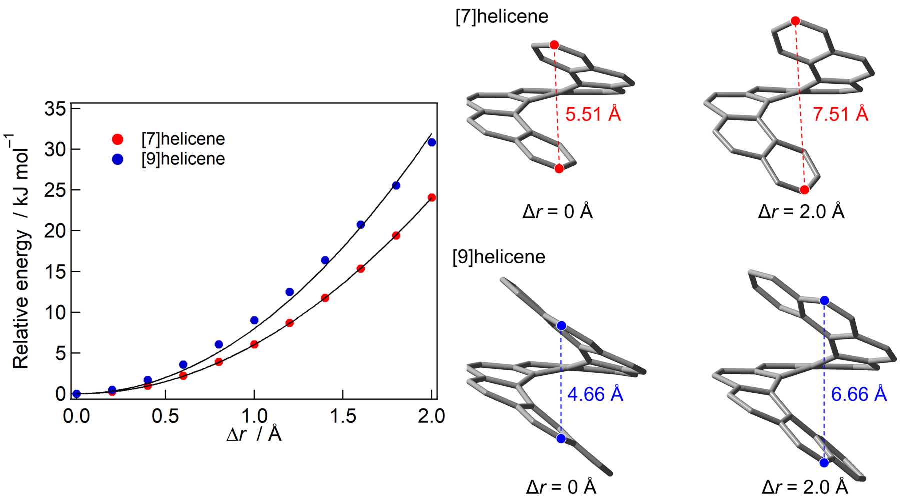

Fig. S68 Increase in the energy upon elongation of the pitch length of [7]helicene and [9]helicene estimated by the TDDFT calculation (B3LYP/6-311G(2d,p) level of the theory). The distance between the two carbon atoms for the most stable optimized structures was estimated to be 5.55 and 4.66 Å for [7]helicene and [9]helicene, respectively. A relaxed potential energy surface was scanned with 10 steps in 0.2 Å increments. The molecular geometry was optimized in each step with a fixed distance between the two carbon atoms (r). Black solid lines show the fitting results of the energy plots by $\Delta E = \frac{1}{2} \cdot A \cdot (\Delta r^2)$, where E is relative energy, and r is the elongation of the distance between the two carbon atoms. The force constant k ($\mathrm{N \cdot m^{-1}}$) was calculated from A ($\mathrm{kJ \cdot mol^{-1} \cdot Å^{-2}}$) as follows: $k = (1 \times 10^{23} \cdot N_A^{-1}) \cdot A$, where N_A is the Avogadro constant.

Table S1. Standard Orientation of the Optimized Geometry for 7H-ImD.

	X	Y	Z		X	Y	Z		X	Y	Z
C	1.1694520	2.2507760	-0.1500660	H	0.4219550	1.4674890	2.3446720	C	-4.5738990	-2.9523140	-1.2381300
C	3.9350070	2.1343610	-0.5323900	H	4.1630780	-0.1553940	4.7917710	C	-2.9911590	-2.7247270	-3.0523780
C	1.7529760	2.8807660	-1.2313890	H	6.4897480	-0.7752040	4.2141960	C	-5.4480730	-3.6164530	-2.0911160
C	1.9869950	1.6626090	0.8482600	H	6.8092000	-0.5250560	-4.0064930	H	-4.8565250	-2.7559130	-0.2123420
C	3.3616500	1.4759710	0.5766970	H	8.4368190	-0.9469520	-2.1911570	C	-3.8630730	-3.3998480	-3.8975020
C	3.1475020	2.8663980	-1.3931890	H	2.1110640	0.1346890	-4.2264150	H	-2.0366950	-2.3605090	-3.4093280
C	4.1244610	0.6239490	1.4656720	H	4.5165860	-0.1195280	-4.7378100	C	-5.0920890	-3.8464670	-3.4174260
C	3.6561070	0.4617250	2.7791490	H	3.3830160	-1.8277820	0.7665990	H	-6.4082510	-3.9516220	-1.7203930
C	2.3046940	0.8188170	3.0922980	H	0.9889220	-1.6281510	1.2852260	H	-3.5862870	-3.5751490	-4.9291530
C	1.4686030	1.2914970	2.1315150	H	0.2798830	0.1134300	-2.5552170	H	-5.7724650	-4.3701580	-4.0771240
C	4.5220720	-0.0836690	3.7719850	H	8.2233460	-1.1439040	2.5397610	C	-3.7055180	0.9368670	0.2652200
C	5.7953250	-0.4441500	3.4512970	H	8.9502860	-1.1674200	0.1747660	C	-4.5881780	0.8671460	-0.8161770
C	6.2155040	-0.4730560	2.0884830	C	-0.3060110	2.2617980	-0.0416620	C	-4.0818050	0.3821190	1.4907980
C	5.3160660	-0.0871480	1.0728860	C	-2.2972080	3.0736210	0.1062020	C	-5.8281420	0.2549360	-0.6724030
C	5.6291250	-0.4704970	-0.2913730	C	-2.4478840	1.7065820	0.1402350	H	-4.2914340	1.3009340	-1.7622990
C	6.9799130	-0.7285710	-0.6071610	C	-0.9325720	-0.2693230	-0.1786920	C	-5.3186210	-0.2408620	1.6325800
C	7.9269980	-0.9310640	0.4410800	C	-2.1059660	-1.9472440	0.6085440	H	-3.4028980	0.4587730	2.3307400
C	7.5326140	-0.8985220	1.7419560	C	-2.4170920	-1.7422630	-0.8565400	C	-6.1963310	-0.2975670	0.5524230
C	4.6567160	-0.5934280	-1.3519400	N	-1.1639840	1.1720260	0.0408420	H	-6.5048790	0.2059840	-1.5158620
C	5.0974470	-0.4805370	-2.6811550	N	-0.9682780	3.3893110	-0.0108630	H	-5.5998610	-0.6688830	2.5866800
C	6.4893370	-0.5734450	-2.9723920	N	-1.2979850	-1.0395050	1.0083190	H	-7.1639170	-0.7711130	0.6647200
C	7.3881130	-0.7857100	-1.9719150	N	-1.7670680	-0.7329500	-1.2948380	C	-3.3038550	4.1497480	0.1700990
C	3.2417970	-0.8016110	-1.1280830	C	-2.5968230	-3.0094050	1.5054040	C	-2.9348610	5.4229760	-0.2802450
C	2.3315160	-0.4140820	-2.1375850	C	-2.8382100	-4.3040430	1.0376520	C	-4.5973890	3.9642190	0.6716210
C	2.8217140	-0.1294980	-3.4524970	C	-2.7575950	-2.7253620	2.8654820	C	-3.8391390	6.4766780	-0.2489480
C	4.1448430	-0.2527740	-3.7287480	C	-3.2492800	-5.2974600	1.9203270	H	-1.9279060	5.5679000	-0.6468670
C	2.7135100	-1.3648940	0.0548680	H	-2.6919930	-4.5380880	-0.0083000	C	-5.4999570	5.0221620	0.6997360
C	1.3722210	-1.2655560	0.3433560	C	-3.1798960	-3.7167590	3.7405490	H	-4.9009160	2.9994610	1.0524770
C	0.5061610	-0.6126200	-0.5584240	H	-2.5401140	-1.7266140	3.2193220	C	-5.1292180	6.2807610	0.2369260
C	0.9632510	-0.2905900	-1.8182430	C	-3.4289780	-5.0041560	3.2686070	H	-3.5350000	7.4534860	-0.6043060
H	5.0034500	2.0713370	-0.6893370	H	-3.4264550	-6.3000960	1.5532940	H	-6.4959740	4.8603440	1.0931440
H	1.1162020	3.3760630	-1.9528420	H	-3.3099140	-3.4888590	4.7907010	H	-5.8352090	7.1014730	0.2611610
H	3.5988300	3.3931350	-2.2239330	H	-3.7558110	-5.7776990	3.9519020				
H	1.9463860	0.6422940	4.0995470	C	-3.3387080	-2.5066210	-1.7167590				

SCF Done: E(RM052X) = -2526.94943972 A.U.
Low frequencies --- -6.5399 -3.9851 -0.8840 -0.0010 -0.0007 0.0011
Low frequencies --- 6.4034 15.1868 20.8673

Zero-point correction = 0.800710 (Hartree/Particle)
Thermal correction to Energy = 0.845289
Thermal correction to Enthalpy = 0.846233
Thermal correction to Gibbs Free Energy = 0.721379
Sum of electronic and zero-point Energies = -2526.148729
Sum of electronic and thermal Energies = -2526.104150
Sum of electronic and thermal Enthalpies = -2526.103206
Sum of electronic and thermal Free Energies = -2526.228061

Excitation energies and oscillator strengths:

Excited State 1: Singlet-A 2.6303 eV 471.37 nm *f*=0.0012 <S**2>=0.000
211 -> 213 -0.13962
212 -> 213 0.68649
This state for optimization and/or second-order correction.
Total Energy, E(TD-HF/TD-KS) = -2526.56329921
Copying the excited state density for this state as the 1-particle RhoCI density.

Excited State 2: Singlet-A 2.9368 eV 422.18 nm *f*=0.0143 <S**2>=0.000
210 -> 214 -0.10997
210 -> 215 -0.17449
211 -> 213 0.58234
211 -> 214 0.13126

211 -> 215 -0.15684
212 -> 213 0.10563
212 -> 214 0.20361
212 -> 215 -0.11296

Excited State 3: Singlet-A 3.0180 eV 410.81 nm *f*=0.0124 <S**2>=0.000
210 -> 213 -0.15350
210 -> 214 -0.23649
210 -> 215 -0.11131
211 -> 213 -0.23952
211 -> 214 0.27148
212 -> 214 0.48460
212 -> 215 0.14003

Excited State 4: Singlet-A 3.1031 eV 399.55 nm *f*=0.0141 <S**2>=0.000
210 -> 213 0.57640
210 -> 214 0.12455
210 -> 215 -0.22489
211 -> 213 -0.18291
211 -> 214 0.13145
211 -> 215 -0.13860

Excited State 5: Singlet-A 3.1237 eV 396.91 nm *f*=0.0364 <S**2>=0.000
210 -> 213 0.21176
210 -> 214 0.17596
211 -> 213 0.19826
211 -> 215 0.38416
212 -> 215 0.46526

Excited State 6: Singlet-A 3.2826 eV 377.70 nm *f*=0.0007 <S**2>=0.000
210 -> 213 0.20664
210 -> 214 -0.17908
210 -> 215 0.33373
211 -> 214 -0.26097
211 -> 215 0.22501
212 -> 214 0.25973
212 -> 215 -0.29588

Excited State 7: Singlet-A 3.3339 eV 371.89 nm *f*=0.0179 <S**2>=0.000
209 -> 213 0.12530
210 -> 213 -0.19150
210 -> 214 0.54096
210 -> 215 -0.14235
212 -> 214 0.23625
212 -> 215 -0.19421

Excited State 8: Singlet-A 3.4057 eV 364.05 nm *f*=0.0447 <S**2>=0.000
211 -> 214 0.39502
211 -> 215 0.39672
212 -> 214 -0.23852
212 -> 215 -0.30363

Excited State 9: Singlet-A 3.4389 eV 360.53 nm *f*=0.0138 <S**2>=0.000
209 -> 213 0.66910
212 -> 214 -0.10060

Excited State 10: Singlet-A 3.5613 eV 348.14 nm *f*=0.1255 <S**2>=0.000
208 -> 213 0.44348
210 -> 214 -0.13839
210 -> 215 -0.33985
211 -> 214 -0.28563
211 -> 215 0.20682

Excited State 11: Singlet-A 3.5984 eV 344.55 nm *f*=0.0135 <S**2>=0.000
208 -> 213 0.50712
210 -> 214 0.13650
210 -> 215 0.34305
211 -> 214 0.23050

Excited State 12: Singlet-A 3.7179 eV 333.48 nm *f*=0.0195 <S**2>=0.000

208 -> 215 -0.23413
209 -> 214 0.43334
209 -> 215 -0.31249
210 -> 217 0.18832
211 -> 214 0.11101
211 -> 218 0.10784
212 -> 214 -0.10174
212 -> 217 -0.16902

Excited State 13: Singlet-A 3.8032 eV 326.00 nm *f*=0.0049 <S**2>=0.000
208 -> 214 0.32086
209 -> 214 0.41798
209 -> 215 0.39106
212 -> 217 0.14394

Excited State 14: Singlet-A 3.8546 eV 321.65 nm *f*=0.0534 <S**2>=0.000
207 -> 213 -0.10793
208 -> 214 -0.34554
211 -> 217 0.19507
212 -> 216 -0.13018
212 -> 217 0.50191

Excited State 15: Singlet-A 3.8862 eV 319.03 nm *f*=0.0307 <S**2>=0.000
194 -> 213 -0.10345
196 -> 213 0.11318
201 -> 213 -0.13961
204 -> 213 -0.14969
205 -> 213 0.46709
206 -> 213 0.12234
207 -> 213 -0.34194
208 -> 214 0.15557

Excited State 16: Singlet-A 3.9243 eV 315.94 nm *f*=0.0018 <S**2>=0.000
208 -> 215 0.13103
212 -> 216 0.64599
212 -> 217 0.10684

Excited State 17: Singlet-A 3.9467 eV 314.14 nm *f*=0.0096 <S**2>=0.000
208 -> 214 0.13188
208 -> 215 0.50915
209 -> 214 0.15510
209 -> 215 -0.25604
211 -> 217 0.11024
211 -> 218 -0.12342
212 -> 216 -0.18751
212 -> 218 -0.13299

Excited State 18: Singlet-A 3.9788 eV 311.62 nm *f*=0.0129 <S**2>=0.000
203 -> 213 -0.15322
205 -> 213 -0.29036
206 -> 213 0.52235
207 -> 213 -0.24565
208 -> 215 -0.11206

Excited State 19: Singlet-A 4.0028 eV 309.74 nm *f*=0.1115 <S**2>=0.000
208 -> 214 0.31061
208 -> 215 -0.23461
209 -> 215 -0.31463
210 -> 217 -0.12331
210 -> 218 -0.10127
211 -> 217 -0.22012
211 -> 218 -0.11473
212 -> 217 0.32682

Excited State 20: Singlet-A 4.0241 eV 308.11 nm *f*=0.0092 <S**2>=0.000
202 -> 213 -0.24138
204 -> 213 -0.10808
205 -> 213 0.27076
206 -> 213 0.33660
207 -> 213 0.47153

Excited State 21: Singlet-A 4.0567 eV 305.63 nm *f*=0.0198 <S**2>=0.000
208 -> 214 0.24470
209 -> 214 -0.17362
210 -> 217 0.33719
210 -> 218 -0.15156
211 -> 216 -0.14100
211 -> 217 0.21913
211 -> 218 0.15919
212 -> 218 0.34847

Excited State 22: Singlet-A 4.1015 eV 302.29 nm *f*=0.0105 <S**2>=0.000
194 -> 213 0.22567
195 -> 213 -0.10433
196 -> 213 -0.17677
197 -> 213 0.10669
199 -> 213 0.12173
200 -> 213 -0.14068
201 -> 213 0.37821
202 -> 213 -0.23161
203 -> 213 0.25349
205 -> 213 0.11869
207 -> 213 -0.20005

Excited State 23: Singlet-A 4.1244 eV 300.61 nm *f*=0.0271 <S**2>=0.000
208 -> 215 -0.18061
210 -> 217 -0.21462
210 -> 218 -0.22439
211 -> 217 0.43215
211 -> 218 -0.14968
212 -> 217 -0.10100
212 -> 219 -0.14021
212 -> 220 -0.21202

Excited State 24: Singlet-A 4.1508 eV 298.70 nm *f*=0.0022 <S**2>=0.000
202 -> 213 0.20088
203 -> 213 0.39990
204 -> 213 0.36003
206 -> 213 0.13194
206 -> 214 0.18832
206 -> 215 -0.13121
207 -> 213 0.10547
212 -> 218 -0.12705

Excited State 25: Singlet-A 4.1876 eV 296.08 nm *f*=0.0228 <S**2>=0.000
202 -> 213 0.12875
203 -> 213 0.13326
206 -> 214 -0.26014
207 -> 214 0.13420
210 -> 218 -0.10205
211 -> 217 0.13515
211 -> 218 -0.14478
211 -> 220 -0.13002
212 -> 217 -0.10767
212 -> 219 0.26104
212 -> 220 0.36203

Excited State 26: Singlet-A 4.2087 eV 294.59 nm *f*=0.0148 <S**2>=0.000
203 -> 213 -0.24522
206 -> 213 -0.12346
206 -> 214 0.29558
207 -> 214 -0.15343
210 -> 217 -0.14592
211 -> 216 -0.25026
211 -> 217 0.16485
212 -> 218 -0.11812
212 -> 219 0.19208
212 -> 220 0.25983

Excited State 27: Singlet-A 4.2223 eV 293.64 nm *f*=0.0131 <S**2>=0.000

204 -> 213 0.13984
206 -> 213 0.11620
206 -> 215 0.39804
207 -> 215 -0.20440
210 -> 217 -0.33052
211 -> 216 -0.17630
211 -> 217 -0.12157
212 -> 218 0.22168

Excited State 28: Singlet-A 4.2421 eV 292.27 nm *f*=0.0047 <S**2>=0.000
203 -> 213 -0.18744
204 -> 213 0.30101
204 -> 214 0.12375
206 -> 214 0.10165
206 -> 215 0.13154
211 -> 216 0.49000

Excited State 29: Singlet-A 4.2534 eV 291.50 nm *f*=0.0602 <S**2>=0.000
202 -> 213 -0.16818
203 -> 213 -0.12947
204 -> 213 0.35335
205 -> 214 0.11448
206 -> 214 -0.25009
207 -> 214 0.12479
210 -> 217 0.15344
211 -> 216 -0.31689
212 -> 218 -0.20801

Excited State 30: Singlet-A 4.2617 eV 290.93 nm *f*=0.0451 <S**2>=0.000
194 -> 213 0.12609
200 -> 213 -0.21481
201 -> 213 0.22669
202 -> 213 0.48787
203 -> 213 -0.21343
205 -> 213 0.13981

Excited State 31: Singlet-A 4.3293 eV 286.38 nm *f*=0.0576 <S**2>=0.000
203 -> 213 -0.17075
204 -> 213 0.22378
204 -> 214 -0.17471
205 -> 213 0.12290
205 -> 214 -0.14392
206 -> 215 -0.27360
207 -> 215 0.15208
210 -> 217 -0.12202
211 -> 218 -0.20788
212 -> 218 0.37026

Excited State 32: Singlet-A 4.3713 eV 283.64 nm *f*=0.0354 <S**2>=0.000
204 -> 214 -0.11176
210 -> 216 0.51075
210 -> 218 -0.11930
211 -> 218 0.16516
212 -> 219 0.19127
212 -> 220 -0.16265
212 -> 221 0.23853

Excited State 33: Singlet-A 4.3742 eV 283.45 nm *f*=0.0070 <S**2>=0.000
210 -> 216 -0.35275
211 -> 221 -0.13907
212 -> 219 0.29409
212 -> 220 -0.27066
212 -> 221 0.37324

Excited State 34: Singlet-A 4.4108 eV 281.09 nm *f*=0.0693 <S**2>=0.000
199 -> 213 -0.34510
200 -> 213 0.42466
201 -> 213 0.38902

Excited State 35: Singlet-A 4.4225 eV 280.35 nm *f*=0.0807 <S**2>=0.000

203 -> 214 0.10057
204 -> 213 0.11407
204 -> 214 -0.24743
204 -> 215 0.10053
205 -> 214 -0.18480
206 -> 214 -0.11632
210 -> 216 -0.24025
210 -> 217 -0.13941
210 -> 218 -0.15217
211 -> 218 0.39315
212 -> 218 -0.10430

Excited State 36: Singlet-A 4.4362 eV 279.48 nm *f*=0.0019 <S**2>=0.000
211 -> 221 0.14280
212 -> 219 0.47382
212 -> 220 -0.20250
212 -> 221 -0.42828

Excited State 37: Singlet-A 4.4813 eV 276.67 nm *f*=0.0463 <S**2>=0.000
194 -> 213 0.14530
199 -> 213 0.39132
200 -> 213 0.35240
204 -> 215 0.16280
205 -> 215 0.14807
210 -> 218 0.24743

Excited State 38: Singlet-A 4.4890 eV 276.20 nm *f*=0.0662 <S**2>=0.000
199 -> 213 -0.32111
200 -> 213 -0.20661
203 -> 215 -0.13812
204 -> 215 0.24864
205 -> 215 0.23906
208 -> 217 0.11643
210 -> 216 0.13490
210 -> 218 0.32375
211 -> 218 0.12197

Excited State 39: Singlet-A 4.5413 eV 273.02 nm *f*=0.1449 <S**2>=0.000
204 -> 215 -0.12643
205 -> 215 -0.10466
208 -> 217 0.10288
209 -> 217 0.30533
211 -> 218 0.13724
211 -> 220 0.11588
211 -> 221 -0.10764
211 -> 222 0.13963
212 -> 222 0.43549
212 -> 223 0.19136

Excited State 40: Singlet-A 4.5838 eV 270.49 nm *f*=0.0713 <S**2>=0.000
208 -> 217 0.42829
209 -> 216 -0.14414
209 -> 217 0.27487
209 -> 218 0.15586
210 -> 218 -0.13441
212 -> 218 -0.11204
212 -> 222 -0.15666

Table S2. Standard Orientation of the Optimized Geometry for 9H-ImD.

	X	Y	Z		X	Y	Z		X	Y	Z
C	-2.5990110	4.2813600	0.8976000	C	-6.6918050	-0.5446280	-0.1557110	C	3.9583300	6.3292700	-1.7524350
H	-2.3878240	5.1644010	1.4876210	C	-5.7137480	0.4699270	2.2803810	H	2.1661520	5.1961050	-2.1270310
C	-1.5691670	3.6112900	0.2777440	C	-2.9557500	0.8401060	2.6754290	C	6.1730870	0.2727630	-0.3164600
H	-0.5536190	3.9727610	0.3528280	C	-1.0698350	-0.2985890	0.9099760	H	4.6794910	0.8604750	-1.7571040
C	-0.7863440	1.7948080	-1.2560680	C	-1.8092220	-2.4752150	-0.7369600	C	5.5495630	0.5572890	1.9954470
C	-1.1253990	0.9291580	-2.2477790	C	-4.2015400	-3.8310140	-0.2872900	H	3.5704980	1.3291160	2.3431010
H	-0.3530830	0.4941870	-2.8725250	C	-4.1946350	2.7223000	-0.0127500	C	3.4553730	-2.3986290	1.9346840
C	-2.8191980	-0.4768030	-3.4092770	C	-3.1524670	1.9868980	-0.6197360	C	3.6241470	-2.8405680	-1.3769880
H	-2.0845650	-0.7144890	-4.1695650	C	-3.4070370	0.7938710	-1.4035740	C	5.1708370	6.3755750	-1.0695940
C	-4.0792810	-0.9924250	-3.4644220	C	-4.5847610	-0.0173470	-1.2757430	H	6.4566080	5.3518090	0.3151790
H	-4.3896310	-1.6192990	-4.2916820	C	-5.3512530	-0.1307640	-0.0573620	H	3.6705350	7.1446750	-2.4045630
C	-6.2977800	-1.3533270	-2.4033790	C	-4.8096010	0.1204220	1.2608300	C	6.4869810	0.1782210	1.0356480
H	-6.6127510	-1.9017340	-3.2831570	C	-3.3997840	0.0659250	1.5878210	H	6.8974990	-0.0185040	-1.0667520
C	-7.1530140	-1.1490460	-1.3642620	C	-2.4204350	-0.7042140	0.8584050	H	5.7864540	0.4729640	3.0484070
H	-8.1779760	-1.4975970	-1.4105110	C	-2.7539850	-1.9319830	0.1599550	C	4.7800190	-2.8093930	1.7600880
C	-7.5655140	-0.3633930	0.9597090	C	-3.9341450	-2.6718420	0.4054440	C	2.8223060	-2.5988290	3.1653210
H	-8.6066480	-0.6443190	0.8545950	H	-5.1117180	-4.3784040	-0.0780540	C	4.1959890	-2.4434270	-2.5887040
C	-7.1093520	0.2195630	2.1013390	H	-4.6238280	-2.3397590	1.1671310	C	3.7818930	-4.1561540	-0.9325760
H	-7.7843730	0.4510030	2.9167170	H	-5.2179260	2.4059580	-0.1593270	H	5.8324460	7.2249150	-1.1858730
C	-5.2268870	1.0813360	3.4750450	H	-4.7389470	4.4014370	1.1823660	H	7.4564880	-0.1930970	1.3438240
H	-5.9414990	1.3832210	4.2315920	C	0.6324950	2.0358520	-0.9270320	C	5.4594390	-3.4204880	2.8079690
C	-3.9014000	1.3509870	3.6154610	N	1.2269630	3.1878860	-1.0939200	H	5.2843020	-2.6222890	0.8218630
H	-3.5321700	1.9067660	4.4690330	N	1.4868750	1.1066780	-0.3571580	C	3.5024480	-3.2194120	4.2051920
C	-1.5651610	1.1015340	2.8225430	C	2.5202410	3.0386810	-0.6619010	H	1.8005840	-2.2634150	3.2867790
H	-1.2409170	1.7341810	3.6400340	C	2.7140020	1.7539740	-0.2071030	C	4.9369610	-3.3483270	-3.3378600
C	-0.6664180	0.6361040	1.9120750	C	1.3099180	-0.3372570	-0.1404980	H	4.0482080	-1.4263850	-2.9270280
H	0.3746810	0.9102000	1.9886320	C	3.4611750	4.1695940	-0.7692750	C	4.5129700	-5.0621670	-1.6932650
C	-0.1418290	-0.8402650	-0.0599980	C	3.9835060	1.1395950	0.2468290	H	3.3256560	-4.4716870	-0.0031990
C	-0.5320080	-1.8533160	-0.8790890	N	1.9019570	-0.7438760	1.1402380	C	4.8209750	-3.6323260	4.0270900
H	0.1355850	-2.2286550	-1.6415850	N	2.0169450	-1.0530590	-1.2046230	H	6.4883430	-3.7281840	2.6725250
C	-2.1135960	-3.6487000	-1.4606440	C	4.6759890	4.2291310	-0.0766650	H	3.0055980	-3.3809950	5.1530660
H	-1.3824820	-4.0270270	-2.1657000	C	3.1114430	5.2384930	-1.6037170	C	5.0967180	-4.6583230	-2.8905620
C	-3.2990160	-4.3124380	-1.2532200	C	4.9271850	0.7572490	-0.7079360	H	5.3859400	-3.0345010	-4.2714680
H	-3.5235650	-5.2171770	-1.8036350	C	4.3050400	1.0361460	1.6040040	H	4.6252660	-6.0824950	-1.3502930
C	-3.9250960	3.8468430	0.7326260	C	2.7201040	-1.6893090	0.8743680	H	5.3509070	-4.1153500	4.8384400
C	-1.8281740	2.4727160	-0.5157460	C	2.8037630	-1.8756330	-0.6223600	H	5.6718180	-5.3637620	-3.4768180
C	-2.4626810	0.4372450	-2.3768640	C	5.5214770	5.3228330	-0.2303900				
C	-4.9882180	-0.7863070	-2.3858090	H	4.9623090	3.4303540	0.5922610				

SCF Done: E(RM052X) = -2834.21324968 A.U.
Low frequencies --- -5.8942 -3.3233 -0.0008 -0.0006 0.0006 4.8744
Low frequencies --- 10.8616 16.2306 20.4494

Zero-point correction = 0.896190 (Hartree/Particle)
Thermal correction to Energy = 0.946121
Thermal correction to Enthalpy = 0.947065
Thermal correction to Gibbs Free Energy = 0.812530
Sum of electronic and zero-point Energies = -2833.317059
Sum of electronic and thermal Energies = -2833.267129
Sum of electronic and thermal Enthalpies = -2833.266185
Sum of electronic and thermal Free Energies = -2833.400720

Excitation energies and oscillator strengths:

Excited State 1: Singlet-A 2.5433 eV 487.50 nm *f*=0.0009 <S**2>=0.000
236 -> 239 0.18852
238 -> 239 0.67246
This state for optimization and/or second-order correction.
Total Energy, E(TD-HF/TD-KS) = -2833.78618275
Copying the excited state density for this state as the 1-particle RhoCI density.

Excited State 2: Singlet-A 2.7382 eV 452.80 nm *f*=0.0051 <S**2>=0.000

```
      235 -> 239         -0.37892
      236 -> 239         -0.15311
      237 -> 239          0.54025

Excited State   3:      Singlet-A      2.8177 eV  440.03 nm  f=0.0014  <S**2>=0.000
      235 -> 239         -0.12208
      236 -> 239          0.63472
      237 -> 239          0.12293
      238 -> 239         -0.15389
      238 -> 241         -0.14345

Excited State   4:      Singlet-A      2.8465 eV  435.57 nm  f=0.0006  <S**2>=0.000
      235 -> 239          0.54844
      236 -> 240          0.10268
      237 -> 239          0.32464
      237 -> 240          0.10799
      237 -> 241          0.20171
      238 -> 240         -0.13784

Excited State   5:      Singlet-A      2.9630 eV  418.44 nm  f=0.0034  <S**2>=0.000
      235 -> 239          0.12392
      236 -> 240         -0.22412
      237 -> 239          0.18576
      237 -> 240          0.13075
      237 -> 241         -0.21043
      238 -> 240          0.50770
      238 -> 241         -0.22938

Excited State   6:      Singlet-A      3.0559 eV  405.72 nm  f=0.0151  <S**2>=0.000
      237 -> 239         -0.16279
      237 -> 240          0.59616
      237 -> 241          0.22287
      238 -> 240          0.12611
      238 -> 241          0.15726

Excited State   7:      Singlet-A      3.0882 eV  401.47 nm  f=0.0223  <S**2>=0.000
      235 -> 240         -0.22600
      236 -> 239          0.15686
      236 -> 240          0.11740
      236 -> 241         -0.21166
      237 -> 240         -0.10512
      237 -> 241         -0.13507
      238 -> 240          0.25954
      238 -> 241          0.49395

Excited State   8:      Singlet-A      3.1818 eV  389.66 nm  f=0.0086  <S**2>=0.000
      234 -> 239         -0.20405
      234 -> 241         -0.10365
      235 -> 240          0.33608
      235 -> 241         -0.12009
      236 -> 240         -0.31279
      237 -> 239          0.10023
      237 -> 240          0.13426
      237 -> 241         -0.26763
      238 -> 240         -0.19885
      238 -> 241          0.25112

Excited State   9:      Singlet-A      3.2605 eV  380.26 nm  f=0.0093  <S**2>=0.000
      234 -> 239          0.63418
      237 -> 241         -0.14673
      238 -> 240         -0.14125

Excited State  10:      Singlet-A      3.2807 eV  377.92 nm  f=0.0482  <S**2>=0.000
      235 -> 241         -0.34812
      236 -> 241          0.44692
      237 -> 240         -0.15248
      237 -> 241          0.27866
      238 -> 240          0.11237
      238 -> 241          0.19801
```

Excited State 11: Singlet-A 3.4008 eV 364.57 nm *f*=0.0126 <S**2>=0.000
234 -> 239 0.14337
234 -> 240 0.19882
235 -> 240 0.26303
235 -> 241 0.21295
236 -> 240 -0.28628
236 -> 241 -0.21586
237 -> 240 -0.19575
237 -> 241 0.31758
238 -> 240 0.15568

Excited State 12: Singlet-A 3.5312 eV 351.11 nm *f*=0.0029 <S**2>=0.000
235 -> 240 0.42994
235 -> 241 -0.26648
236 -> 240 0.40666
236 -> 241 -0.15778
238 -> 240 0.15000

Excited State 13: Singlet-A 3.5365 eV 350.59 nm *f*=0.0172 <S**2>=0.000
235 -> 240 0.21641
235 -> 241 0.45336
236 -> 240 0.20761
236 -> 241 0.37384
237 -> 241 -0.14299
238 -> 241 0.14585

Excited State 14: Singlet-A 3.6426 eV 340.37 nm *f*=0.0198 <S**2>=0.000
232 -> 239 0.17948
233 -> 239 0.14072
234 -> 240 0.31848
234 -> 241 0.48864
238 -> 242 0.11399
238 -> 243 0.11694
238 -> 244 0.12364

Excited State 15: Singlet-A 3.6524 eV 339.46 nm *f*=0.0013 <S**2>=0.000
234 -> 240 0.54333
234 -> 241 -0.32485
235 -> 241 -0.10788
237 -> 241 -0.13664

Excited State 16: Singlet-A 3.7135 eV 333.87 nm *f*=0.0337 <S**2>=0.000
229 -> 239 -0.17920
232 -> 239 0.45149
233 -> 239 0.40198
234 -> 239 0.12062
234 -> 241 -0.19409

Excited State 17: Singlet-A 3.7415 eV 331.38 nm *f*=0.0058 <S**2>=0.000
229 -> 239 0.10029
232 -> 239 -0.37159
233 -> 239 0.46754
234 -> 241 0.11704
238 -> 242 -0.23166
238 -> 243 -0.14603

Excited State 18: Singlet-A 3.7703 eV 328.84 nm *f*=0.0677 <S**2>=0.000
232 -> 239 -0.16411
233 -> 239 0.23143
233 -> 240 -0.12704
234 -> 240 -0.13717
234 -> 241 -0.13731
237 -> 243 0.14020
237 -> 244 -0.10269
238 -> 242 0.43000
238 -> 243 0.31018

Excited State 19: Singlet-A 3.8218 eV 324.41 nm *f*=0.0156 <S**2>=0.000
229 -> 239 0.19977
230 -> 239 -0.10615

231 -> 239 -0.15436
234 -> 241 -0.15043
237 -> 242 0.30482
237 -> 243 0.36067
238 -> 243 -0.16343
238 -> 244 0.27904

Excited State 20: Singlet-A 3.8362 eV 323.20 nm *f*=0.0013 <S**2>=0.000
229 -> 239 0.41868
230 -> 239 -0.23647
231 -> 239 -0.35594
232 -> 239 0.12210
237 -> 242 -0.15456
237 -> 243 -0.19816
238 -> 244 -0.10468

Excited State 21: Singlet-A 3.8859 eV 319.06 nm *f*=0.0107 <S**2>=0.000
236 -> 242 0.28607
236 -> 243 0.12614
237 -> 242 -0.12429
237 -> 243 -0.16253
238 -> 242 0.40267
238 -> 243 -0.31376
238 -> 244 0.15011

Excited State 22: Singlet-A 3.9110 eV 317.02 nm *f*=0.0414 <S**2>=0.000
217 -> 239 0.11775
218 -> 239 0.12075
219 -> 239 0.16516
223 -> 239 0.10512
225 -> 239 -0.21634
227 -> 239 0.30723
228 -> 239 0.38006
229 -> 239 -0.20232
231 -> 239 -0.19254

Excited State 23: Singlet-A 3.9481 eV 314.04 nm *f*=0.0166 <S**2>=0.000
230 -> 239 0.13394
232 -> 239 -0.10090
232 -> 241 0.17384
233 -> 240 0.27365
234 -> 242 -0.10072
234 -> 243 -0.13713
235 -> 242 -0.10791
235 -> 244 -0.16931
237 -> 242 -0.17711
237 -> 244 -0.21125
237 -> 246 0.11314
238 -> 243 0.24706
238 -> 244 0.20436
238 -> 246 0.14679

Excited State 24: Singlet-A 3.9730 eV 312.07 nm *f*=0.0167 <S**2>=0.000
227 -> 239 -0.15243
228 -> 239 0.25748
229 -> 239 0.14080
230 -> 239 -0.42263
231 -> 239 0.43039

Excited State 25: Singlet-A 3.9915 eV 310.62 nm *f*=0.0255 <S**2>=0.000
233 -> 240 -0.14934
235 -> 242 -0.22542
235 -> 243 -0.21276
236 -> 242 0.10960
236 -> 243 0.31931
237 -> 242 0.12898
237 -> 243 -0.14661
237 -> 244 0.26596
238 -> 242 -0.11413
238 -> 243 0.24548

238 -> 244 0.15760

Excited State 26: Singlet-A 4.0094 eV 309.24 nm *f*=0.0125 <S**2>=0.000
225 -> 239 -0.11024
227 -> 239 0.18294
229 -> 239 0.34367
230 -> 239 0.36142
231 -> 239 0.27088
232 -> 239 0.12257
235 -> 242 0.11226
238 -> 244 -0.11144

Excited State 27: Singlet-A 4.0247 eV 308.06 nm *f*=0.0249 <S**2>=0.000
232 -> 240 -0.17695
233 -> 240 -0.21833
233 -> 241 -0.14187
236 -> 242 -0.27260
236 -> 243 -0.12003
237 -> 242 -0.19905
237 -> 243 -0.12229
238 -> 244 0.39925

Excited State 28: Singlet-A 4.0537 eV 305.86 nm *f*=0.0193 <S**2>=0.000
227 -> 239 0.12050
228 -> 239 -0.15324
232 -> 240 0.30413
232 -> 241 -0.22854
233 -> 240 -0.14238
233 -> 241 -0.16488
236 -> 242 0.27069
236 -> 243 0.11312
237 -> 242 -0.23071
237 -> 243 0.11650
237 -> 244 -0.17380

Excited State 29: Singlet-A 4.0771 eV 304.10 nm *f*=0.0055 <S**2>=0.000
227 -> 239 -0.13219
228 -> 239 0.10731
232 -> 240 -0.16487
233 -> 241 0.12494
235 -> 242 0.46485
236 -> 242 0.18101
236 -> 243 0.10148
237 -> 242 -0.22817
237 -> 243 0.13868
237 -> 244 0.14645
238 -> 243 0.12967
238 -> 244 0.12262

Excited State 30: Singlet-A 4.0933 eV 302.90 nm *f*=0.0374 <S**2>=0.000
227 -> 239 0.22753
228 -> 239 -0.19493
230 -> 239 -0.10898
232 -> 240 0.15888
232 -> 241 0.13210
233 -> 240 -0.16110
234 -> 243 -0.10677
235 -> 242 0.16816
235 -> 243 0.17067
236 -> 243 -0.11156
236 -> 244 0.17520
237 -> 243 -0.12485
237 -> 244 0.29975
238 -> 243 0.11839
238 -> 246 0.10240

Excited State 31: Singlet-A 4.1028 eV 302.20 nm *f*=0.0147 <S**2>=0.000
227 -> 239 0.18694
228 -> 239 -0.17459
232 -> 240 -0.26959

232 -> 241 -0.10583
233 -> 239 -0.10764
233 -> 241 0.41069
235 -> 244 -0.16877
236 -> 242 0.10877
236 -> 244 0.14053
237 -> 244 -0.21009

Excited State 32: Singlet-A 4.1090 eV 301.74 nm *f*=0.0006 <S**2>=0.000
227 -> 239 0.22540
228 -> 239 -0.29321
229 -> 239 -0.10226
230 -> 239 -0.21793
233 -> 240 0.30877
235 -> 243 -0.11633
235 -> 244 0.17999
236 -> 242 -0.14258
236 -> 243 0.17730
236 -> 244 -0.21849

Excited State 33: Singlet-A 4.1630 eV 297.83 nm *f*=0.0064 <S**2>=0.000
232 -> 241 -0.10097
233 -> 240 0.10905
233 -> 241 -0.10341
235 -> 242 0.17964
235 -> 243 0.33739
236 -> 243 0.23287
237 -> 242 0.22671
237 -> 243 -0.25191
237 -> 244 -0.22008
237 -> 246 -0.10934
238 -> 247 -0.13224
238 -> 248 0.14826

Excited State 34: Singlet-A 4.1927 eV 295.71 nm *f*=0.0095 <S**2>=0.000
232 -> 240 -0.17371
233 -> 241 -0.17040
235 -> 244 0.11819
236 -> 242 0.34560
236 -> 243 -0.30360
236 -> 244 -0.20547
237 -> 242 0.19541
237 -> 243 -0.15251
237 -> 244 -0.13219
237 -> 248 0.10650
238 -> 242 -0.11177
238 -> 247 0.10423
238 -> 248 -0.10459

Excited State 35: Singlet-A 4.2080 eV 294.64 nm *f*=0.0057 <S**2>=0.000
235 -> 242 -0.30663
235 -> 243 0.37709
235 -> 244 0.17477
236 -> 242 0.18395
236 -> 244 -0.15843
237 -> 242 -0.16996
237 -> 244 0.10207
237 -> 246 0.10467
237 -> 248 -0.10420
238 -> 247 -0.14804

Excited State 36: Singlet-A 4.2361 eV 292.68 nm *f*=0.0692 <S**2>=0.000
224 -> 239 -0.22968
225 -> 239 0.29738
226 -> 239 0.14685
227 -> 239 0.27381
228 -> 239 0.14617
233 -> 241 0.11521
236 -> 243 -0.10919
236 -> 246 -0.11035

238 -> 247 0.11538
238 -> 248 0.26054

Excited State 37: Singlet-A 4.2481 eV 291.86 nm *f*=0.0219 <S**2>=0.000
224 -> 239 0.14692
225 -> 239 -0.21056
227 -> 239 -0.19955
232 -> 240 0.10067
233 -> 241 0.20039
236 -> 244 -0.10693
236 -> 246 -0.16955
238 -> 246 0.30441
238 -> 247 0.14388
238 -> 248 0.28941

Excited State 38: Singlet-A 4.2774 eV 289.86 nm *f*=0.0031 <S**2>=0.000
232 -> 241 -0.25710
235 -> 243 -0.10161
235 -> 247 -0.11815
236 -> 244 -0.13761
236 -> 247 -0.12699
237 -> 246 0.17284
237 -> 247 0.10552
237 -> 248 0.11385
238 -> 244 -0.11266
238 -> 246 0.38718
238 -> 247 -0.19235
238 -> 248 -0.15768

Excited State 39: Singlet-A 4.2872 eV 289.20 nm *f*=0.0303 <S**2>=0.000
232 -> 241 -0.27220
234 -> 243 -0.10634
235 -> 243 0.22553
235 -> 247 0.16897
236 -> 243 0.13906
236 -> 247 0.19054
237 -> 246 0.24709
238 -> 245 -0.15044
238 -> 247 0.28552

Excited State 40: Singlet-A 4.3065 eV 287.90 nm *f*=0.0569 <S**2>=0.000
232 -> 241 0.27511
233 -> 240 -0.16357
235 -> 248 -0.11654
236 -> 243 0.18797
236 -> 244 -0.12729
236 -> 248 0.14955
237 -> 243 -0.10178
237 -> 244 -0.10635
237 -> 248 -0.22203
238 -> 246 0.22293
238 -> 247 0.10346
238 -> 248 -0.24406

Table S3. Standard Orientation of the Optimized Geometry for 7H-BR.

	X	Y	Z		X	Y	Z		X	Y	Z
C	1.1028660	-0.0266520	1.8847270	H	0.5649870	-2.5572960	0.9787260	C	-4.3877410	-1.3243310	-2.950570
C	3.8537670	0.5026670	1.9407250	H	4.5095110	-4.8391200	-0.244351	C	-2.8992140	-2.7380560	-1.670770
C	1.5968640	1.1578750	2.4292210	H	6.8974270	-4.2201550	-0.516648	C	-5.2169470	-2.4154850	-3.183422
C	2.0193240	-0.9972180	1.3575180	H	7.2527000	3.9682200	-0.086616	H	-4.6264120	-0.3605250	-3.381156
C	3.3937630	-0.6535820	1.2722030	H	8.8805920	2.1058920	-0.240769	C	-3.7353390	-3.8213960	-1.898800
C	2.9708640	1.3864810	2.5219250	H	2.5775870	4.4060170	-0.414066	H	-1.9957080	-2.8389300	-1.083787
C	4.3039890	-1.5122930	0.5406050	H	5.0083190	4.7747270	-0.116247	C	-4.8972380	-3.6622380	-2.652098
C	3.9005190	-2.8391040	0.3136940	H	3.9111460	-0.7608640	-1.869883	H	-6.1074290	-2.2935000	-3.787108
C	2.5334140	-3.1980170	0.5015750	H	1.5074350	-1.0762000	-2.285984	H	-3.4874660	-4.7877690	-1.479199
C	1.6076010	-2.2936540	0.9146930	H	0.7298990	2.9164530	-0.964766	H	-5.5485370	-4.5090050	-2.829790
C	4.8436580	-3.8169410	-0.113989	H	8.6277550	-2.5789820	-0.567767	C	-3.5126480	1.3913120	2.0962870
C	6.1473320	-3.4746630	-0.281505	H	9.3706750	-0.2178660	-0.425221	C	-4.6547600	1.0343810	2.8226680
C	6.5463790	-2.1073740	-0.222980	C	-0.3422440	-0.1665640	1.8586220	C	-3.4217180	2.6749590	1.5426410
C	5.5921370	-1.0929550	0.0276460	C	-2.3090280	-0.9787720	1.7680260	C	-5.6981940	1.9406650	2.9727420
C	5.9775580	0.2773650	-0.275179	C	-2.3693890	0.4857930	1.9484020	H	-4.7149240	0.0567860	3.2837270
C	7.3614490	0.5697880	-0.301311	C	-0.4660260	0.6627300	-1.684948	C	-4.4693340	3.5733660	1.6906790
C	8.3213090	-0.4803680	-0.365618	C	-2.5465630	1.0807010	-1.744694	H	-2.5330830	2.9362780	0.9827930
C	7.9164340	-1.7748050	-0.423340	C	-2.3022750	-0.3676330	-1.936874	C	-5.6105720	3.2075240	2.4016410
C	5.0630940	1.3715360	-0.543106	N	-1.1365780	0.9450730	1.9940140	H	-6.5747730	1.6601260	3.5429230
C	5.5298880	2.6885130	-0.354090	N	-1.0311460	-1.3303670	1.7261090	H	-4.4010610	4.5546750	1.2395300
C	6.9215840	2.9427220	-0.197072	N	-1.3757090	1.6761240	-1.596254	H	-6.4273710	3.9096220	2.5136720
C	7.8135400	1.9202610	-0.263834	N	-0.9980830	-0.5755610	-1.883145	C	-3.3632470	-1.9791350	1.6224000
C	3.7001580	1.2111600	-1.006383	C	-3.7900860	1.8496540	-1.685720	C	-3.0600040	-3.3198240	1.9068850
C	2.7985540	2.2997620	-0.877917	C	-3.7413160	3.2194870	-1.984696	C	-4.6414620	-1.6601440	1.1447750
C	3.2843130	3.5895210	-0.498001	C	-5.0059320	1.2876500	-1.275834	C	-4.0172330	-4.3083980	1.7330230
C	4.6129510	3.7869950	-0.321410	C	-4.8846150	3.9997230	-1.891875	H	-2.0688960	-3.5595170	2.2673950
C	3.2151300	0.0258090	-1.618682	H	-2.7949820	3.6470470	-2.286903	C	-5.5923230	-2.6555520	0.9592620
C	1.8792260	-0.1506610	-1.868597	C	-6.1445250	2.0764120	-1.168215	H	-4.8802240	-0.6349470	0.9033680
C	0.9542730	0.8804090	-1.563697	H	-5.0538260	0.2370910	-1.028752	C	-5.2861720	-3.9802560	1.2566460
C	1.4301390	2.1085580	-1.134009	C	-6.0897370	3.4314360	-1.481054	H	-3.7754900	-5.3375070	1.9678670
H	4.9170930	0.6873010	1.9974080	H	-4.8376430	5.0533020	-2.137773	H	-6.5689310	-2.3971910	0.5702180
H	0.8878150	1.8800590	2.8072380	H	-7.0730450	1.6331360	-0.831726	H	-6.0297510	-4.7542510	1.1138080
H	3.3401380	2.2704340	3.0248330	H	-6.9797540	4.0431800	-1.401729				
H	2.2345900	-4.2107190	0.2568320	C	-3.2274850	-1.4746230	-2.181471				

SCF Done: E(UM052X) = -2526.93306246 A.U.
Low frequencies --- -9.7982 -7.5752 -3.2015 -0.0013 -0.0005 0.0010
Low frequencies --- 11.1595 16.4541 22.0572

Zero-point correction = 0.797351 (Hartree/Particle)
Thermal correction to Energy = 0.843041
Thermal correction to Enthalpy = 0.843985
Thermal correction to Gibbs Free Energy = 0.717790
Sum of electronic and zero-point Energies = -2526.135712
Sum of electronic and thermal Energies = -2526.090022
Sum of electronic and thermal Enthalpies = -2526.089078
Sum of electronic and thermal Free Energies = -2526.215273

Excitation energies and oscillator strengths:

Excited State 1: 1.424-A 1.1662 eV 1063.10 nm *f*=0.0001 <S**2>=0.257
212A -> 213A 0.69501
212B -> 213B 0.71015
This state for optimization and/or second-order correction.
Total Energy, E(TD-HF/TD-KS) = -2526.60943812
Copying the excited state density for this state as the 1-particle RhoCI density.

Excited State 2: 1.197-A 1.2813 eV 967.67 nm *f*=0.0189 <S**2>=0.108
212A -> 213A 0.70810

```
      212B -> 213B         -0.69232

Excited State   3:   2.530-A     1.6644 eV   744.92 nm   f=0.0098   <S**2>=1.350
      209A -> 213A         -0.11854
      210A -> 213A          0.10824
      211A -> 213A          0.55425
      209B -> 213B          0.20660
      210B -> 213B          0.11980
      211B -> 213B          0.70561
      211B -> 215B          0.10276

Excited State   4:   2.177-A     1.7378 eV   713.45 nm   f=0.0692   <S**2>=0.935
      209A -> 213A         -0.19012
      210A -> 213A         -0.25315
      211A -> 213A          0.74784
      209B -> 213B         -0.13154
      211B -> 213B         -0.50626

Excited State   5:   2.552-A     1.8462 eV   671.58 nm   f=0.0240   <S**2>=1.379
      210A -> 213A          0.51757
      208B -> 213B          0.16946
      210B -> 213B          0.71936
      211B -> 213B         -0.29521

Excited State   6:   2.177-A     1.9069 eV   650.18 nm   f=0.0806   <S**2>=0.934
      207A -> 213A          0.11276
      210A -> 213A          0.68189
      210A -> 214A          0.10537
      211A -> 213A          0.10340
      210B -> 213B         -0.62188
      211B -> 213B         -0.15183
      212B -> 214B          0.13384

Excited State   7:   2.487-A     1.9824 eV   625.42 nm   f=0.0059   <S**2>=1.296
      195A -> 213A          0.10674
      205A -> 213A          0.25858
      207A -> 213A          0.58609
      208A -> 213A          0.12184
      209A -> 213A          0.18894
      210A -> 213A         -0.14603
      211A -> 213A          0.10270
      205B -> 213B         -0.22063
      207B -> 213B          0.46417
      208B -> 213B          0.33669

Excited State   8:   2.348-A     2.0486 eV   605.22 nm   f=0.0297   <S**2>=1.128
      205A -> 213A         -0.24789
      207A -> 213A         -0.40033
      209A -> 213A         -0.36037
      211A -> 213A         -0.15400
      195B -> 213B          0.11534
      205B -> 213B         -0.30708
      207B -> 213B          0.45818
      208B -> 213B          0.33366
      209B -> 213B         -0.16695
      212B -> 214B          0.10296

Excited State   9:   2.467-A     2.0883 eV   593.71 nm   f=0.0457   <S**2>=1.271
      208A -> 213A         -0.10664
      210A -> 213A         -0.22067
      211A -> 215A         -0.10104
      212A -> 214A         -0.19009
      206B -> 213B         -0.11748
      209B -> 213B          0.82032
      211B -> 213B         -0.29392
      211B -> 215B          0.11083
```

Excited State 10: 2.697-A 2.1086 eV 588.00 nm *f*=0.0142 <S**2>=1.568
205A -> 213A -0.17963
207A -> 213A -0.37263
209A -> 213A 0.56760
209A -> 214A 0.10155
210A -> 213A 0.20289
210A -> 214A -0.14791
211A -> 213A 0.18503
211A -> 215A 0.21416
212A -> 214A 0.12329
205B -> 213B -0.12000
207B -> 213B 0.15792
208B -> 213B 0.11921
209B -> 213B 0.20953
210B -> 214B 0.12806
211B -> 215B -0.21623
212B -> 214B -0.23179

Excited State 11: 2.856-A 2.1851 eV 567.41 nm *f*=0.0132 <S**2>=1.789
206A -> 213A 0.13175
207A -> 213A -0.14966
208A -> 213A 0.17495
209A -> 213A 0.57835
210A -> 213A -0.14730
210A -> 214A 0.19500
210A -> 216A -0.10234
211A -> 215A -0.28708
212A -> 214A -0.12882
212A -> 215A -0.12306
206B -> 213B 0.10027
207B -> 213B -0.10670
208B -> 213B 0.10314
209B -> 213B -0.24977
210B -> 214B -0.20794
210B -> 216B 0.12168
211B -> 215B 0.27452
212B -> 214B 0.16803

Excited State 12: 2.359-A 2.3020 eV 538.59 nm *f*=0.0286 <S**2>=1.142
198A -> 213A -0.26294
199A -> 213A 0.16159
201A -> 213A -0.14302
202A -> 213A -0.17290
203A -> 213A 0.12793
204A -> 213A -0.14661
206A -> 213A 0.16569
208A -> 213A 0.71126
209A -> 213A -0.29603
210A -> 213A 0.16665
207B -> 213B -0.10704
208B -> 213B 0.14491
209B -> 213B 0.17522

Excited State 13: 2.312-A 2.3093 eV 536.90 nm *f*=0.0040 <S**2>=1.087
208A -> 213A -0.16976
197B -> 213B 0.17276
198B -> 213B 0.21014
199B -> 213B 0.32747
200B -> 213B -0.35037
201B -> 213B 0.49601
203B -> 213B -0.13116
204B -> 213B 0.42086
205B -> 213B -0.12843
206B -> 213B 0.31819
208B -> 213B 0.16147

209B -> 213B 0.12350

Excited State 14: 2.364-A 2.4169 eV 512.98 nm *f*=0.0010 <S**2>=1.147
197A -> 213A 0.18336
198A -> 213A 0.43702
199A -> 213A -0.25301
201A -> 213A 0.22605
202A -> 213A 0.12136
203A -> 213A -0.20424
204A -> 213A 0.26136
205A -> 213A 0.16832
208A -> 213A 0.25604
201B -> 213B -0.10982
204B -> 213B -0.10656
207B -> 213B -0.31257
208B -> 213B 0.47804
210B -> 213B -0.11152

Excited State 15: 2.418-A 2.4409 eV 507.95 nm *f*=0.0055 <S**2>=1.211
197A -> 213A -0.11491
198A -> 213A -0.27637
199A -> 213A 0.15616
201A -> 213A -0.14687
203A -> 213A 0.12284
204A -> 213A -0.17237
205A -> 213A -0.10313
208A -> 213A -0.30477
211A -> 214A 0.10202
212A -> 214A 0.22009
212A -> 215A 0.11664
201B -> 213B -0.10918
204B -> 213B -0.10192
207B -> 213B -0.33466
208B -> 213B 0.53560
209B -> 213B 0.11265
209B -> 215B -0.10330
210B -> 213B -0.10930
211B -> 214B -0.11157
212B -> 214B 0.16921

Excited State 16: 2.972-A 2.5715 eV 482.15 nm *f*=0.1610 <S**2>=1.959
206A -> 213A 0.14530
208A -> 213A 0.29665
209A -> 215A 0.17506
211A -> 214A 0.26336
211A -> 215A -0.14963
211A -> 216A 0.11689
212A -> 214A 0.21510
212A -> 215A 0.23110
205B -> 213B 0.10235
206B -> 213B 0.23858
207B -> 213B 0.27029
208B -> 213B -0.18002
209B -> 213B 0.17345
209B -> 215B -0.22306
211B -> 214B -0.27667
211B -> 216B -0.12844
212B -> 214B 0.17032
212B -> 215B -0.18844

Excited State 17: 2.481-A 2.6760 eV 463.32 nm *f*=0.0349 <S**2>=1.289
204A -> 213A -0.11402
205A -> 213A 0.43774
207A -> 213A -0.28304
208A -> 213A -0.17695
211A -> 214A -0.10728

197B -> 213B -0.12396
205B -> 213B 0.47458
206B -> 213B 0.39826
207B -> 213B 0.33440
208B -> 213B 0.12185
211B -> 214B 0.10066

Excited State 18: 2.519-A 2.7082 eV 457.82 nm *f*=0.0487 <S**2>=1.336
203A -> 213A -0.11708
204A -> 213A 0.15035
205A -> 213A -0.33302
206A -> 213A 0.43804
207A -> 213A 0.29307
208A -> 213A -0.11703
197B -> 213B -0.10535
204B -> 213B -0.39277
205B -> 213B -0.17497
206B -> 213B 0.47565

Excited State 19: 2.282-A 2.7354 eV 453.26 nm *f*=0.0033 <S**2>=1.051
204A -> 213A 0.10968
205A -> 213A -0.48806
207A -> 213A 0.26862
204B -> 213B 0.46672
205B -> 213B 0.51732
206B -> 213B -0.11634
207B -> 213B 0.19387
208B -> 213B 0.13902

Excited State 20: 2.349-A 2.7426 eV 452.07 nm *f*=0.0105 <S**2>=1.130
203A -> 213A -0.12253
204A -> 213A 0.17635
205A -> 213A 0.30612
206A -> 213A 0.70205
208A -> 213A -0.15211
211A -> 214A 0.10149
204B -> 213B 0.28529
206B -> 213B -0.29116
211B -> 215B -0.12684

Excited State 21: 2.867-A 2.7684 eV 447.86 nm *f*=0.0085 <S**2>=1.805
203A -> 213A -0.17836
204A -> 213A 0.19994
211A -> 215A 0.27041
212A -> 214A -0.47691
212A -> 215A 0.13460
205B -> 213B 0.19762
210B -> 215B -0.16334
211B -> 215B -0.25119
212B -> 214B 0.47927
212B -> 215B 0.18233

Excited State 22: 2.419-A 2.7870 eV 444.87 nm *f*=0.0011 <S**2>=1.213
197A -> 213A 0.11213
198A -> 213A 0.27219
199A -> 213A -0.19617
201A -> 213A 0.22835
202A -> 213A 0.29148
203A -> 213A 0.51926
204A -> 213A -0.49720
206A -> 213A 0.24996
212A -> 214A -0.23892

Excited State 23: 2.430-A 2.7997 eV 442.85 nm *f*=0.0010 <S**2>=1.226
203A -> 213A 0.12181
206A -> 213A -0.14977

211A -> 215A 0.11090
195B -> 213B 0.11215
197B -> 213B -0.11336
198B -> 213B -0.11113
199B -> 213B -0.19792
200B -> 213B 0.19189
201B -> 213B -0.34907
203B -> 213B 0.18150
204B -> 213B 0.50342
205B -> 213B -0.34110
206B -> 213B 0.37415
207B -> 213B -0.10993
208B -> 213B -0.10188
212B -> 214B 0.15277
212B -> 215B 0.11738

Excited State 24: 3.186-A 2.8220 eV 439.36 nm *f*=0.0649 <S**2>=2.287
202A -> 213A -0.20809
203A -> 213A 0.10483
206A -> 213A -0.12431
210A -> 214A 0.38904
210A -> 215A -0.14612
211A -> 215A 0.21807
212A -> 214A 0.33825
212A -> 215A 0.13864
197B -> 213B 0.12762
204B -> 213B -0.16945
206B -> 213B -0.14280
209B -> 216B -0.11417
210B -> 214B -0.38732
211B -> 214B 0.12579
211B -> 215B -0.25135
212B -> 214B 0.19303
212B -> 216B -0.11734

Excited State 25: 3.178-A 2.8615 eV 433.29 nm *f*=0.0084 <S**2>=2.274
210A -> 214A 0.21918
210A -> 215A 0.32213
211A -> 214A -0.31726
211A -> 216A 0.14387
212A -> 215A 0.20549
212A -> 216A 0.15683
206B -> 213B -0.12385
210B -> 215B -0.37677
211B -> 214B 0.11458
211B -> 215B 0.19470
211B -> 216B -0.16209
212B -> 214B -0.21525
212B -> 215B 0.46112

Excited State 26: 2.681-A 2.9184 eV 424.83 nm *f*=0.0258 <S**2>=1.547
209A -> 215A 0.11283
211A -> 214A -0.10948
212A -> 214A -0.29078
212A -> 215A 0.44877
212A -> 216A 0.11285
199B -> 213B 0.13187
200B -> 213B -0.26369
201B -> 213B -0.28061
202B -> 213B -0.12778
203B -> 213B 0.12220
208B -> 213B 0.10679
211B -> 214B 0.20243
212B -> 214B -0.28841
212B -> 215B -0.44077

Excited State 27: 2.498-A 2.9198 eV 424.63 nm *f*=0.0012 <S**2>=1.310
212A -> 214A -0.12366
212A -> 215A 0.26048
193B -> 213B 0.12684
195B -> 213B -0.23211
197B -> 213B -0.12232
198B -> 213B -0.10810
199B -> 213B -0.23335
200B -> 213B 0.50099
201B -> 213B 0.49873
202B -> 213B 0.12668
203B -> 213B -0.10840
211B -> 214B 0.12394
212B -> 214B -0.14190
212B -> 215B -0.25869

Excited State 28: 2.373-A 2.9314 eV 422.95 nm *f*=0.0009 <S**2>=1.157
198A -> 213A 0.13461
199A -> 213A -0.18350
200A -> 213A 0.57344
201A -> 213A -0.54107
203A -> 213A 0.37067
204A -> 213A 0.28235
199B -> 213B 0.10429

Excited State 29: 2.313-A 2.9638 eV 418.33 nm *f*=0.0023 <S**2>=1.088
194A -> 213A 0.19648
195A -> 213A -0.20948
197A -> 213A -0.14880
198A -> 213A -0.28865
200A -> 213A -0.10115
201A -> 213A 0.35117
202A -> 213A 0.12632
203A -> 213A 0.52354
204A -> 213A 0.56419
205A -> 213A 0.11354

Excited State 30: 2.568-A 2.9889 eV 414.82 nm *f*=0.0035 <S**2>=1.399
197A -> 213A -0.24726
200A -> 213A 0.15078
201A -> 213A -0.15893
202A -> 213A 0.59719
203A -> 213A -0.15798
205A -> 213A 0.11771
208A -> 213A 0.10289
211A -> 214A -0.10662
212A -> 214A 0.11537
198B -> 213B -0.10107
201B -> 213B -0.18194
202B -> 213B 0.24359
203B -> 213B -0.37472
211B -> 214B 0.21144
212B -> 214B 0.17372

Excited State 31: 2.344-A 3.0001 eV 413.26 nm *f*=0.0128 <S**2>=1.124
197A -> 213A -0.17374
202A -> 213A 0.34025
203A -> 213A -0.10925
193B -> 213B -0.14625
195B -> 213B 0.35503
196B -> 213B 0.13007
197B -> 213B 0.15530
198B -> 213B 0.22539
200B -> 213B 0.20163
201B -> 213B 0.21799
203B -> 213B 0.61554

212B -> 215B 0.10743

Excited State 32: 2.680-A 3.0051 eV 412.58 nm *f*=0.0580 <S**2>=1.545
195A -> 213A 0.11824
197A -> 213A 0.18577
202A -> 213A -0.20833
210A -> 214A -0.21526
211A -> 214A -0.31414
212A -> 214A 0.12301
193B -> 213B -0.15490
195B -> 213B 0.37508
196B -> 213B 0.15960
197B -> 213B 0.17430
200B -> 213B 0.13800
202B -> 213B 0.26758
210B -> 214B 0.14131
211B -> 214B 0.32058
212B -> 214B 0.24860
212B -> 215B -0.26966

Excited State 33: 2.417-A 3.0417 eV 407.61 nm *f*=0.0351 <S**2>=1.210
210A -> 215A 0.10983
211A -> 214A -0.16925
212A -> 214A 0.16299
212A -> 215A -0.11832
193B -> 213B 0.19523
194B -> 213B -0.10405
195B -> 213B -0.46626
196B -> 213B -0.21724
198B -> 213B 0.10634
199B -> 213B 0.11465
200B -> 213B -0.12630
202B -> 213B 0.20703
203B -> 213B 0.55827
205B -> 213B -0.14366
211B -> 214B 0.21240
212B -> 214B 0.15888
212B -> 215B -0.12285

Excited State 34: 2.355-A 3.0594 eV 405.25 nm *f*=0.0128 <S**2>=1.137
202A -> 213A -0.19766
210A -> 215A -0.11112
211A -> 214A 0.15627
212A -> 214A -0.13228
201B -> 213B -0.14596
202B -> 213B 0.84294
211B -> 214B -0.19381
212B -> 214B -0.12953

Excited State 35: 2.382-A 3.0853 eV 401.85 nm *f*=0.0101 <S**2>=1.168
191A -> 213A 0.13428
192A -> 213A 0.18486
194A -> 213A -0.51141
195A -> 213A 0.58094
196A -> 213A 0.16934
197A -> 213A 0.16246
198A -> 213A -0.14158
200A -> 213A -0.13063
201A -> 213A 0.14155
202A -> 213A 0.21224
203A -> 213A 0.14361
204A -> 213A 0.18382
205A -> 213A -0.17371

Excited State 36: 2.610-A 3.1105 eV 398.60 nm *f*=0.0699 <S**2>=1.453
210A -> 214A -0.27974

210A -> 215A -0.42379
211A -> 214A -0.41308
212A -> 215A 0.42933
197B -> 213B 0.10166
210B -> 215B 0.14589
211B -> 214B -0.30719
211B -> 215B 0.18415
211B -> 216B 0.10932
212B -> 214B 0.20105
212B -> 215B 0.22732

Excited State 37: 2.356-A 3.1466 eV 394.02 nm *f*=0.0018 <S**2>=1.138
198A -> 213A -0.30639
199A -> 213A -0.12580
200A -> 213A 0.65226
201A -> 213A 0.48955
202A -> 213A -0.14051
203A -> 213A -0.18217
204A -> 213A -0.13420
197B -> 213B -0.21980

Excited State 38: 2.622-A 3.1647 eV 391.77 nm *f*=0.0190 <S**2>=1.468
194A -> 213A -0.10511
196A -> 213A 0.11897
197A -> 213A -0.21439
200A -> 213A 0.22469
201A -> 213A 0.23162
202A -> 213A -0.12565
212A -> 214A -0.23093
212A -> 215A -0.13672
212A -> 217A -0.16890
194B -> 213B -0.10772
195B -> 213B -0.15229
196B -> 213B -0.14324
197B -> 213B 0.50834
198B -> 213B 0.12236
200B -> 213B 0.17489
201B -> 213B -0.16789
206B -> 213B 0.17136
209B -> 214B -0.10071

Excited State 39: 2.663-A 3.2166 eV 385.45 nm *f*=0.0043 <S**2>=1.522
197A -> 213A 0.25727
198A -> 213A -0.13206
202A -> 213A 0.10916
212A -> 214A 0.11870
212A -> 215A 0.10742
212A -> 216A -0.11235
198B -> 213B 0.43326
199B -> 213B 0.23075
200B -> 213B 0.33595
201B -> 213B -0.19397
203B -> 213B -0.14495
209B -> 214B 0.12664
210B -> 215B 0.21430
211B -> 214B 0.21307
212B -> 215B 0.14897
212B -> 217B -0.21572

Excited State 40: 2.724-A 3.2241 eV 384.56 nm *f*=0.0214 <S**2>=1.606
209A -> 215A -0.10127
209A -> 217A -0.11172
210A -> 215A 0.16471
212A -> 216A 0.21158
212A -> 217A 0.30181
192B -> 213B -0.12275

197B -> 213B	-0.10988
198B -> 213B	0.40732
199B -> 213B	0.23313
200B -> 213B	0.27157
201B -> 213B	-0.11008
203B -> 213B	-0.10887
209B -> 215B	0.10385
210B -> 215B	-0.16318
211B -> 214B	-0.11732
211B -> 215B	0.14871
212B -> 217B	0.33918

Table S4. Standard Orientation of the Optimized Geometry for 9H-BR.

	X	Y	Z		X	Y	Z		X	Y	Z
C	2.0327350	-3.7823210	-1.7079760	C	6.9776810	0.2223010	-0.5501280	H	-2.8741800	3.8619230	2.2070420
H	1.7038610	-4.8139010	-1.7305770	C	6.1625880	-1.9855810	0.9723620	C	-5.4285700	-3.3145280	2.6791480
C	1.0991870	-2.7707500	-1.6503680	C	3.4917330	-2.3220230	1.7352070	H	-6.5855050	-1.8236910	3.7112330
H	0.0448710	-2.9972000	-1.6352930	C	1.6078120	-0.2265280	1.5346130	H	-4.0606880	-4.5695530	1.5903040
C	0.5387310	-0.3230220	-1.7092510	C	2.4890920	2.4635400	1.3089120	C	-6.1893900	3.9323530	1.4616340
C	1.0233640	0.9520450	-1.9210530	C	5.1539290	3.1079560	1.7835750	H	-7.3250910	2.2354030	0.7871340
H	0.3137050	1.7510520	-2.0815190	C	3.8104370	-2.1658380	-1.7304420	H	-4.7967180	5.4327810	2.1237500
C	2.8253950	2.5917650	-2.1977980	C	2.8837320	-1.1085630	-1.5994240	H	-6.1026520	-4.1248970	2.9271500
H	2.0769030	3.3204280	-2.4842820	C	3.3319080	0.2752460	-1.5331680	H	-7.0284300	4.6152540	1.4159790
C	4.1484760	2.8955300	-2.2011980	C	4.6699660	0.7019790	-1.2103850	C	-0.9016770	-0.4314370	-1.6558650
H	4.5021460	3.8671260	-2.5238590	C	5.6022170	-0.0424510	-0.3916420	N	-1.6586000	0.7087690	-1.7669490
C	6.4839310	2.2862740	-1.7016260	C	5.2093470	-1.0119760	0.6090580	N	-1.6378620	-1.5742700	-1.5621370
H	6.7894590	3.2329560	-2.1307030	C	3.9006910	-1.0706900	1.2327390	C	-2.9067720	0.2971630	-1.7695570
C	7.4013080	1.3998350	-1.2327260	C	2.9838450	0.0419340	1.3629600	C	-2.8995680	-1.1722500	-1.6178420
H	8.4633450	1.6001620	-1.3093910	C	3.4387480	1.4211260	1.4381280	C	-3.9916140	1.2545640	-2.0125840
C	7.9434770	-0.6787760	-0.0102950	C	4.7693770	1.7858880	1.7419760	C	-3.9993550	-2.1294400	-1.5128690
H	8.9939660	-0.4776070	-0.1824070	H	6.1823180	3.3537250	2.0165250	C	-5.1637430	0.9068900	-2.6943230
C	7.5389810	-1.7981760	0.6442440	H	5.4942060	1.0188040	1.9685570	C	-3.7969060	2.5876470	-1.6230340
H	8.2539780	-2.5401700	0.9791140	H	4.8621560	-1.9346460	-1.8148770	C	-5.2558170	-1.7699160	-1.0078480
C	5.7528020	-3.1669910	1.6569980	H	4.1319760	-4.2678450	-1.8871730	C	-3.7641780	-3.4686830	-1.8564340
H	6.4992720	-3.9169130	1.8902210	C	-0.7948430	0.7033180	1.4677710	C	-6.1344120	1.8687250	-2.9524760
C	4.4393000	-3.3691020	1.9339820	N	-1.4434110	-0.4811050	1.6010280	H	-5.3037680	-0.1072840	-3.0440510
H	4.0929320	-4.3012540	2.3637530	N	-1.6304120	1.7951260	1.4492150	C	-4.7691240	3.5424950	-1.8809610
C	2.1253850	-2.5349540	2.0480940	C	-2.7252550	-0.1705710	1.7114240	H	-2.8831110	2.8450780	-1.1038590
H	1.8198630	-3.5157230	2.3925020	C	-2.8425230	1.2966920	1.5897450	C	-6.2561400	-2.7220290	-0.8632540
C	1.2013510	-1.5552490	1.8616440	C	-3.6987710	-1.2176650	2.0235460	H	-5.4388330	-0.7476050	-0.7103700
H	0.1551580	-1.7551420	2.0131680	C	-4.0207030	2.1671830	1.5810310	C	-4.7680700	-4.4162790	-1.7165680
C	0.6461240	0.8640810	1.4207860	C	-4.8458470	-0.9762660	2.7902220	H	-2.7881390	-3.7418420	-2.2339930
C	1.1085820	2.1500440	1.2427070	C	-3.4147190	-2.5297630	1.6127370	C	-5.9436300	3.1847980	-2.5409140
H	0.3875700	2.9472860	1.1238750	C	-5.2836280	1.7170810	1.1767850	H	-7.0340320	1.5909020	-3.4870250
C	2.9084670	3.8149100	1.3251600	C	-3.8565120	3.5195460	1.9116450	H	-4.6182310	4.5627340	-1.5523440
H	2.1604590	4.5875540	1.1920460	C	-5.7058670	-2.0204760	3.1114050	C	-6.0173270	-4.0463530	-1.2198420
C	4.2256250	4.1373410	1.5392610	H	-5.0484440	0.0206220	3.1579880	H	-7.2157520	-2.4323910	-0.4547970
H	4.5423640	5.1722460	1.5601130	C	-4.2775250	-3.5665130	1.9342610	H	-4.5786340	-5.4451290	-1.9959850
C	3.3989760	-3.4779650	-1.7829190	H	-2.5236270	-2.7058050	1.0238600	H	-6.7035270	3.9303530	-2.7396600
C	1.4950940	-1.4160290	-1.6313860	C	-6.3593750	2.5941680	1.1191080	H	-6.7983110	-4.7879540	-1.1080120
C	2.4015770	1.2659890	-1.8894450	H	-5.4162550	0.6851880	0.8857620				
C	5.0979230	1.9554780	-1.7052270	C	-4.9337990	4.3924870	1.8566290				

SCF Done: E(UM052X) = -2834.19527016 A.U.
Low frequencies --- -7.3786 -5.5616 -3.9166 -0.0010 -0.0007 0.0003
Low frequencies --- 10.6728 23.9414 32.2082

Zero-point correction = 0.893221 (Hartree/Particle)
Thermal correction to Energy = 0.943952
Thermal correction to Enthalpy = 0.944896
Thermal correction to Gibbs Free Energy = 0.810277
Sum of electronic and zero-point Energies = -2833.302049
Sum of electronic and thermal Energies = -2833.251319
Sum of electronic and thermal Enthalpies = -2833.250374
Sum of electronic and thermal Free Energies = -2833.384993

Excitation energies and oscillator strengths:

Excited State 1: 1.694-A 0.9833 eV 1260.88 nm *f*=0.0034 <S**2>=0.467
238A -> 239A 0.79987
238B -> 239B 0.58283
This state for optimization and/or second-order correction.

Total Energy, E(TD-HF/TD-KS) = -2833.83213159
Copying the excited state density for this state as the 1-particle RhoCI density.

Excited State 2: 1.119-A 1.3009 eV 953.07 nm *f*=0.0503 <S**2>=0.063
238A -> 239A -0.58341
238B -> 239B 0.80345

Excited State 3: 2.597-A 1.5035 eV 824.66 nm *f*=0.0019 <S**2>=1.436
235A -> 239A -0.26676
236A -> 239A -0.37145
237A -> 239A 0.51951
235B -> 239B 0.50248
237B -> 239B 0.44323

Excited State 4: 2.548-A 1.6489 eV 751.92 nm *f*=0.0417 <S**2>=1.373
236A -> 239A 0.83599
236B -> 239B 0.11102
237B -> 239B 0.44466

Excited State 5: 2.142-A 1.7059 eV 726.78 nm *f*=0.0109 <S**2>=0.897
235A -> 239A 0.14788
236A -> 239A -0.19871
237A -> 239A -0.29114
235B -> 239B -0.27660
236B -> 239B -0.54131
237B -> 239B 0.64966

Excited State 6: 2.372-A 1.7447 eV 710.64 nm *f*=0.1422 <S**2>=1.157
236A -> 239A 0.19620
237A -> 239A 0.70099
238A -> 240A 0.16663
235B -> 239B -0.32621
236B -> 239B -0.48035
237B -> 239B -0.19648

Excited State 7: 2.123-A 1.8217 eV 680.58 nm *f*=0.0214 <S**2>=0.877
236A -> 239A 0.18115
237A -> 239A -0.22438
233B -> 239B -0.11734
234B -> 239B -0.11245
235B -> 239B 0.64107
236B -> 239B -0.59479
237B -> 239B -0.30931

Excited State 8: 2.667-A 1.9432 eV 638.03 nm *f*=0.0007 <S**2>=1.528
228A -> 239A -0.12391
230A -> 239A 0.14937
232A -> 239A 0.16219
233A -> 239A 0.64840
235A -> 239A -0.15886
230B -> 239B 0.10705
233B -> 239B -0.52917
236B -> 239B 0.10302

Excited State 9: 2.164-A 1.9797 eV 626.28 nm *f*=0.0446 <S**2>=0.921
235A -> 239A 0.87115
237A -> 239A 0.21323
238A -> 240A -0.11972
233B -> 239B -0.27295
235B -> 239B 0.15061
236B -> 239B 0.11678

Excited State 10: 3.209-A 2.0099 eV 616.86 nm *f*=0.0166 <S**2>=2.324
232A -> 239A -0.10816
233A -> 239A -0.23163
234A -> 240A 0.14270

235A -> 240A 0.11458
236A -> 241A 0.28341
236A -> 243A 0.10530
237A -> 240A 0.19748
237A -> 241A 0.25529
237A -> 242A 0.10574
238A -> 240A 0.25096
238A -> 241A -0.13161
234B -> 239B 0.22832
234B -> 240B -0.14800
235B -> 239B 0.17482
235B -> 241B -0.14236
236B -> 239B 0.16144
236B -> 240B 0.21169
236B -> 244B -0.12497
237B -> 241B 0.37075
238B -> 240B -0.24217

Excited State 11: 2.191-A 2.0817 eV 595.60 nm *f*=0.0508 <S**2>=0.951
228A -> 239A -0.15031
230A -> 239A 0.21369
231A -> 239A -0.13885
232A -> 239A 0.14373
233A -> 239A 0.44560
234A -> 239A -0.11180
235A -> 239A 0.21306
220B -> 239B 0.10397
228B -> 239B 0.15535
230B -> 239B -0.21695
233B -> 239B 0.57736
234B -> 239B 0.21550
235B -> 239B 0.15571

Excited State 12: 2.611-A 2.1421 eV 578.80 nm *f*=0.0017 <S**2>=1.454
232A -> 239A 0.12625
234A -> 239A 0.53857
232B -> 239B 0.13588
233B -> 239B -0.12103
234B -> 239B 0.73099

Excited State 13: 1.985-A 2.2205 eV 558.37 nm *f*=0.0152 <S**2>=0.735
232A -> 239A 0.11535
234A -> 239A 0.76894
233B -> 239B 0.11252
234B -> 239B -0.50588

Excited State 14: 2.336-A 2.3266 eV 532.89 nm *f*=0.0010 <S**2>=1.114
222A -> 239A 0.24015
223A -> 239A -0.28424
224A -> 239A -0.16325
225A -> 239A 0.57512
227A -> 239A 0.17783
229A -> 239A 0.48626
230A -> 239A -0.18899
231A -> 239A 0.29956
234A -> 239A -0.18481

Excited State 15: 2.400-A 2.3433 eV 529.11 nm *f*=0.0089 <S**2>=1.190
238A -> 240A 0.14301
222B -> 239B 0.39940
223B -> 239B -0.20058
226B -> 239B 0.54605
229B -> 239B -0.31824
230B -> 239B 0.45748
231B -> 239B -0.15879
233B -> 239B 0.11720

```
Excited State   16:   2.985-A      2.3837 eV   520.13 nm   f=0.1061   <S**2>=1.978
     231A -> 239A         0.16410
     232A -> 239A         0.22583
     234A -> 241A        -0.10926
     235A -> 239A         0.17484
     235A -> 240A         0.18529
     235A -> 241A         0.10185
     236A -> 241A        -0.23782
     237A -> 239A        -0.12004
     238A -> 240A         0.42513
     238A -> 242A        -0.14216
     222B -> 239B        -0.11163
     226B -> 239B        -0.15244
     230B -> 239B        -0.18139
     232B -> 239B         0.13810
     234B -> 239B        -0.17501
     235B -> 239B         0.14429
     235B -> 240B         0.22260
     236B -> 239B         0.11894
     236B -> 241B        -0.26074
     237B -> 244B         0.10113
     238B -> 242B         0.12373

Excited State   17:   2.767-A      2.5094 eV   494.07 nm   f=0.0336   <S**2>=1.664
     228A -> 239A        -0.11031
     230A -> 239A         0.11682
     231A -> 239A         0.14399
     232A -> 239A         0.52706
     233A -> 239A        -0.25852
     234A -> 239A        -0.14322
     236A -> 239A         0.11948
     236A -> 241A         0.11958
     237A -> 241A         0.18492
     238A -> 240A        -0.35028
     232B -> 239B         0.34621
     237B -> 241B         0.24312
     238B -> 240B         0.25637
     238B -> 241B         0.14345

Excited State   18:   2.644-A      2.5369 eV   488.73 nm   f=0.0064   <S**2>=1.497
     231A -> 239A        -0.29372
     233A -> 239A        -0.13945
     237A -> 241A        -0.14196
     238A -> 240A         0.22626
     238A -> 241A         0.10578
     231B -> 239B         0.21193
     232B -> 239B         0.66593
     234B -> 239B        -0.10621
     236B -> 241B         0.11381
     237B -> 240B        -0.10319
     238B -> 240B        -0.26899
     238B -> 242B        -0.12176

Excited State   19:   2.251-A      2.5857 eV   479.50 nm   f=0.0068   <S**2>=1.016
     225A -> 239A        -0.10538
     227A -> 239A        -0.10909
     228A -> 239A         0.11661
     229A -> 239A        -0.14952
     230A -> 239A        -0.33779
     231A -> 239A         0.34674
     232A -> 239A        -0.34233
     233A -> 239A         0.32951
     238A -> 241A        -0.28318
     231B -> 239B        -0.11414
     232B -> 239B         0.49482
```

233B -> 239B 0.14453

Excited State 20: 2.959-A 2.6092 eV 475.19 nm *f*=0.0142 <S**2>=1.939
228A -> 239A 0.11406
230A -> 239A -0.15890
232A -> 239A -0.15000
233A -> 239A 0.14828
234A -> 241A 0.12788
235A -> 241A 0.15674
236A -> 241A -0.12806
237A -> 240A 0.20649
237A -> 241A 0.19435
238A -> 240A -0.13885
238A -> 241A 0.65028
231B -> 239B 0.11739
236B -> 241B -0.22915
237B -> 240B -0.26294
238B -> 241B 0.28531

Excited State 21: 2.744-A 2.6280 eV 471.78 nm *f*=0.0258 <S**2>=1.633
227A -> 239A -0.11592
229A -> 239A -0.14981
230A -> 239A -0.10777
231A -> 239A 0.35102
232A -> 239A 0.34231
237A -> 241A -0.21677
238A -> 240A 0.20471
238A -> 241A 0.19039
226B -> 239B -0.11977
229B -> 239B 0.24314
230B -> 239B 0.26378
231B -> 239B -0.28186
233B -> 239B 0.16268
236B -> 241B 0.20085
237B -> 241B -0.10674
238B -> 240B -0.23584

Excited State 22: 2.325-A 2.6600 eV 466.11 nm *f*=0.0189 <S**2>=1.102
228A -> 239A -0.10963
229A -> 239A -0.17319
231A -> 239A 0.45168
236A -> 240A -0.10496
237A -> 241A -0.10579
238A -> 240A 0.14943
226B -> 239B 0.11809
228B -> 239B 0.25486
229B -> 239B -0.36567
230B -> 239B -0.38167
231B -> 239B 0.38750
233B -> 239B -0.29595

Excited State 23: 2.416-A 2.7078 eV 457.87 nm *f*=0.0279 <S**2>=1.209
227A -> 239A -0.11749
228A -> 239A 0.29051
229A -> 239A -0.19801
230A -> 239A -0.29924
231A -> 239A -0.11107
232A -> 239A 0.34405
233A -> 239A 0.10963
238A -> 240A 0.15095
228B -> 239B -0.25564
229B -> 239B 0.10088
230B -> 239B 0.11357
231B -> 239B 0.52332
232B -> 239B -0.14989
233B -> 239B 0.11651

238B -> 240B 0.22923

Excited State 24: 3.027-A 2.7208 eV 455.69 nm *f*=0.0255 <S**2>=2.041
228A -> 239A -0.14604
229A -> 239A 0.15118
230A -> 239A 0.15410
232A -> 239A -0.17049
236A -> 241A 0.30646
237A -> 240A -0.35741
238A -> 240A 0.19951
238A -> 241A 0.38034
234B -> 240B 0.10328
235B -> 241B -0.17699
236B -> 240B -0.12090
236B -> 241B 0.12236
237B -> 240B 0.28397
237B -> 241B 0.13378
238B -> 240B 0.39571

Excited State 25: 2.455-A 2.7371 eV 452.98 nm *f*=0.0095 <S**2>=1.257
225A -> 239A -0.15098
228A -> 239A -0.23828
229A -> 239A 0.28495
230A -> 239A 0.21088
231A -> 239A 0.16107
232A -> 239A -0.19255
237A -> 240A 0.12318
238A -> 241A -0.17229
227B -> 239B 0.18544
228B -> 239B 0.11360
229B -> 239B 0.41654
230B -> 239B 0.29353
231B -> 239B 0.47356

Excited State 26: 2.954-A 2.7915 eV 444.15 nm *f*=0.0098 <S**2>=1.931
222A -> 239A -0.12537
223A -> 239A 0.10691
225A -> 239A -0.24562
227A -> 239A 0.16844
229A -> 239A 0.26776
231A -> 239A 0.17789
235A -> 240A -0.10791
236A -> 240A 0.37549
237A -> 240A 0.11092
238A -> 241A -0.17180
238A -> 242A -0.11689
228B -> 239B -0.24261
230B -> 239B -0.23497
232B -> 239B -0.12741
236B -> 240B -0.24595
237B -> 240B -0.31642
237B -> 241B 0.12354
238B -> 241B 0.15901

Excited State 27: 2.487-A 2.8012 eV 442.62 nm *f*=0.0045 <S**2>=1.296
222A -> 239A 0.11540
224A -> 239A -0.12975
225A -> 239A 0.42096
227A -> 239A -0.32992
229A -> 239A -0.43153
230A -> 239A 0.43877
231A -> 239A 0.14387
232A -> 239A -0.21051
236A -> 240A 0.20783
229B -> 239B 0.12234
236B -> 240B -0.14065

237B -> 240B -0.14318

Excited State 28: 2.404-A 2.8088 eV 441.42 nm *f*=0.0334 <S**2>=1.195
228A -> 239A 0.11750
232A -> 239A 0.11245
235A -> 241A 0.11541
238A -> 240A -0.16572
238A -> 241A 0.16030
222B -> 239B 0.26930
226B -> 239B 0.37770
227B -> 239B 0.21615
229B -> 239B 0.48552
230B -> 239B -0.36203
234B -> 241B -0.11710
238B -> 240B -0.15825
238B -> 241B -0.33035

Excited State 29: 2.383-A 2.8264 eV 438.67 nm *f*=0.0339 <S**2>=1.170
229A -> 239A -0.12221
230A -> 239A -0.19797
231A -> 239A -0.16118
235A -> 241A -0.13779
237A -> 241A -0.12157
238A -> 240A 0.16276
238A -> 241A -0.11901
222B -> 239B 0.10422
224B -> 239B 0.12535
226B -> 239B 0.17338
228B -> 239B 0.27937
229B -> 239B 0.28598
230B -> 239B -0.19598
231B -> 239B -0.19381
234B -> 241B 0.16912
237B -> 240B 0.10241
238B -> 241B 0.59369

Excited State 30: 2.810-A 2.8319 eV 437.82 nm *f*=0.0078 <S**2>=1.724
224A -> 239A -0.20519
227A -> 239A 0.20328
228A -> 239A -0.20195
229A -> 239A -0.12357
230A -> 239A -0.31520
231A -> 239A -0.12009
236A -> 240A 0.26580
238A -> 240A -0.11535
221B -> 239B 0.17182
222B -> 239B -0.15693
224B -> 239B 0.10583
228B -> 239B 0.43268
230B -> 239B 0.16453
231B -> 239B 0.10234
232B -> 239B -0.10811
233B -> 239B -0.10419
235B -> 240B 0.14758
235B -> 241B -0.10789
236B -> 240B -0.12495
238B -> 241B -0.18572

Excited State 31: 2.285-A 2.8958 eV 428.15 nm *f*=0.0346 <S**2>=1.055
222A -> 239A 0.21540
223A -> 239A -0.19654
224A -> 239A -0.18280
227A -> 239A 0.45927
228A -> 239A -0.42490
229A -> 239A -0.35857
230A -> 239A -0.10768

236A -> 240A -0.12228
222B -> 239B 0.10850
227B -> 239B -0.12478
228B -> 239B -0.39640

Excited State 32: 2.600-A 2.8980 eV 427.83 nm *f*=0.1279 <S**2>=1.439
231A -> 239A -0.13868
235A -> 240A 0.10346
236A -> 241A 0.11201
237A -> 240A 0.26524
238A -> 240A 0.23351
222B -> 239B 0.11845
227B -> 239B -0.18686
229B -> 239B 0.11846
230B -> 239B -0.10209
231B -> 239B -0.17798
235B -> 240B 0.11721
236B -> 240B 0.13798
236B -> 241B 0.16386
237B -> 240B -0.42276
238B -> 240B 0.43575
238B -> 241B -0.34908

Excited State 33: 2.402-A 2.9380 eV 422.00 nm *f*=0.0004 <S**2>=1.192
226A -> 239A 0.48758
228A -> 239A -0.31243
230A -> 239A -0.14684
237A -> 241A 0.11178
222B -> 239B -0.19820
223B -> 239B 0.19766
224B -> 239B -0.19762
225B -> 239B 0.20839
226B -> 239B 0.21841
227B -> 239B 0.47655
228B -> 239B -0.10656
229B -> 239B -0.17385

Excited State 34: 2.425-A 2.9404 eV 421.66 nm *f*=0.0139 <S**2>=1.221
220A -> 239A 0.10449
223A -> 239A 0.27824
226A -> 239A 0.59293
227A -> 239A -0.10079
228A -> 239A -0.12987
230A -> 239A -0.11201
237A -> 241A -0.13886
238A -> 240A -0.11973
224B -> 239B 0.10994
225B -> 239B 0.20392
227B -> 239B -0.45285
229B -> 239B 0.12539
236B -> 241B -0.11496
237B -> 240B 0.14205
238B -> 241B 0.10870

Excited State 35: 2.826-A 2.9459 eV 420.87 nm *f*=0.0012 <S**2>=1.747
224A -> 239A 0.10477
235A -> 240A -0.22175
236A -> 240A -0.17816
236A -> 241A -0.19078
237A -> 240A -0.31270
237A -> 241A 0.39472
238A -> 240A 0.17932
222B -> 239B 0.10231
226B -> 239B -0.12946
227B -> 239B -0.28985
228B -> 239B 0.14189

229B -> 239B 0.10001
235B -> 241B 0.13568
235B -> 242B 0.13165
236B -> 240B -0.27505
237B -> 240B -0.17257
237B -> 241B 0.22795
238B -> 240B -0.15766
238B -> 241B -0.11364

Excited State 36: 2.333-A 2.9668 eV 417.90 nm *f*=0.0017 <S**2>=1.111
223A -> 239A -0.14119
227A -> 239A 0.29081
228A -> 239A 0.28083
229A -> 239A -0.11576
230A -> 239A 0.21008
231A -> 239A 0.14340
223B -> 239B 0.34723
225B -> 239B 0.62389
226B -> 239B 0.18107
227B -> 239B -0.17241
228B -> 239B 0.20226
229B -> 239B 0.11981

Excited State 37: 2.336-A 2.9849 eV 415.36 nm *f*=0.0085 <S**2>=1.114
221A -> 239A 0.11755
222A -> 239A 0.25441
224A -> 239A 0.13920
225A -> 239A -0.13443
226A -> 239A 0.43439
227A -> 239A 0.38845
228A -> 239A 0.25185
229A -> 239A -0.15684
230A -> 239A 0.21310
231A -> 239A 0.15071
236A -> 240A 0.12929
223B -> 239B -0.22188
225B -> 239B -0.36570
226B -> 239B -0.17273
227B -> 239B 0.10030
228B -> 239B 0.10925

Excited State 38: 2.709-A 3.0008 eV 413.17 nm *f*=0.0182 <S**2>=1.585
221A -> 239A 0.13850
223A -> 239A -0.17404
224A -> 239A 0.26151
226A -> 239A -0.16654
227A -> 239A -0.26273
228A -> 239A -0.30455
230A -> 239A -0.13388
236A -> 240A 0.36200
236A -> 241A 0.11807
238A -> 240A -0.11932
221B -> 239B -0.10343
222B -> 239B 0.22493
225B -> 239B 0.12655
226B -> 239B -0.15022
235B -> 240B 0.15018
236B -> 240B 0.14612
236B -> 241B 0.18528
237B -> 240B 0.12711
238B -> 240B -0.25419
238B -> 242B 0.17676

Excited State 39: 2.628-A 3.0615 eV 404.97 nm *f*=0.0019 <S**2>=1.477
218A -> 239A -0.31135
219A -> 239A 0.44498

220A -> 239A -0.30926
221A -> 239A 0.18789
222A -> 239A 0.13545
227A -> 239A -0.19125
228A -> 239A -0.13404
236A -> 240A -0.14741
218B -> 239B 0.11992
219B -> 239B -0.13743
220B -> 239B 0.25135
226B -> 239B -0.14762
235B -> 240B -0.15237
235B -> 241B -0.11489
236B -> 241B -0.18549
238B -> 242B -0.10910

Excited State 40: 2.281-A 3.0834 eV 402.10 nm *f*=0.0144 <S**2>=1.050
236A -> 240A -0.31972
236A -> 241A -0.19795
237A -> 240A -0.33003
237A -> 241A -0.38868
238A -> 240A -0.13160
234B -> 241B -0.16210
235B -> 240B 0.11245
235B -> 241B -0.16869
236B -> 240B 0.16379
237B -> 240B -0.33844
237B -> 241B 0.47433
238B -> 241B 0.13895

9. References

S1. H. Shinkai, T. Ito, T. Iida, Y. Kitao, H. Yamada and I. Uchida, *J. Med. Chem.* 2000, **43**, 4667−4677.

S2. J. L. Burkhart, , R. Müller and U. Kazmaier, *Eur. J. Org. Chem.* 2011, 3050−3059.

S3. S. Sato, K. Sakata, Y. Hashimoto, H. Takikawa and K. Suzuki, *Angew. Chem. Int. Ed.* 2017, **56**, 12608−12613.

S4. T. Bosanac and C. S. Wilcox, *Org. Lett.* 2004, **6**, 2321−2324.

S5. J. D. Moseley, W. O. Moss, M. J. Welham, C. L. Ancell, J. Banister, S. A. Bowden, G. Norton and M. J. Young, *Org. Proc. Res. Dev.* 2003, **7**, 58−66.

S6. Gaussian09 program (Revision D.01), M. J. Frisch, G. W. Trucks, H. B. Schlegel, G. E. Scuseria, M. A. Robb, J. R. Cheeseman, G. Scalmani, V. Barone, G. A. Petersson, H. Nakatsuji, X. Li, M. Caricato, A. V. Marenich, J. Bloino, B. G. Janesko, R. Gomperts, B. Mennucci, H. P. Hratchian, J. V. Ortiz, A. F. Izmaylov, J. L. Sonnenberg, D. Williams-Young, F. Ding, F. Lipparini, F. Egidi, J. Goings, B. Peng, A. Petrone, T. Henderson, D. Ranasinghe, V. G. Zakrzewski, J. Gao, N. Rega, G. Zheng, W. Liang, M. Hada, M. Ehara, K. Toyota, R. Fukuda, J. Hasegawa, M. Ishida, T. Nakajima, Y. Honda, O. Kitao, H. Nakai, T. Vreven, K. Throssell, J. A. Montgomery, Jr., J. E. Peralta, F. Ogliaro, M. J. Bearpark, J. J. Heyd, E. N. Brothers, K. N. Kudin, V. N. Staroverov, T. A. Keith, R. Kobayashi, J. Normand, K. Raghavachari, A. P. Rendell, J. C. Burant, S. S. Iyengar, J. Tomasi, M. Cossi, J. M. Millam, M. Klene, C. Adamo, R. Cammi, J. W. Ochterski, R. L. Martin, K. Morokuma, O. Farkas, J. B. Foresman, and D. J. Fox, Gaussian, Inc., Wallingford CT, 2016.
